# High-yield syntheses of doubly 2,7-dialkynylpyrene-threaded photostable [8]rotaxanes exhibiting extremely bright circularly polarized luminescence

**DOI:** 10.1039/d5sc06304c

**Published:** 2025-09-26

**Authors:** Kohei Nishioki, Asuka Kimura, Yuki Ohishi, Juri Yamashita, Masahiro Kitamoto, Munetaka Iwamura, Koichi Nozaki, Junya Chiba, Masahiko Inouye

**Affiliations:** a Graduate School of Medicine and Pharmaceutical Sciences, University of Toyama Sugitani Toyama 930-0194 Japan ohishi@pha.u-toyama.ac.jp; b Graduate School of Science and Engineering, University of Toyama Gofuku Toyama 930-8555 Japan

## Abstract

Circularly polarized luminescence (CPL) emitters garner much interest due to their potential applications in chiroptical materials. While many organic CPL emitters have been reported, their preparation often requires low-yield reactions and laborious optical resolution steps. In this study, we demonstrated a cooperative rotaxane approach to synthesize CPL emitters with desirable properties in high yields. We designed doubly 2,7-dialkynylpyrene-threaded [8]rotaxanes with two γ-cyclodextrins and four cucurbit[6]urils as ring components. Although these [8]rotaxanes have heavily complicated interlocked structures, their syntheses were achieved in 67–77% isolated yields as a single isomer utilizing a cooperative capture strategy. These [8]rotaxanes exhibited high luminescence quantum yields *Φ*_lum_ of 0.40–0.47 and large |*g*_lum_| values of 1.1–1.9 × 10^−2^ in an aqueous solution. Considering *ε* = 2.2–2.5 × 10^5^ M^−1^ cm^−1^, the CPL brightness (*B*_CPL_ = *ε* × *Φ*_lum_ × |*g*_lum_|/2) values of 569–836 were as bright as the highest ones of reported organic CPL emitters. Owing to the insulation of the alkynylpyrene cores against outside molecules by the ring components, the [8]rotaxanes showed outstanding photo- and thermal-stability in solutions. The [8]rotaxanes might have the potential to be CPL emitters suitable for future optical materials.

## Abstract 2

[8]Rotaxane-type circularly polarized luminescence (CPL) emitters were synthesized in high yields. These rotaxanes exhibited bright CPL and showed outstanding photo- and thermal-stability.

## Graphical abstract

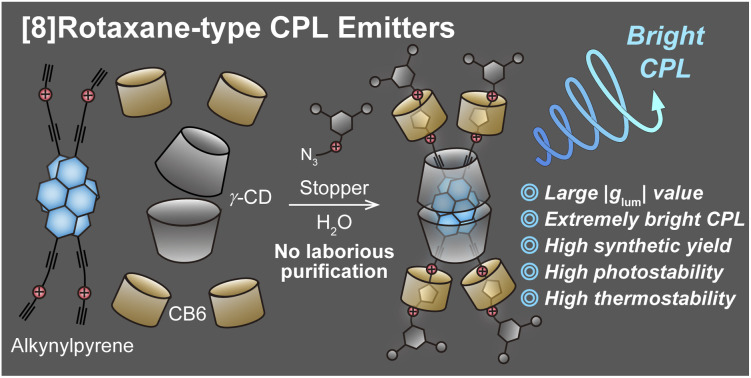

## Introduction

Circularly polarized luminescence (CPL) is the differential emission of right- and left-circularly polarized light.^1^ CPL emitters have the potential to be applied in 3D displays,^2,3^ bioimaging probes,^4^ security inks,^5^ and catalysts for enantioselective synthesis.^6,7^ The performance of CPL emitters is often evaluated by the luminescence dissymmetry factor (*g*_lum_), which quantifies the polarization degree. On the other hand, practical CPL emitters require not only large |*g*_lum_|, but also enough molar absorption coefficient (*ε*) and luminescence quantum yield (*Φ*_lum_). Collectively, CPL brightness (*B*_CPL_ = *ε* × *Φ*_lum_ × |*g*_lum_|/2) is known as a metric that quantifies the overall efficiency of CPL emitters.^8^ Although chiral lanthanide complexes are recognized as a potential CPL emitter due to the large |*g*_lum_| values, their low *Φ*_lum_ ones limit the utility in CPL materials.^9,10^ In addition, lanthanides unevenly distribute on earth and might have political risks. Therefore, in recent years much research has focused on developing purely organic CPL emitters with high *B*_CPL_ values.

In this connection, several π-expanded organic CPL emitters have been reported such as helicene and binaphthyl derivatives with helical and axial chiralities in their own right.^11,12^ However, many of those exhibit low |*g*_lum_| values resulting from their small magnetic dipole transition moment. To overcome this problem, excimers, especially pyrene excimers, drew attention for constructing CPL emitters because of their high |*g*_lum_| values when pairs of pyrenes were connected with chiral backbones.^13–17^ For example, chiral cyclophane-based pyrene excimers have recently been reported as a CPL emitter showing large |*g*_lum_| values.^18,19^ Although the rigid cyclophane-based structures are beneficial for producing a pair of asymmetrically stacking pyrenes, the synthetic yields of macrocyclization are inevitably low. Furthermore, the purification of such molecules needs optical resolution by chiral column chromatography at the final stage of the synthetic scheme. This process necessitates costly investment and laborious examination.

The host–guest strategy has emerged as an easy approach for creating organic CPL emitters from achiral fluorophores, since the complexation induces asymmetric conformation to the achiral guest fluorophores by using chiral hosts.^20–22^ For example, complexes of cyclodextrins (CDs) with achiral guest fluorophores often exhibit circular dichroism and/or CPL signals, reflecting the intrinsic chirality of CDs.^15,23–26^ Although the host–guest strategy has the advantage that CPL materials can be easily prepared by simply mixing chiral hosts and achiral guest fluorophores, these reversible inclusion complexes spontaneously dissociate in dilute solutions, practically limiting their applications.

**Fig. 1:**
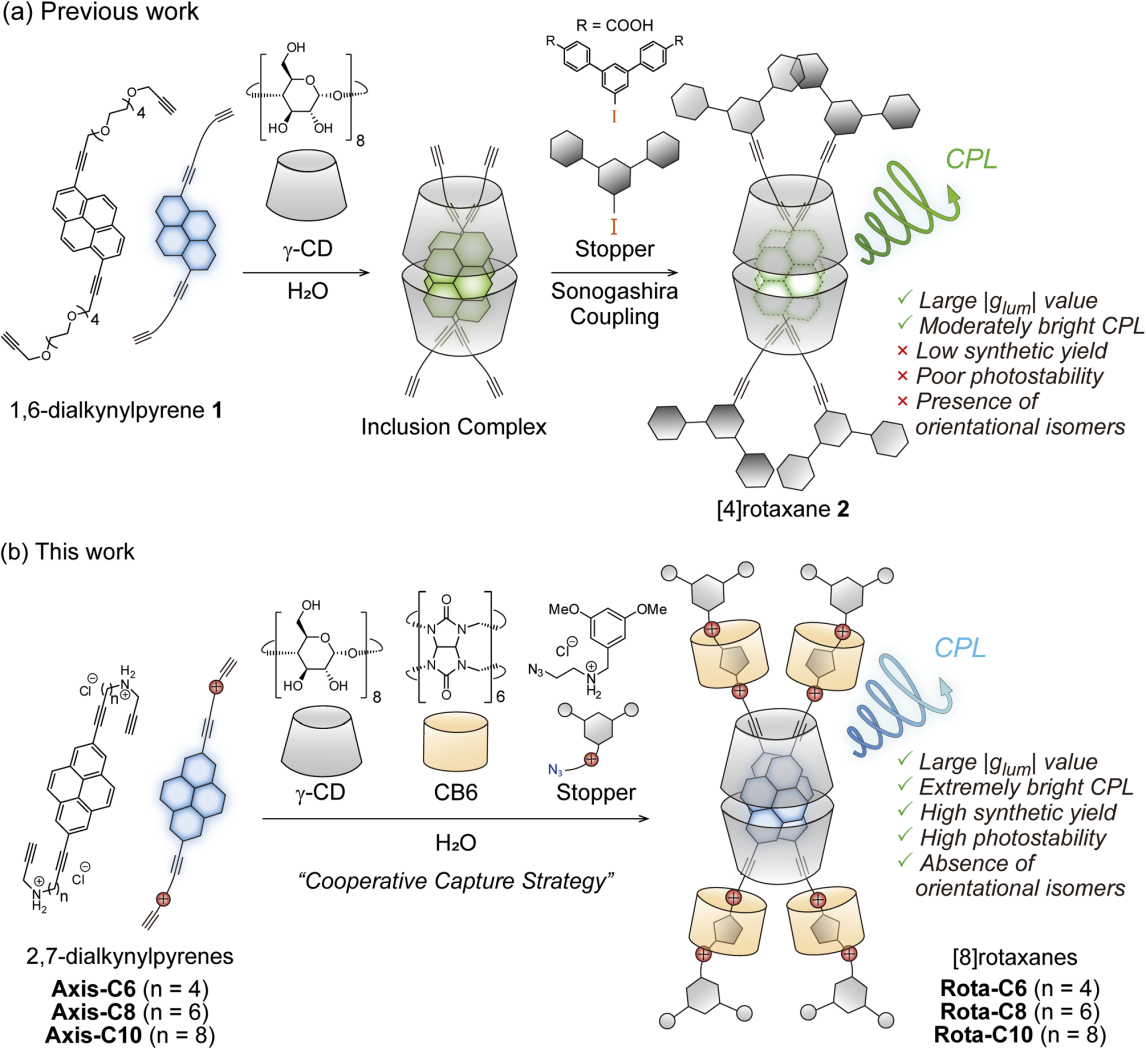
Schematic illustration of rotaxane-type CPL fluorophores. (a) Previous [4]rotaxane-type CPL fluorophore. (b) [8]Rotaxane-type CPL fluorophores in this work.

Rotaxane architecture can realize irreversible encapsulation of fluorophores as opposed to the host–guest strategy mentioned above.^27^ Rotaxane has a mechanically interlocked structure, in which bulky stopper moieties prevent dissociation of guest-like fluorescent axis and host-like ring components.^28,29^ Indeed, rotaxane-type fluorescent dyes containing CD rings are known to have unique photophysical properties.^30–37^ Particularly, we reported that doubly dialkynylpyrene- and dialkynylperylene-threaded [4]rotaxanes having two γ-CDs exhibited strong CPL with large |*g*_lum_| values on the order of 10^−2^ (Fig. 1a).^38,39^ Despite the outstanding CPL properties, their syntheses remain highly challenging. Synthetic yields of these rotaxanes were extremely low (3–5%) due to the entropic disadvantage associated with the sequential assembly of the multiple components. Furthermore, the separation of the isomeric rotaxane originating from the orientation of the two inner 1,6-disubstituted pyrene axes 1 has not been achieved (*vide infra*). More problematically, the alkynylpyrene-threaded [4]rotaxane 2 showed poor stability under photoirradiation (Fig. S1). The axis 1 required long linkers connecting the alkynylpyrene core and the reactive sites against the stopper molecules because CD rings and other stopper sites sterically hinder the capping reactions (Fig. S2). As a result, the γ-CD rings shuttle along the long linkers, exposing the inner fluorophores to external reactive species photochemically generated. Thus, it is essential that a high-yield synthetic method for rotaxanes possessing sufficient photostability must be developed for applying them to materials of optical interest.

In this research, we succeeded in synthesizing doubly 2,7-dialkynylpyrene-threaded [8]rotaxanes made of two 2,7-dialkynylpyrenes having acetylene termini, two γ-CDs, four cucurbit[6]urils (CB6s), and four resorcinol derivatives having azido termini as stopper molecules using a cooperative capture strategy in high yields (Fig. 1b).^40–43^ This synthetic strategy enables efficient rotaxane synthesis because the capping reaction proceeds inside CB6 between the acetylene and the azido termini after the spontaneous assembly of the eight rotaxane components. Here, we report the detailed synthetic procedure, structural analysis, and photophysical properties of the [8]rotaxane-type CPL fluorophores.

## Results and discussions

We designed 2,7-dialkynylpyrene derivatives Axis-C6, Axis-C8, and Axis-C10 as axis components (Fig. 1b). These axes were synthesized from 2,7-dibromopyrene by Sonogashira reaction and Fukuyama amine synthesis, subsequently (Scheme S1). The axes having two ammonium cation moieties are applicable for syntheses of rotaxane-type fluorescence dyes based on a cooperative capture strategy, which we established as a high-yield synthetic method of singly fluorophore-threaded [5]rotaxanes with two CDs and two CB6s.^43^ In this method, the negatively charged CB6 rims attract both ammonium cations of the axis and of the stopper molecule by electrostatic interaction, causing an alkyne–azide cycloaddition in the CB6 cavity without Cu(i) catalyst. Furthermore, CB6s attract CDs through hydrogen-bonding between the carbonyl groups of CB6s and the hydroxy groups of CDs. Owing to this attraction by CB6 against axes, stopper molecules, and CDs, the syntheses of [8]rotaxanes do not require excessively long linkers along which the CD rings shuttle (Fig. S2). The *D*_2h_-symmetrical 2,7-disubstituted pyrene structure no longer produces the isomers in regard to the relative orientations of the inner pyrene pair (*vide supra*, Fig. S3). The absence of orientational isomers would lead to the doubly alkynylpyrene threaded [8]rotaxanes showing sharp absorption bands and thus achieving a high *B*_CPL_ value.

**Fig. 2:**
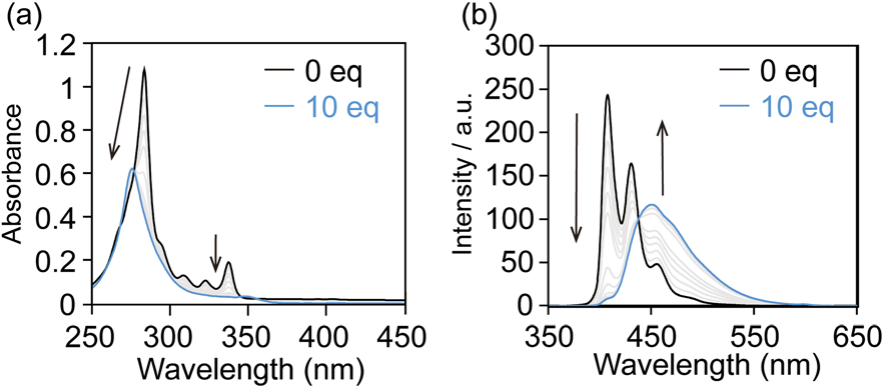
Titration experiments of Axis-C6 with γ-CD. Changes of (a) UV-vis absorption spectra and (b) fluorescence emission spectra of Axis-C6 induced by titration of γ-CD. Conditions: [Axis-C6] = 5.0 × 10^−6^ M, [γ-CD] = 0 to 5.0 × 10^−5^ M, H_2_O with 0.1% HCO_2_H, path length = 10 mm, 25 °C, *λ*_ex_ = 300 nm.

Prior to the rotaxane syntheses, the affinities between the alkynylpyrene axes (Axis-C6, Axis-C8, and Axis-C10) and γ-CD were evaluated because stopper molecules must react with thus generated pseudo-rotaxane complex. When γ-CD was added to an aqueous solution of Axis-C6, the original absorption peak around 290 nm was slightly blue-shifted to 277 nm and weakened as a similar manner of DNA duplex formation (Fig. 2a). In the emission spectra, the shape of the emission band changed significantly. After the addition, initial monomer emission immediately disappeared, and a new broad emission band emerged in the longer wavelength region (Fig. 2b). This new emission band stems from the excimer emission often observed from a 2 : 2 complex consisting of pyrene derivatives and γ-CDs.^24^ The association between Axis-C6 and γ-CD was also monitored by circular dichroism spectroscopy (Fig. S4). The mixture of Axis-C6 and γ-CD showed cotton effects in the region where Axis-C6 absorbs. This finding suggested that γ-CD induced asymmetrical stacking of the alkynylpyrene pair inside. When Axis-C8 or Axis-C10 was used as an axis component, titration experiments reproduced the results for Axis-C6 (Fig. S5 and S6). Considering this information and the cavity size of γ-CD, we concluded that the axes and γ-CD predominantly form 2 : 2 complexes.

**Scheme 1:**
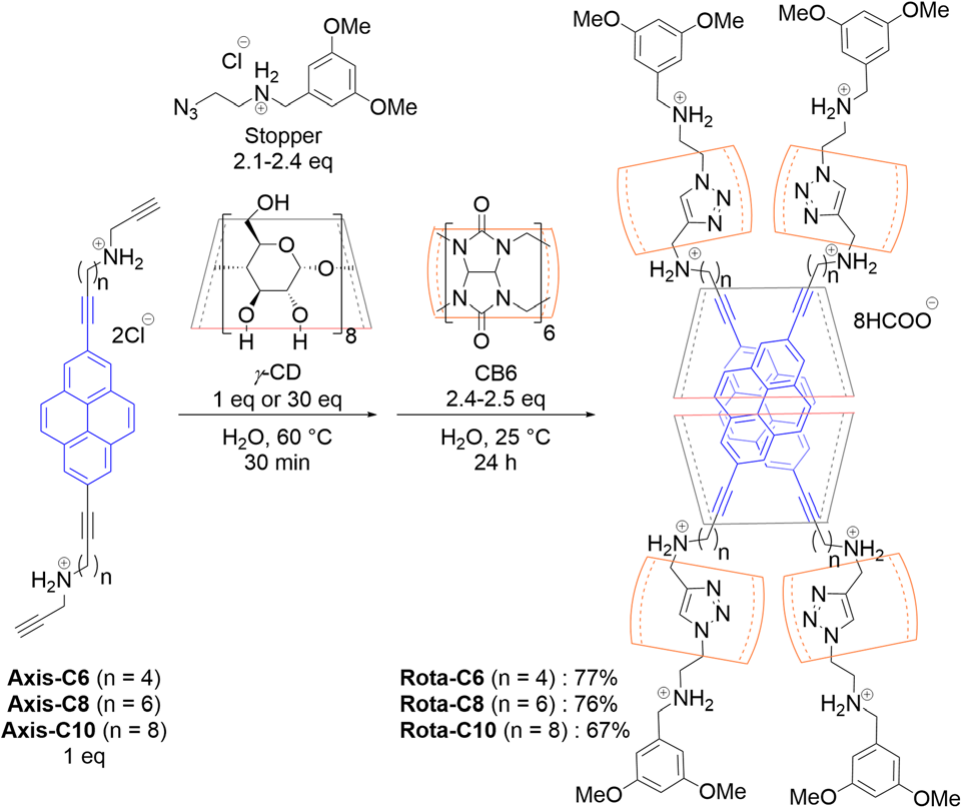
Syntheses of [8]rotaxanes.

A cooperative capture strategy was utilized to realize a high-yield synthesis of a doubly 2,7-dialkynylpyrene-threaded [8]rotaxanes (Scheme 1 and Table 1). An aqueous solution of Axis-C6 or Axis-C8 (4.0 × 10^−3^ M, 1 equiv.), the stopper molecule (2.3–2.4 equiv.), and an excess amount of γ-CD (30 equiv.) was heated at 60 °C for 30 min. After the cooling to 25 °C, CB6 (2.5 equiv.) was added to the solution to initiate the stopping reaction. The reaction was almost completed for 12 hours (Fig. S7 and S8), and Rota-C6 and Rota-C8 were isolated by reverse-phase HPLC as formate salts in high yields of 77% and 76%, respectively (Table 1, Entries 1 and 2). However, when Axis-C10 was used as an axis component, this condition gave not only [8]rotaxane but also [9]rotaxane and [10]rotaxane as side-products containing excess γ-CD rings (Fig. S9). The existence of these side-products meant that the alkyl linkers of Axis-C10 were too long to synthesize only [8]rotaxane. In order to suppress the excessive encapsulation, only 1 equivalent of γ-CD (4.2 × 10^−3^ M) was used for [8]rotaxane synthesis. This refined condition selectively furnished Rota-C10 (isolated in 67%, Table 1, Entry 3, Fig. S9). This synthetic strategy was quite effective in comparison with our previous doubly fluorophore-threaded [4]rotaxanes, whose synthetic yields were 3–5%, accompanied by exceedingly laborious separation.^38,39^

**Table 1:** Synthetic conditions for constructing [8]rotaxanes^a^

Entry	Axis	γ-CD	Yields
1	Axis-C6	30 equiv.	77%
2	Axis-C8	30 equiv.	76%
3	Axis-C10	1 equiv.	67%

a Conditions: [axis] = 4 mM, stopper = 2.1–2.4 equiv., CB6 = 2.4–2.5 equiv., 25 °C, 24 h.

**Fig. 3:**
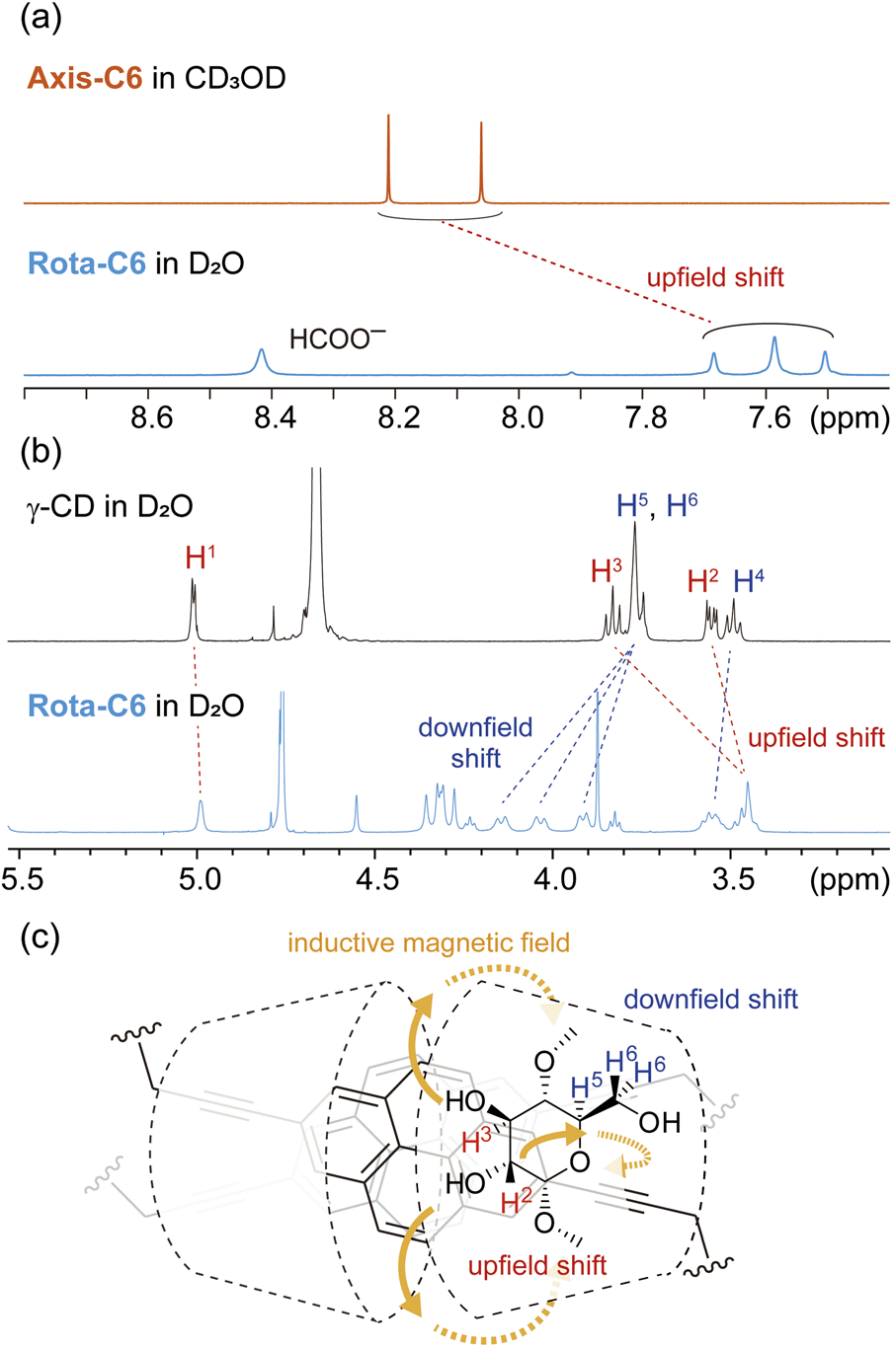
Identification of Rota-C6 by ^1^H NMR experiments. (a) ^1^H NMR spectra of Axis-C6 and Rota-C6. (b) ^1^H NMR spectra of γ-CD and Rota-C6. (c) Schematic illustration of ring current effect of Rota-C6. Conditions: [Axis-C6] = [γ-CD] = 4.0 × 10^−3^ M, [Rota-C6] = 2.0 × 10^−3^ M, 500 MHz, 25 °C, CD_3_OD (Axis-C6), D_2_O (Rota-C6, γ-CD).

The structural arrangement of Rota-C6 was identified by various ^1^H NMR experiments. 2D COSY and NOESY experiments allowed the full assignment of C–H protons in Rota-C6 and confirmed the presence of a single isomer with different orientations against the two outer γ-CDs, that is for head-to-head, head-to-tail, and tail-to-tail (Fig. S10–S14). In the 1D ^1^H NMR spectrum, the pyrene protons of Rota-C6 shifted upfield in D_2_O compared with those of Axis-C6 in CD_3_OD (Fig. 3a). These shifts indicate that the two alkynylpyrene cores are in proximity within the γ-CD cavity.^38^ The signals of γ-CD protons also shifted after the rotaxane formation (Fig. 3b). The C–H proton signals at C2 and C3 (H^2^, H^3^) of γ-CDs shifted upfield, while the signals assigned at C5 and C6 (H^5^, H^6^) shifted downfield. These signal shifts are similar to our previous singly fluorophore-threaded [5]rotaxanes^43^ and are rationalized by magnetic anisotropic effects from the alkynylpyrene core (Fig. 3c). The individual upfield and downfield shifts suggest that two γ-CDs are orientated in a head-to-head manner, in other words, their wider rim sides face each other (Fig. 3c). This head-to-head orientation was also confirmed by the 2D NOESY spectrum (Fig. S13). Strong NOE correlations were observed between all pyrene protons and H^3^ protons placed at the wider rim side of γ-CDs. Furthermore, propargyl proton H^a^ showed NOE with H^6^ protons of γ-CDs, reinforcing narrower rims located far from the alkynylpyrene cores. ^1^H NMR spectra of Rota-C8 and Rota-C10 also showed similar characteristics, and their COSY and NOESY spectra indicated the head-to-head orientations (Fig. S15–S24). The proton signals of the pyrene cores in the 2,7-dialkynylpyrene-based [8]rotaxanes were drastically simpler than those of the previous 1,6-dialkynylpyrene-based [4]rotaxane 2, demonstrating the absence of isomeric rotaxane originating from the orientation of the two inner pyrenes.

**Fig. 4:**
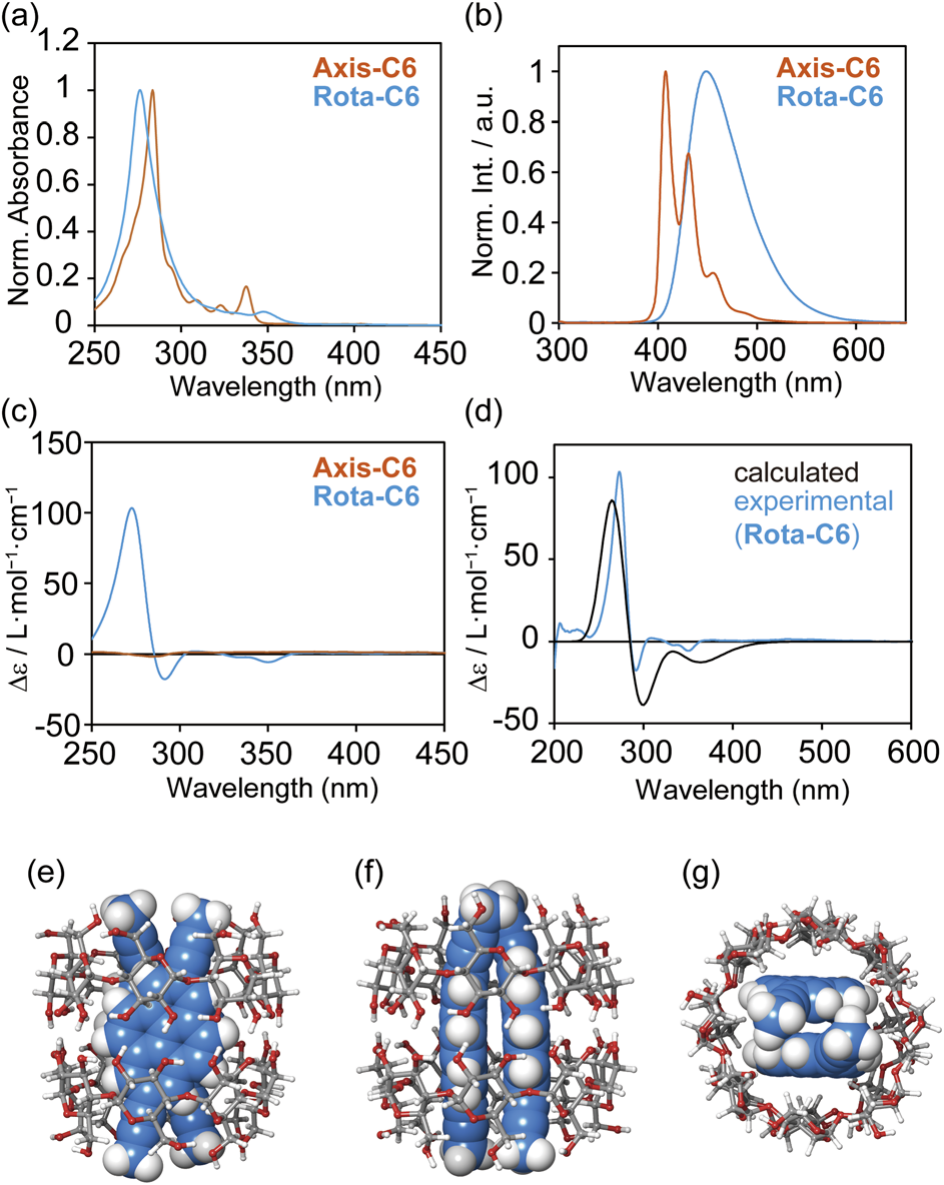
Photophysical and chiroptical properties of Rota-C6. (a) UV-Vis absorption, (b) fluorescence emission, and (c) circular dichroism spectra of Axis-C6 (orange) and Rota-C6 (blue). (d) Circular dichroism spectrum of Rota-C6 (blue) and calculated circular dichroism spectrum of (Py)_2_ ⊂ (γ-CD)_2_ (black). Conditions: [Axis-C6] = 5.0 × 10^−6^ M in H_2_O with 0.1% HCO_2_H, [Rota-C6] = 5.0 × 10^−6^ M in H_2_O, path length = 10 mm, 25 °C, *λ*_ex_ = 300 nm (Axis-C6), 280 nm (Rota-C6). (e–g), optimized structure obtained from DFT calculation of model compound of (Py)_2_ ⊂ (γ-CD)_2_ at (e) front view, (f) side view, and (g) top view. The 2,7-di(propyn-1-yl)pyrene (Py) is virtual and a simple analogue of Axis-C6. Conditions: B3LYP/6-31G, under vacuum.

The desirable photophysical properties of [8]rotaxanes were revealed by the absorption and emission spectra in comparison with those of the previous [4]rotaxane 2 and the axes themselves in H_2_O (Fig. 4a, b, S26–S33 and Table 2). The Rota-C6 indeed showed a sharp absorption band at 277 nm and a large molar absorption coefficient of *ε* = 2.5 × 10^5^ M^−1^ cm^−1^, while in the case of 2, *ε* = 4.3 × 10^4^ M^−1^ cm^−1^ at 370 nm.^38^ The absorption band of Rota-C6 slightly blue-shifted compared to that of Axis-C6 (Fig. 4a). This type of blue shift was not observed in the singly alkynylpyrene-threaded [5]rotaxane and may be attributed to the stacking of a pair of pyrenes. Emission spectra indicated that Axis-C6 displayed sharp monomer emission, whereas Rota-C6 exhibited only broad excimer emission (Fig. 4b). The absorption and excimer emission bands of Rota-C6 were maintained in the concentration range from 7.5 × 10^−5^ to 8.0 × 10^−8^ M (Fig. S28 and S29). This concentration independences prove that the inner alkynylpyrene pair never aggregates or dissociates in either concentrated or diluted solutions. The absolute *Φ*_lum_ of Rota-C6 in H_2_O was measured as 0.47 (Table 2). This strong excimer emission accounts for the fact that the γ-CD rings suppress molecular vibration and intermolecular aggregation of the inner alkynylpyrene pair. Rota-C8 and Rota-C10 also showed strong but slightly weak emission (*Φ*_lum_ = 0.45 and 0.40). This difference suggests that the longer alkyl linkers somewhat allow the shuttling of the γ-CD rings, resulting in the nonradiative transitions.

**Table 2:** Photophysical properties of axes and [8]rotaxanes

Fluorophore	Aqueous solution						Solid state
	*λ*_abs_ [nm]	*ε* [ ×10^4^ M^−1^ cm^−1^]	*λ*_em_ [nm]	*Φ*_lum_	*g*_lum_ [ ×10^−3^]	*B*_CPL_ [M^−1^ cm^−1^]	*Φ*_lum_
Complex of Axis-C10 and γ-CD a^,^^b^	277	13^c^	453	0.41	−7.4	197	—
Rota-C6^a^	277	25^d^	448	0.47	−11	646	0.29
Rota-C8^a^	277	23^d^	452	0.45	−11	569	—
Rota-C10^a^	277	22^d^	454	0.40	−19	836	—
[4]Rotaxane 2^e^	370	4.3	528	0.37	−15	119	—

a Conditions: H_2_O, 25 °C, *λ*_ex_ = 280 nm.

b Conditions: [Axis-C10] = [γ-CD] = 2.0 × 10^−5^ M.

c This value was calculated based on the concentration of the 2 : 2 complex ([2 : 2 complex] = 1.0 × 10^−5^ M).

d Because the absorption bands of stopper and triazole moieties overlap that of 2,7-dialkynylpyrene cores, the *ε* values of 2,7-dialkynylpyrene cores were calculated by subtracting the molar absorption coefficient of stopper and triazole moieties (8.0 × 10^3^ M^−1^ cm^−1^, Fig. S34).

e These values were reported by our previous literature.^38^ Conditions: H_2_O containing NH_3_ (pH 9.5), 25 °C, *λ*_ex_ = 370 nm.

The chiroptical properties of [8]rotaxanes were unveiled by circular dichroism spectroscopy (Fig. 4c). Although the complex of Axis-C6 and γ-CD showed weak Cotton effects (Fig. S35a), Rota-C6 exhibited strong ones (Fig. 4c). Moreover, Rota-C8 and Rota-C10 showed similar shapes of circular dichroism spectra (Fig. S26c and S27c). These chiroptical features of [8]rotaxanes are definitely attributed to the asymmetrically stacked two alkynylpyrene cores tightly restrained by γ-CDs, even if the alkyl-chains became longer. In the [8]rotaxanes, the two negative and one positive circular dichroism signals were observed in the longer and shorter wavelength regions around 360–275 nm, respectively. Judging from the exciton coupling theory,^44^ we could predict that the asymmetrical stacking of the alkynylpyrene pair is biased with a left-handed chirality. This arrangement coincides with the stable conformation optimized by quantum chemical calculations (Fig. 4e–g). The conformational search was performed for the 2 : 2 inclusion complex of 2,7-di(propyn-1-yl)pyrene (Py), the simple model of [8]rotaxanes and γ-CD on the basis of MacroModel-based Monte Carlo simulation. The obtained left-handed stable structure was then further optimized by density functional theory (DFT) calculation. Using this most stable conformation, a time-dependent DFT (TD-DFT) calculation was performed to predict the transition energy. From the TD-DFT calculation, the simulated circular dichroism spectrum showed good agreement with the experimentally observed one of Rota-C6 (Fig. 4d), confirming the left-handed conformation of the alkynylpyrene pair in [8]rotaxanes.

**Fig. 5:**
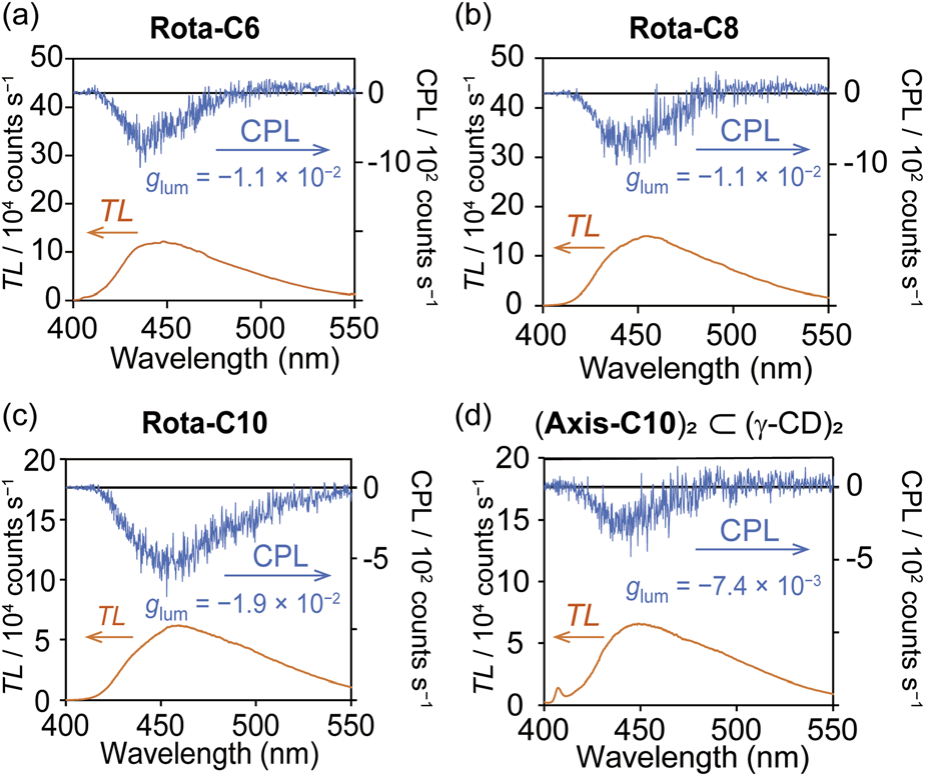
CPL characteristics of [8]rotaxanes. CPL spectra of (a) Rota-C6, (b) Rota-C8, (c) Rota-C10, and (d) inclusion complex of (Axis-C10)_2_ ⊂ (γ-CD)_2_. Conditions: [Rota-C6] = [Rota-C8] = [Rota-C10] = 5.0 × 10^−6^ M, [Axis-C10] = [γ-CD] = 2.0 × 10^−5^ M, H_2_O, path length = 10 mm, 25 °C, *λ*_ex_ = 300–380 nm.

In the CPL spectrum, [8]rotaxanes exhibited a negative CPL band (Fig. 5a–c), and the |*g*_lum_| values were as large as that of the previous [4]rotaxane 2 (Table 2 and Fig. S36).^38^ The minus sign of the CPL is known to be observed from left-handed twisted excimer,^45^ which means that the 2,7-dialkynylpyrene pair predominantly forms left-handed stacking in both the ground and excited states. Combined with the high *ε* and *Φ*_lum_ values of the [8]rotaxanes, the *B*_CPL_ values of 569–836 were as bright as the highest ones in reported organic CPL emitters containing mechanically interlocked molecules (Table S1).^8,46,47^ This *B*_CPL_ value was far higher than those of the inclusion complex between the Axis-C10 and γ-CD (*B*_CPL_ = 197, Fig. 5d) and the previous [4]rotaxane 2 (*B*_CPL_ = 119).

**Fig. 6:**
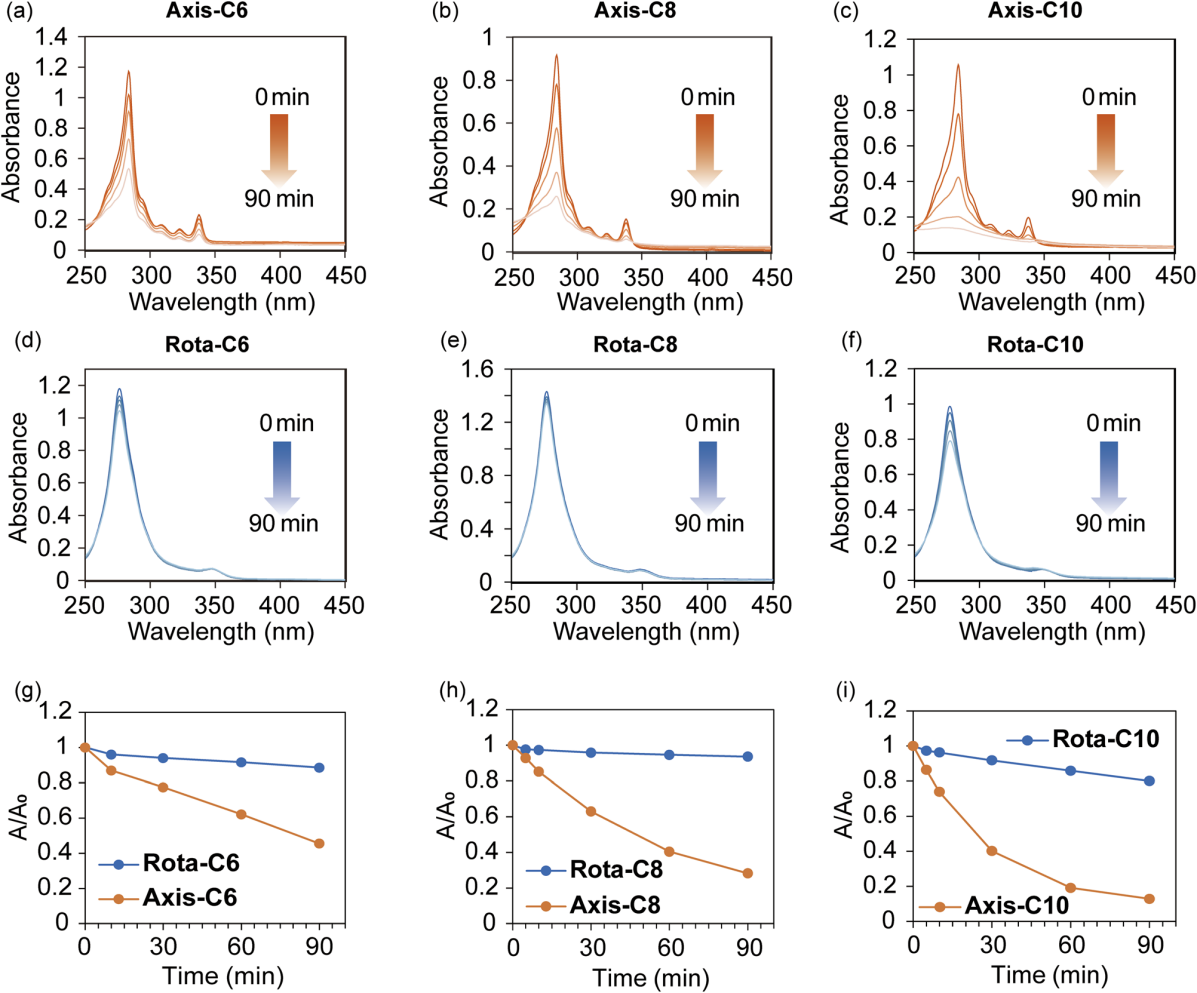
Photostability of [8]rotaxanes. Changes of UV-vis absorption spectra of (a) Axis-C6, (b) Axis-C8, (c) Axis-C10, (d) Rota-C6, (e) Rota-C8, and (f) Rota-C10 during irradiation of high-pressure mercury lamp. Conditions: [Axis-C6] = [Axis-C8] = [Axis-C10] = 5.0 × 10^−6^ M in H_2_O with 0.1% HCO_2_H, [Rota-C6] = [Rota-C8] = [Rota-C10] = 5.0 × 10^−6^ M in H_2_O, path length = 10 mm, 25 °C. Light source: 250 W high-pressure mercury lamp. (g–i) Absorption changes of (g) Axis-C6 and Rota-C6, (h) Axis-C8 and Rota-C8, and (i) Axis-C10 and Rota-C10 over the time.

Focusing on the application of the [8]rotaxanes, the photostability was evaluated by absorption and emission changes under continuous photoirradiation (Fig. 6 and S40). Aqueous solutions of the axes and the [8]rotaxanes were irradiated with a high-pressure mercury lamp, and their absorption and emission changes were monitored. The absorption and emission bands of the axes rapidly disappeared due to the photodegradation (Fig. 6a–c and S40a–c), on the contrary, the optical properties of the [8]rotaxanes were almost maintained during the photoirradiation (Fig. 6d–f and S40d–f). In addition, the CD and CPL properties of Rota-C10 were almost maintained after the photostability experiment (Fig. S41). The photostability of the [8]rotaxanes was significantly higher than that of the previous pyrene-based [4]rotaxane 2, suggesting that the successively connected γ-CD and CB6 rings tightly protect inner fluorophores without a break from reactive oxygen species generated by photoirradiation. Heat resistance of the [8]rotaxanes was also evaluated on the basis of fluorescence emission measurements under variable temperatures (Fig. S42). The intensity of the excimer emission of [8]rotaxanes decreased at high temperatures because non-radiative processes were promoted. However, [8]rotaxanes showed no monomer emission, in contrast to the complex of the axes and γ-CD. Furthermore, the chiroptical properties of [8]rotaxanes were reproduced after heating at 80 °C and cooling to 25 °C (Fig. S43). At a high temperature of 50 °C, Rota-C10 exhibited a comparable CPL to that measured at 25 °C (Fig. S44). The irreversibly interlocked structure of rotaxanes proved to be effective for maintaining remarkable CPL activity against the harsh external stimuli.

## Conclusion

In conclusion, we succeeded in developing doubly 2,7-dialkynylpyrene-threaded [8]rotaxanes as new purely organic CPL emitters. The high-yield syntheses were achieved utilizing a cooperative capture strategy, proving this method is an effective method not only for the singly fluorophore-threaded rotaxanes but also for the doubly fluorophore-threaded ones. Photophysical measurements of the [8]rotaxanes elucidated remarkably high *B*_CPL_ values compared with organic CPL emitters so far reported. Chiroptical analyses and quantum chemical calculations suggested left-handed stacking of the inner alkynylpyrene pair. Since this asymmetry is based on the chirality of γ-CD, the opposite chiroptical property would be realized by using γ-l-cyclodextrin, an enantiomer of γ-CD.^48^ The stability analyses revealed the robust resistance of [8]rotaxanes to both photo- and thermal-stimuli. The present study demonstrated that this [8]rotaxane approach was a promising strategy for creating extremely bright organic CPL emitters from various achiral organic fluorophores. A new project to develop NIR emissive [8]rotaxane utilizing this strategy is proceeding now.

## Author contributions

Y. O. conceived and directed the project. K. Ni. and A. K. performed most experiments under the supervision of Y. O., J. C., and M. In. J. Y. and M. K. performed quantum yield measurement under the supervision of M. Iw. J. Y. and M. K. performed CPL measurement under the supervision of M. Iw. and K. No. K. Ni., Y. O., and M. In. wrote the paper. All authors discussed the results and commented on the manuscript.

## Conflicts of interest

There are no conflicts to declare.

## Supplementary Material

SC-016-D5SC06304C-s001

## Data Availability

All data relating to materials and methods, experimental procedures, mechanistic studies, characterization data for all new compounds (^1^H NMR, ^13^C NMR, IR and HRMS), and additional computational results are available in the supplementary information (SI). Supplementary information: detailed synthetic procedures, chemical characterization, and NMR spectra (pdf). See DOI: https://doi.org/10.1039/d5sc06304c.
