# Supplementary material for: High-yield syntheses of doubly 2,7-dialkynylpyrene-threaded photostable [8]rotaxanes exhibiting extremely bright circularly polarized luminescence
Source: Chem Sci. 2025 Sep 26;16(43):20414–21. doi: 10.1039/d5sc06304c (PMC12495304; doi:10.1039/d5sc06304c)

Supplementary Information (SI) for Chemical Science.
This journal is © The Royal Society of Chemistry 2025

Supporting Information

High-yield syntheses of doubly 2,7-dialkynylpyrene-threaded photostable [8]rotaxanes exhibiting extremely bright circularly polarized luminescence

Kohei Nishioki,[a] Asuka Kimura,[a] Yuki Ohishi,[a]* Juri Yamashita,[b] Masahiro Kitamoto,[b] Munetaka Iwamura,[b] Koichi Nozaki,[b] Junya Chiba,[a] and Masahiko Inouye[a]

[a] Graduate School of Medicine and Pharmaceutical Sciences, University of Toyama, Toyama 930-0194, Japan

[b] Graduate School of Science and Engineering, University of Toyama, Toyama 930-8555, Japan

Table of Contents

1. Supplementary data S2

2. Experimental section S27

3. References S38

4. Calculated energies and geometric coordinates S40

5. ^{1}H NMR, ^{13}C NMR, and ESI-MS spectra of new compounds S61

1. Supplementary data

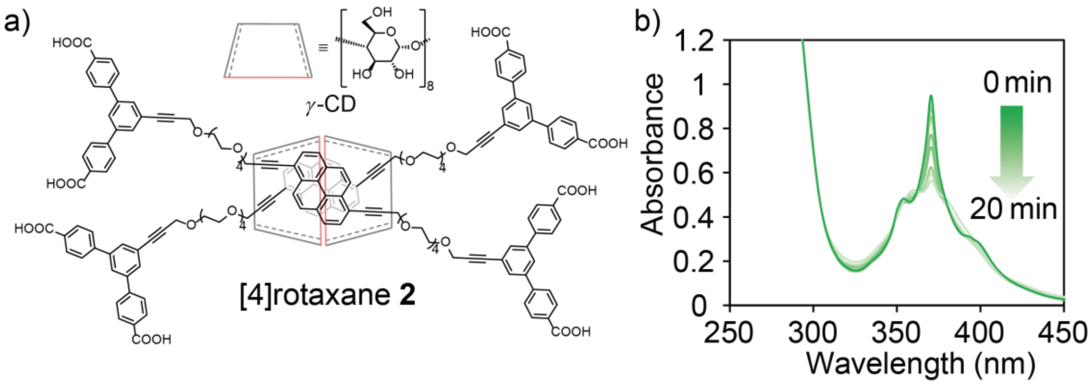

Figure S1. Photostability of [4]rotaxane **2**. (a) Chemical structure of previously reported [4]rotaxane **2**. (b) Changes of UV-Vis absorption spectra of [4]rotaxane **2** during irradiation of high-pressure mercury lamp. Conditions: [**2**] = 2.2 × 10^{-5} M, H_2O containing NH_3 (pH 9.5), path length = 10 mm, 25 °C. Light source: 150 W high-pressure mercury lamp.

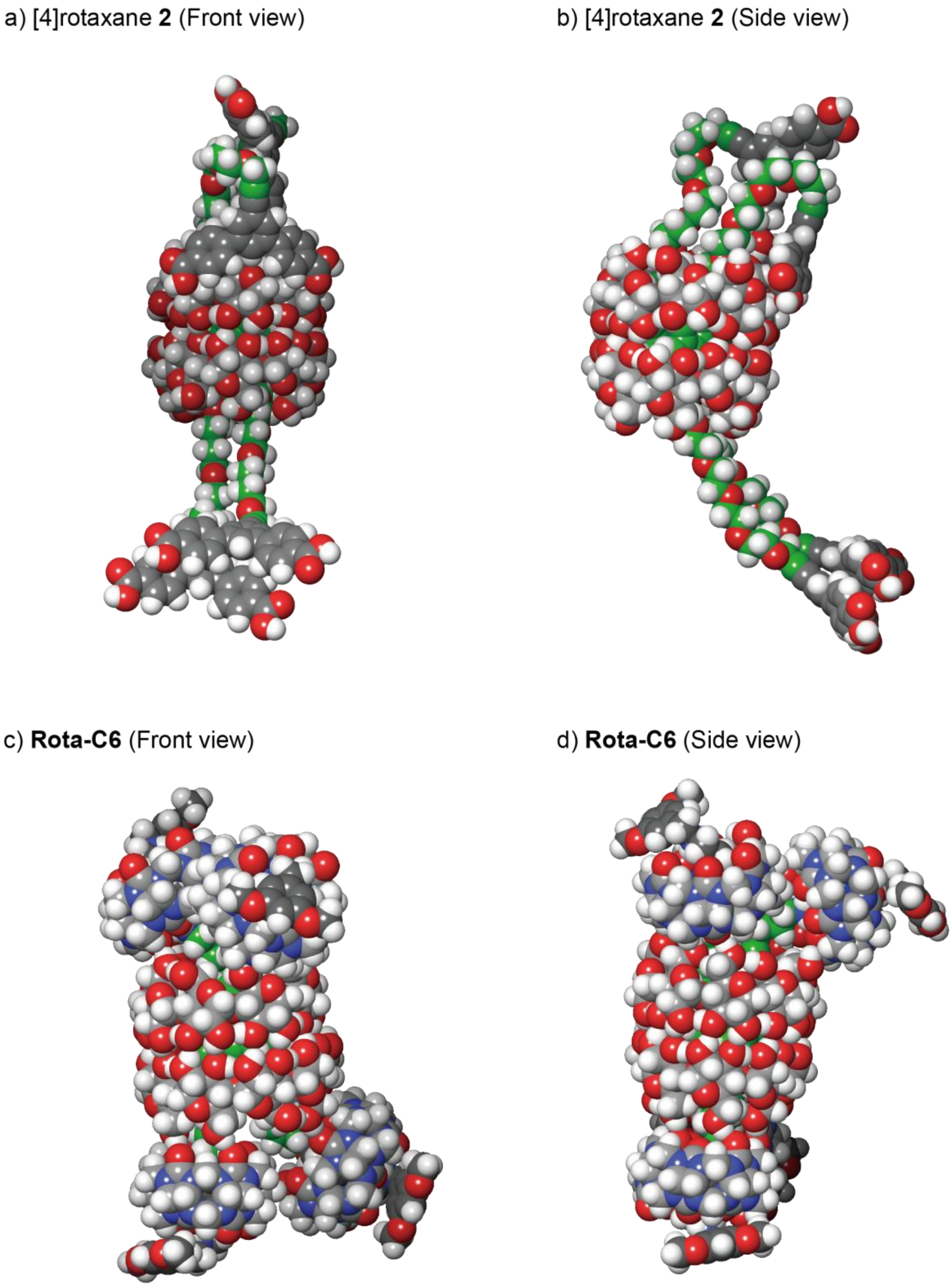

Figure S2. (a, b) CPK models of previously reported [4]rotaxane **2** and (c, d) **Rota-C6** developed in this work. The models were optimized by MacroModel-based Monte Carlo simulation. Conditions: OPLS4, water, 3000 conformations.

Synthesis of Axis-C6

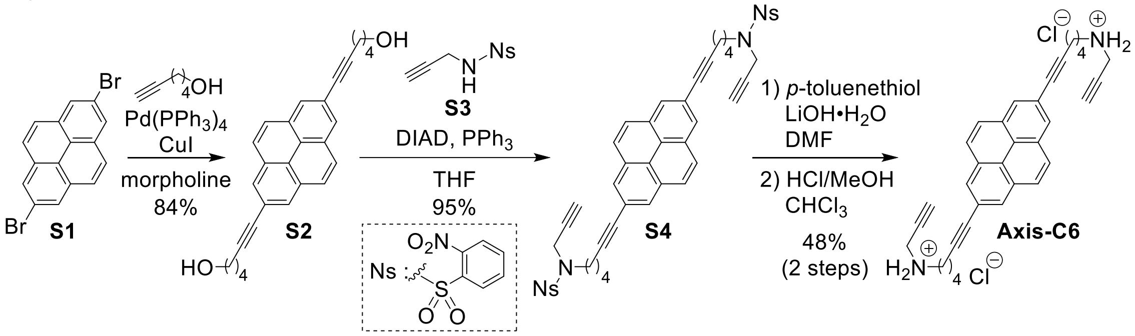

Synthesis of Axis-C8

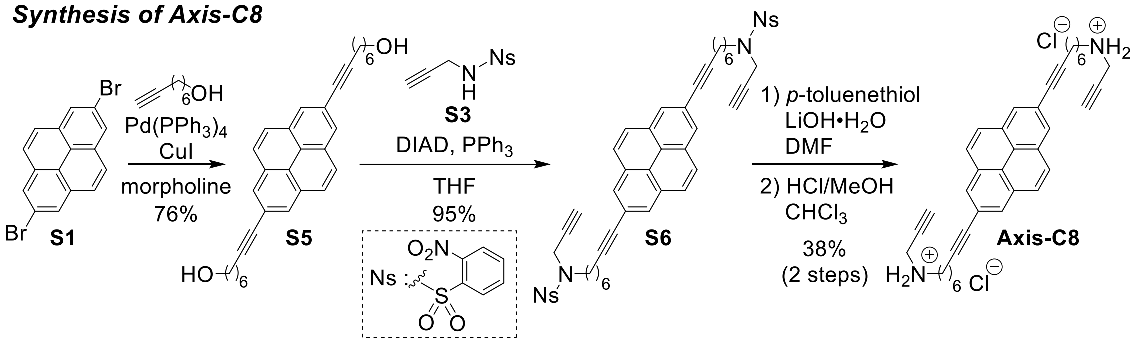

Synthesis of Axis-C10

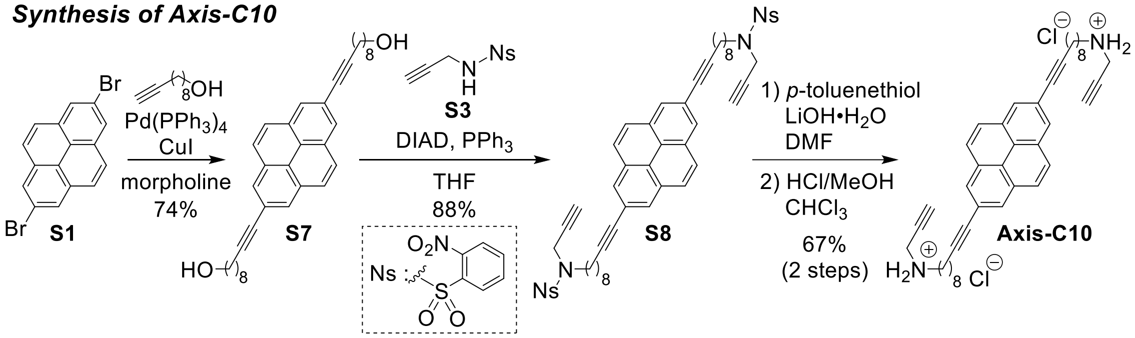

Scheme S1. Syntheses of axes. DIAD: Diisopropyl azodicarboxylate.

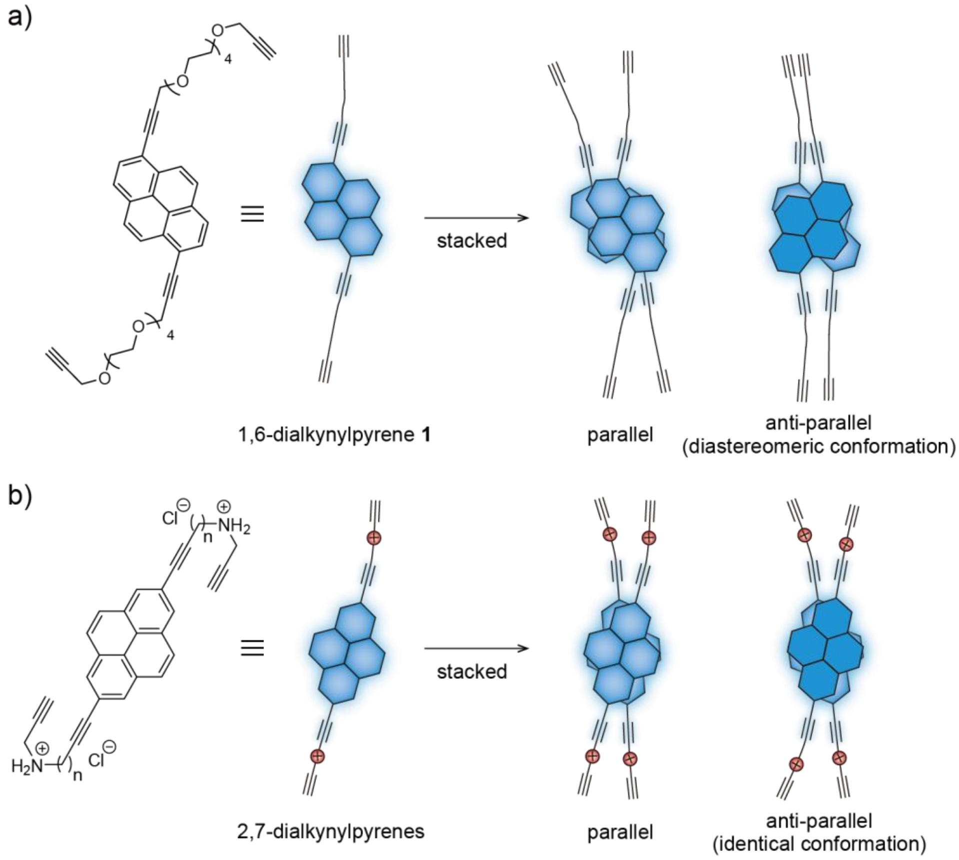

Figure S3. Schematic illustration of conformational isomers derived from stacking manner of two pyrenes. (a) Previously reported 1,6-dialkynylpyrene **1**. (b) 2,7-dialkynylpyrenes developed in this work.

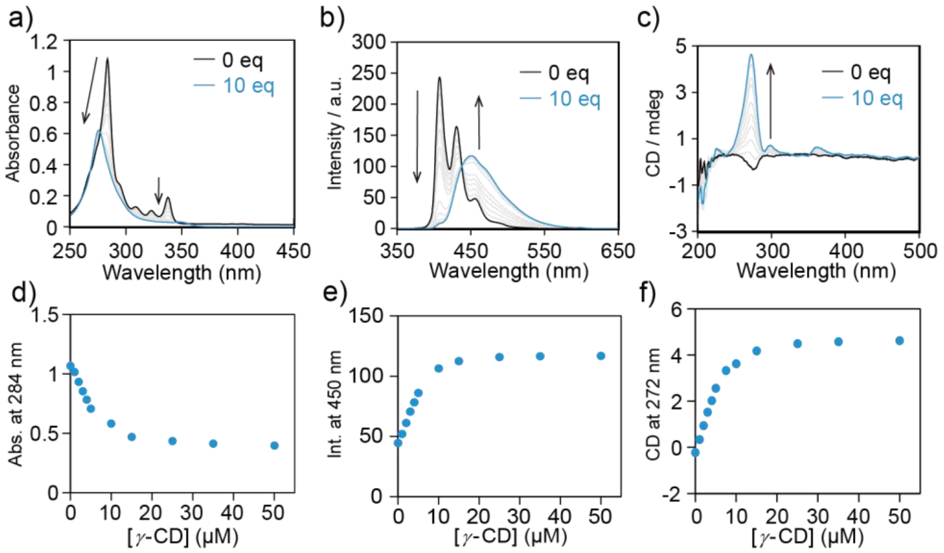

Figure S4. Titration experiments of **Axis-C6** and γ-CD. (a) Absorption spectra. (b) Emission spectra. (c) Circular dichroism spectra. (d–f) Signal changes induced by titration of γ-CD. Conditions: [**Axis-C6**] = 5.0×10^{-6} M, [γ-CD] = 0 to 5.0×10^{-5} M, H_2O with 0.1% HCO_2H, path length = 10 mm, λ_{ex} = 300 nm, 25 °C.

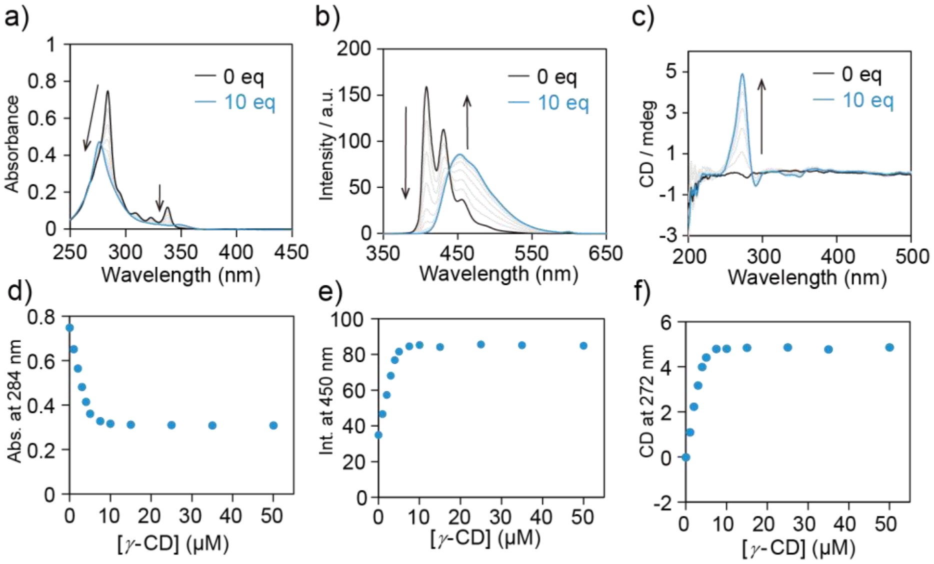

Figure S5. Titration experiments of **Axis-C8** and γ-CD. (a) Absorption spectra. (b) Emission spectra. (c) Circular dichroism spectra. (d–f) Signal changes induced by titration of γ-CD. Conditions: [**Axis-C8**] = 5.0×10^{-6} M, [γ-CD] = 0 to 5.0×10^{-5} M, H_2O with 0.1% HCO_2H, path length = 10 mm, λ_{ex} = 300 nm, 25 °C.

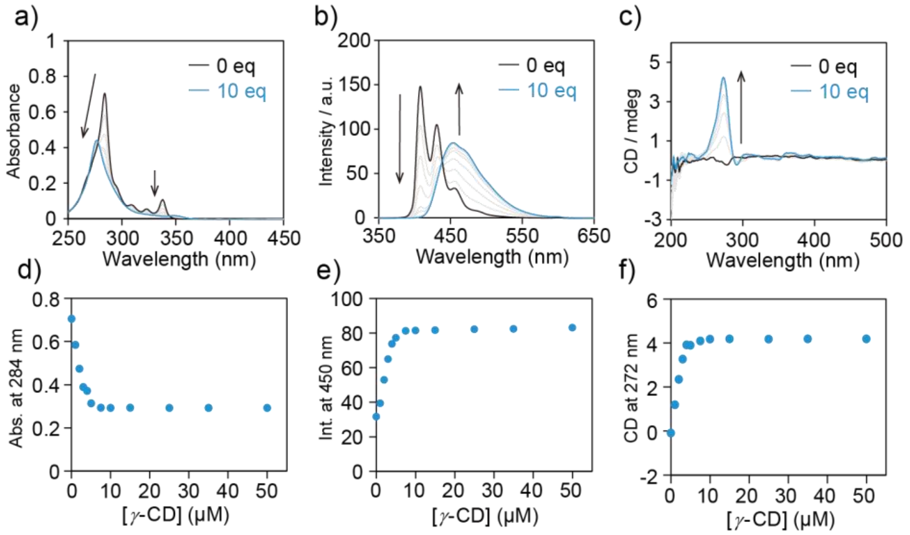

Figure S6. Titration experiments of **Axis-C10** and γ-CD. (a) Absorption spectra. (b) Emission spectra. (c) Circular dichroism spectra. (d–f) Signal changes induced by titration of γ-CD. Conditions: [**Axis-C10**] = 5.0×10^{-6} M, [γ-CD] = 0 to 5.0×10^{-5} M, H_2O with 0.1% HCO_2H, path length = 10 mm, λ_{ex} = 300 nm, 25 °C.

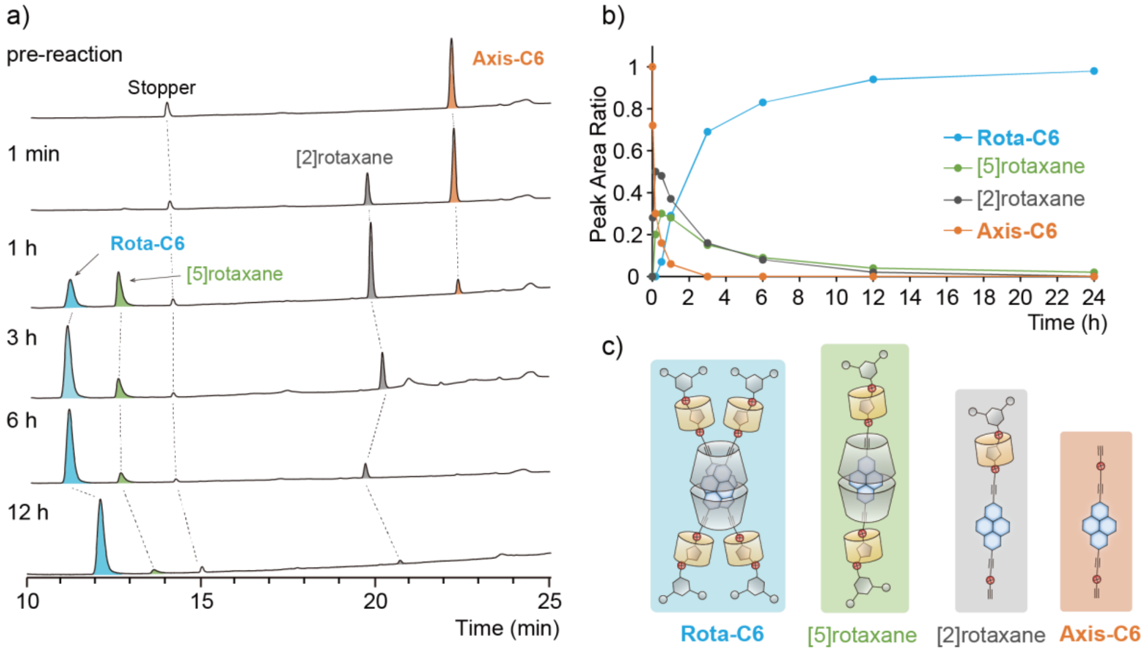

Figure S7. Tracking the process for **Rota-C6** formation by a HPLC/MS method. (a) Time-dependent HPLC/MS analysis of the reaction mixture for synthesizing **Rota-C6**. HPLC conditions: MeCN/H_2O with 0.1% HCO_2H, 0-64% in 35 min and thus 80%, 40 °C, 254 nm (detection). (b) Changing the peak area ratio of the HPLC chart during the reaction. (c) Schematic illustration of the structure of the side-products.

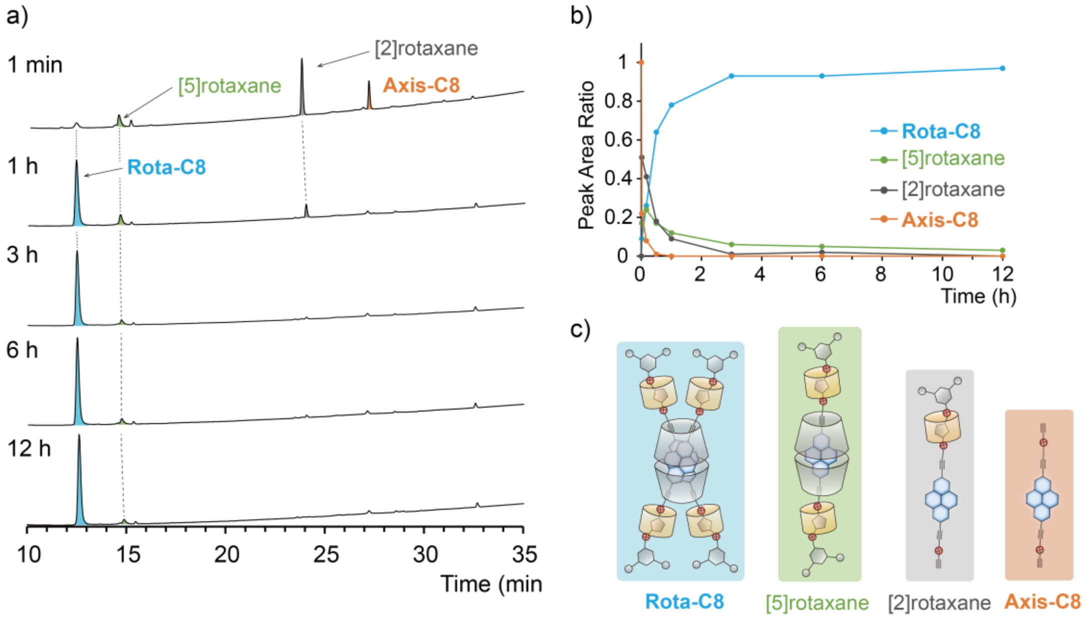

Figure S8. Tracking the process for **Rota-C8** formation by a HPLC/MS method. (a) Time-dependent HPLC/MS analysis of the reaction mixture for synthesizing **Rota-C8**. HPLC conditions: MeCN/H_2O with 0.1% HCO_2H, 0-64% in 35 min and thus 80%, 40 °C, 254 nm (detection). (b) Changing the peak area ratio of the HPLC chart during the reaction. (c) Schematic illustration of the structure of the side-products.

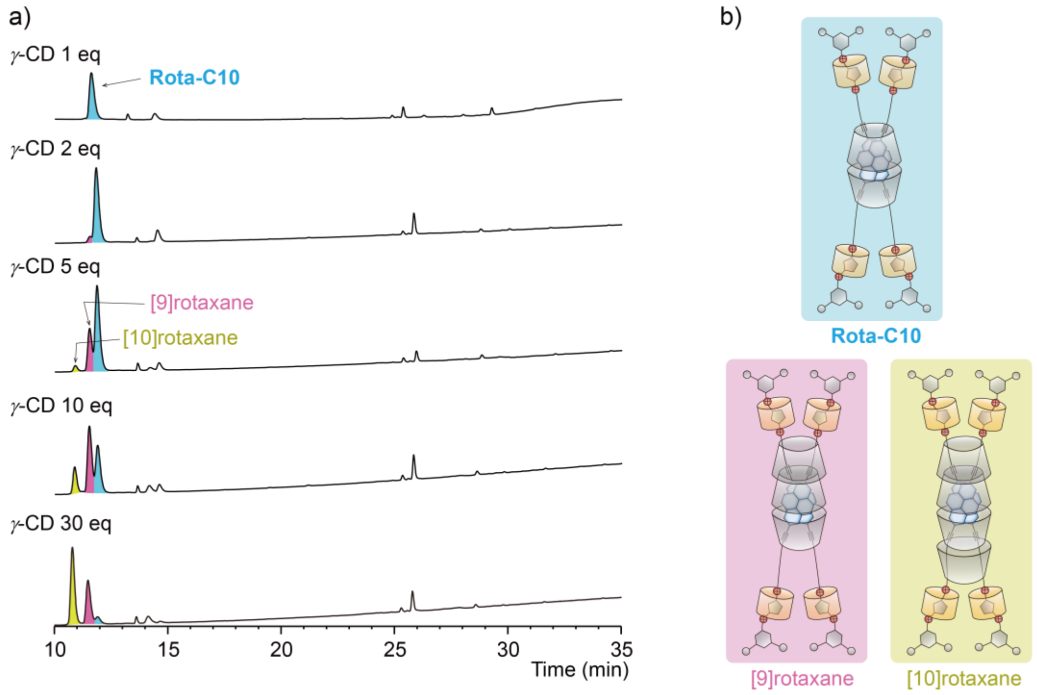

Figure S9. HPLC/MS analysis of the crude product for synthesizing **Rota-C10** using different amounts of γ-CD. (a) HPLC/MS analysis of the crude product for synthesizing **Rota-C10** using different amounts of γ-CD (1 eq to 30 eq). HPLC conditions: MeCN/H_2O with 0.1% HCO_2H, 0-64% in 35 min and thus 80%, 40 °C, 254 nm (detection). (b) Schematic illustration of the structure of the side-products.

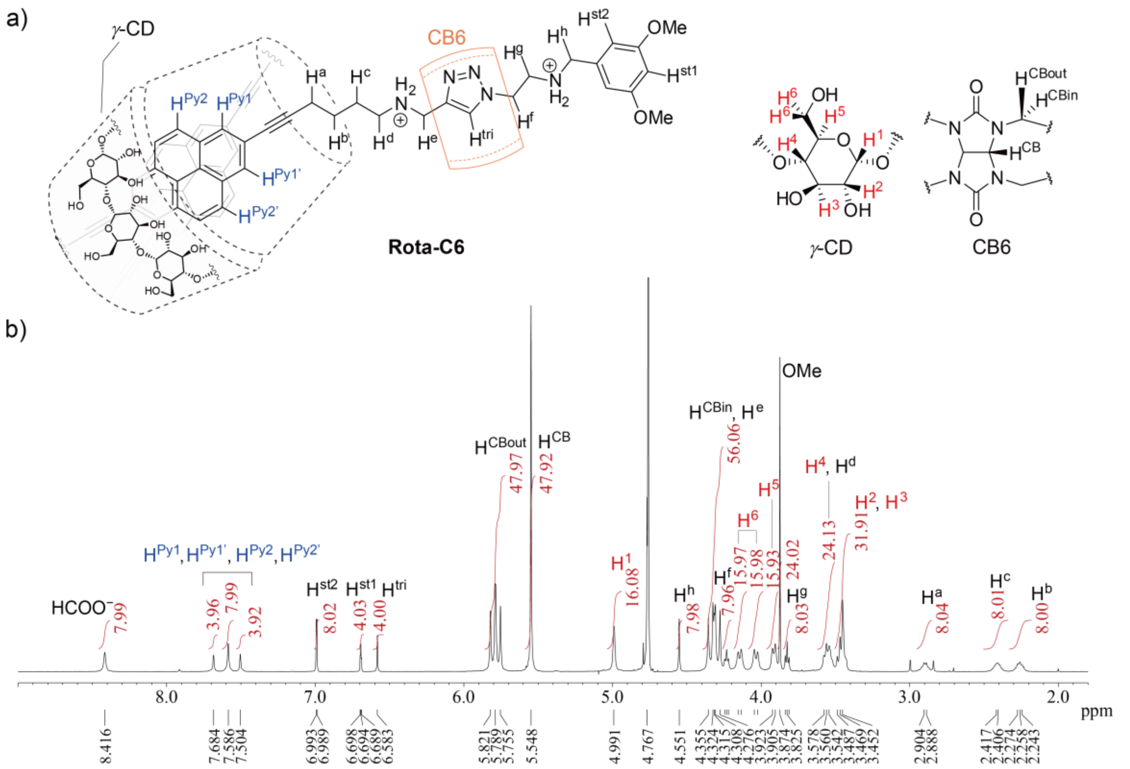

Figure S10. ^{1}H NMR signal assignment of **Rota-C6**. (a) Chemical structures of components of **Rota-C6**. (b) ^{1}H NMR spectrum of **Rota-C6**. Conditions: 2.0 × 10^{-3} M, D$_2$O, 25 °C, 500 MHz. The H^{Py1} and H$^{Py1'}$ are not equivalent due to the influence of γ-CD rings and the other alkynylpyrene axis (see Figure S25). Similarly, the H^{Py2} and H$^{Py2'}$ are also not equivalent.

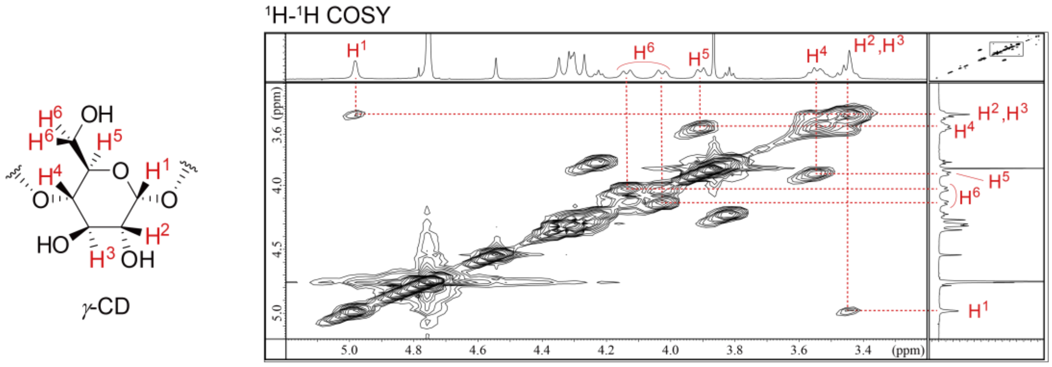

Figure S11. Assignment of ^{1}H NMR signals of γ-CD-ring protons of **Rota-C6**. Chemical structure of γ-CD and ^{1}H-^{1}H COSY NMR spectrum of **Rota-C6**. Conditions: 2.0 × 10^{-3} M, D$_2$O, 25 °C, 500 MHz.

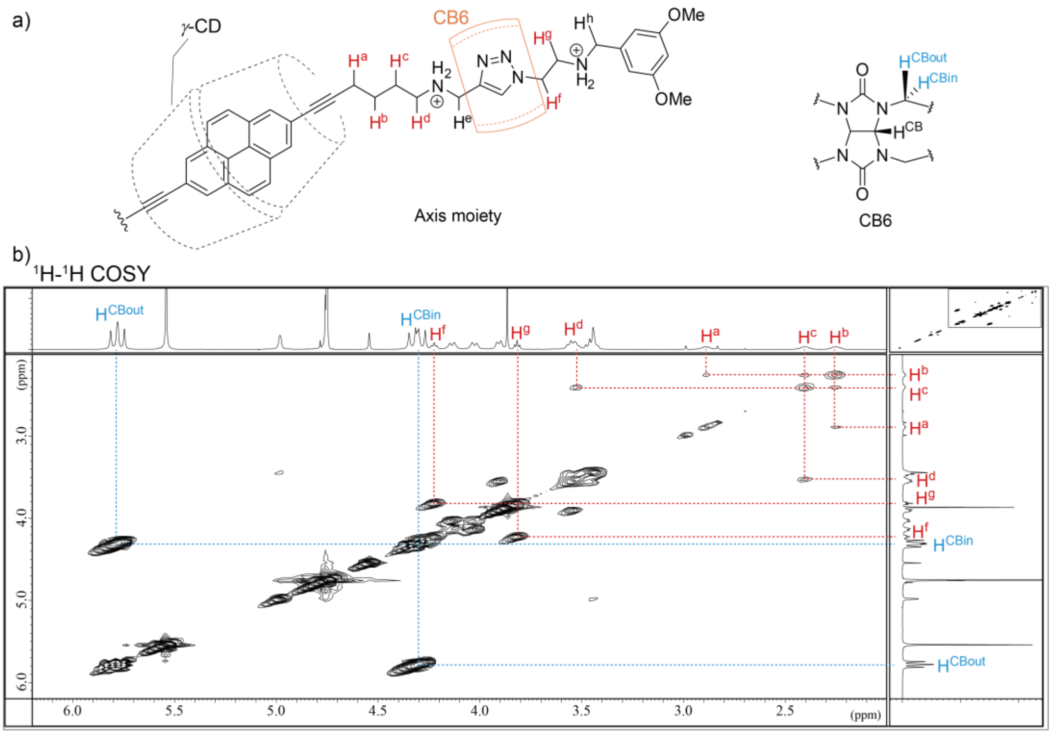

Figure S12. Assignment of ^{1}H NMR signals of methylene-linker and CB6-ring protons of **Rota-C6**. (a) Chemical structures of axis moiety of **Rota-C6** and CB6. (b) ^{1}H-^{1}H COSY NMR spectrum of **Rota-C6**. Conditions: 2.0 × 10^{-3} M, D_2O, 25 °C, 500 MHz.

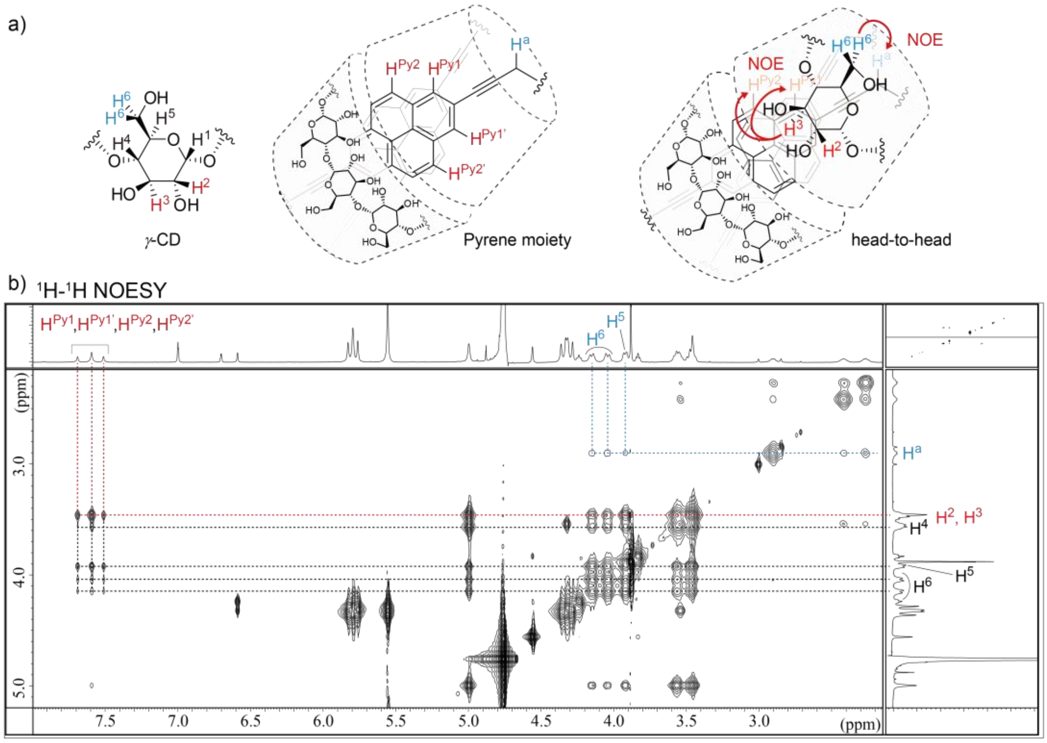

Figure S13. Assignment of head-to-head orientation of **Rota-C6**. (a) Chemical structures of components of **Rota-C6**. (b) ^{1}H-^{1}H NOESY NMR spectrum of **Rota-C6**. Correlation between axis and γ-CD were observed. Conditions: 2.0 × 10^{-3} M, D_2O, 25 °C, 500 MHz.

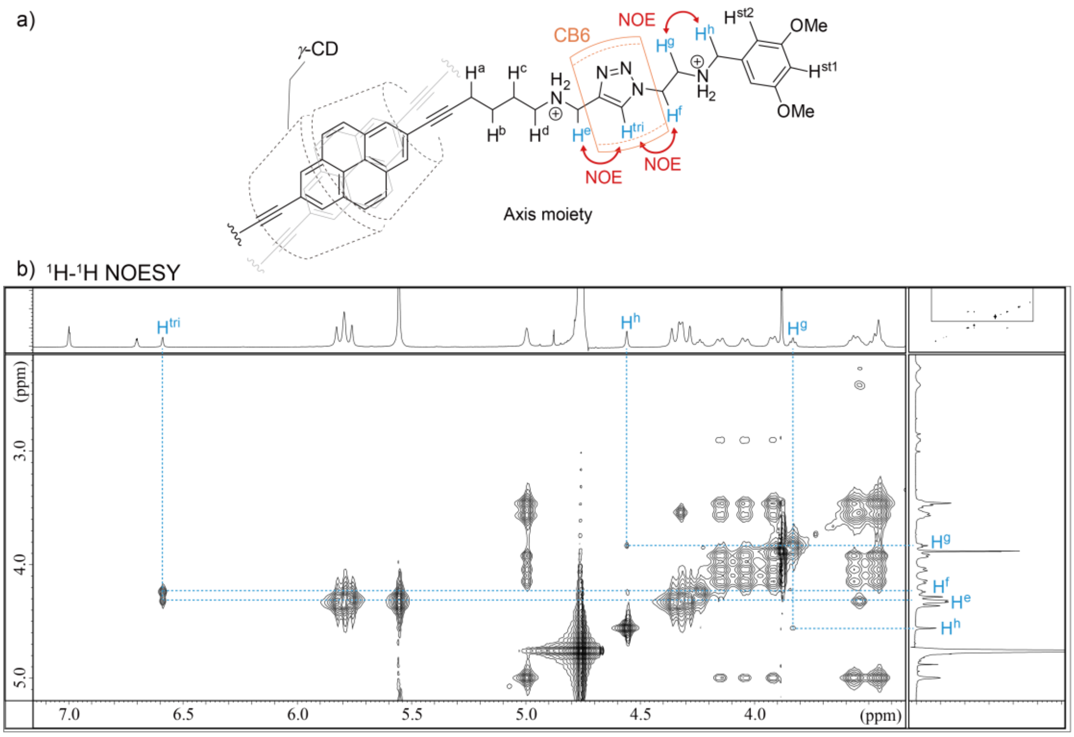

Figure S14. Assignment of ^{1}H NMR signals of methylene-linker and stopper protons of **Rota-C6**. (a) Chemical structure of components of **Rota-C6**. (b) ^{1}H-^{1}H NOESY NMR spectrum of **Rota-C6**. Conditions: 2.0 × 10^{-3} M, D_2O, 25 °C, 500 MHz.

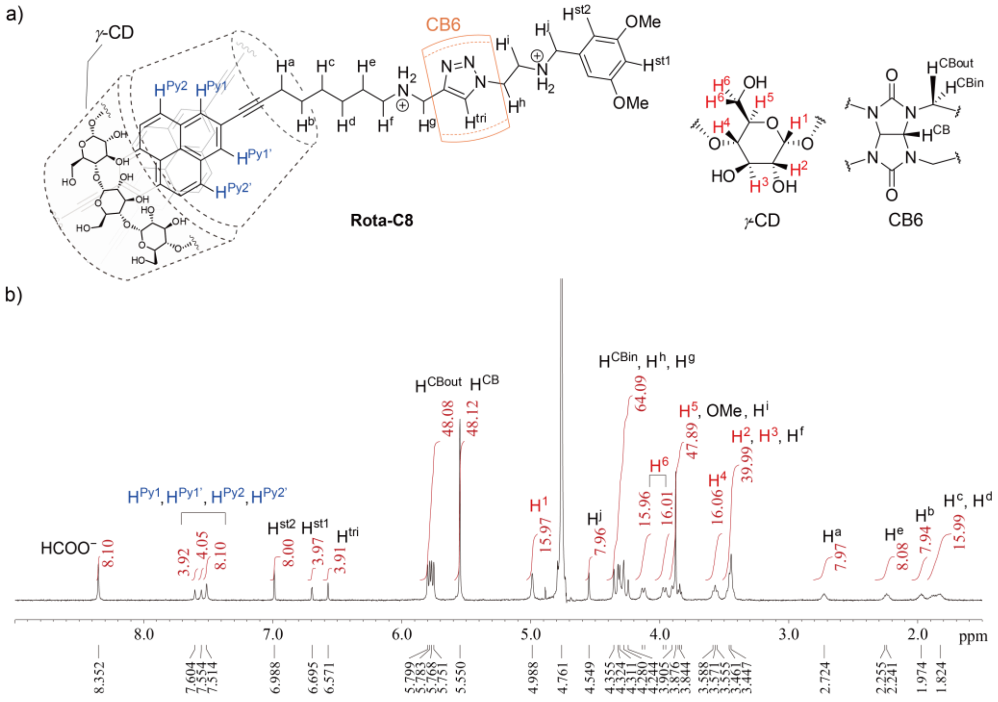

Figure S15. ^{1}H NMR signal assignment of **Rota-C8**. (a) Chemical structures of components of **Rota-C8**. (b) ^{1}H NMR spectrum of **Rota-C8**. Conditions: 2.0 × 10^{-3} M, D_2O, 25 °C, 500 MHz. The H^{Py1} and $H^{Py1'}$ are not equivalent due to the influence of γ-CD rings and the other alkynylpyrene axis (see Figure S25). Similarly, the H^{Py2} and $H^{Py2'}$ are also not equivalent.

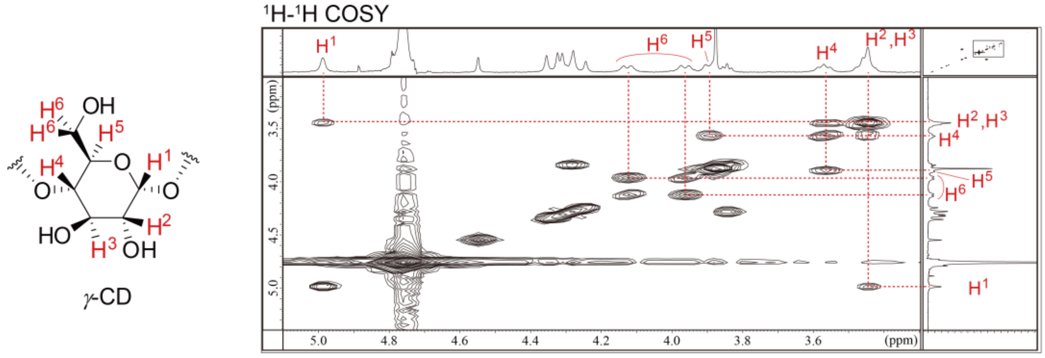

Figure S16. Assignment of ^{1}H NMR signals of γ-CD-ring protons of **Rota-C8**. Chemical structure of γ-CD and ^{1}H-^{1}H COSY NMR spectrum of **Rota-C8**. Conditions: 2.0×10^{-3} M, D_2O, 25 °C, 500 MHz.

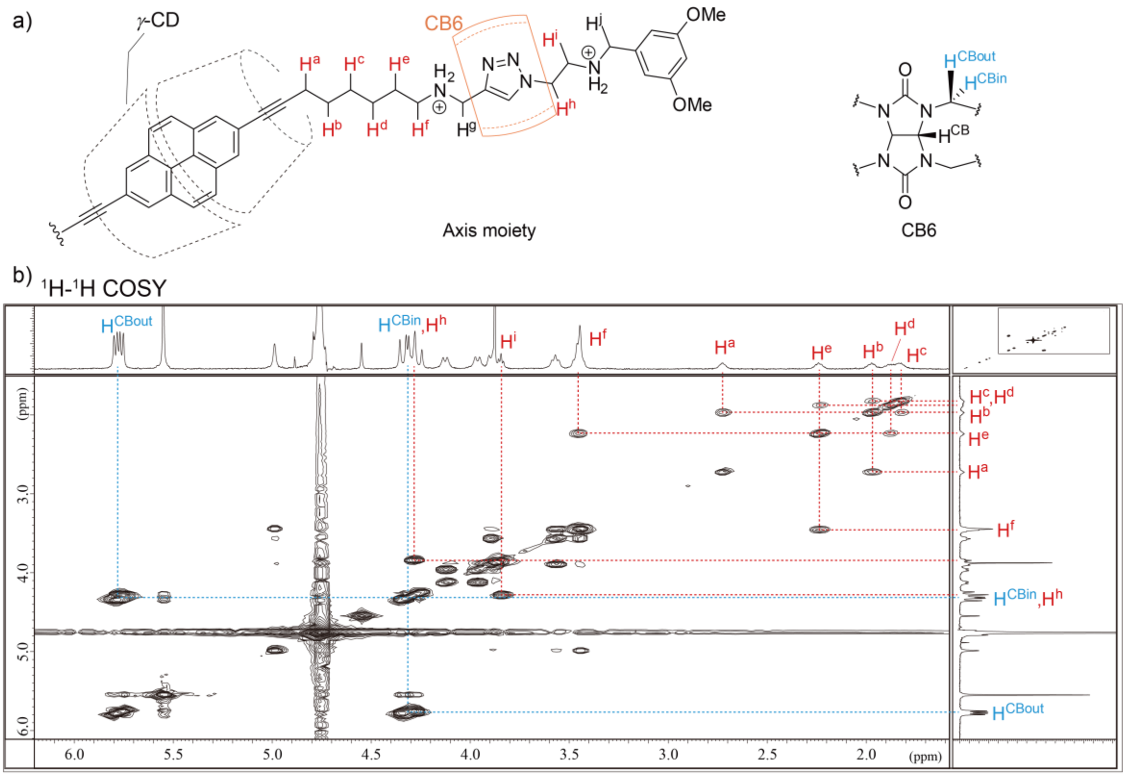

Figure S17. Assignment of ^{1}H NMR signals of methylene-linker and CB6-ring protons of **Rota-C8**. (a) Chemical structures of axis moiety of **Rota-C8** and CB6. (b) ^{1}H-^{1}H COSY NMR spectrum of **Rota-C8**. Conditions: 2.0×10^{-3} M, D_2O, 25 °C, 500 MHz.

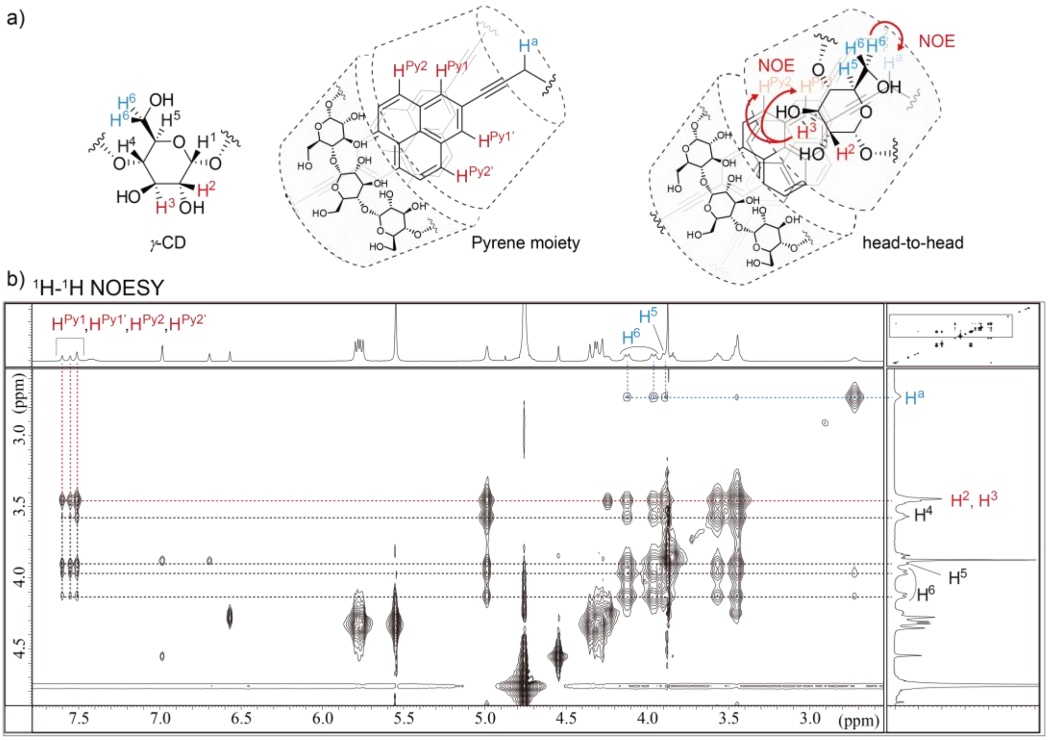

Figure S18. Assignment of head-to-head orientation of **Rota-C8**. (a) Chemical structures of components of **Rota-C8**. (b) ^{1}H-^{1}H NOESY NMR spectrum of **Rota-C8**. Correlation between axis and γ-CD were observed. Conditions: 2.0×10^{-3} M, D_2O, 25 °C, 500 MHz.

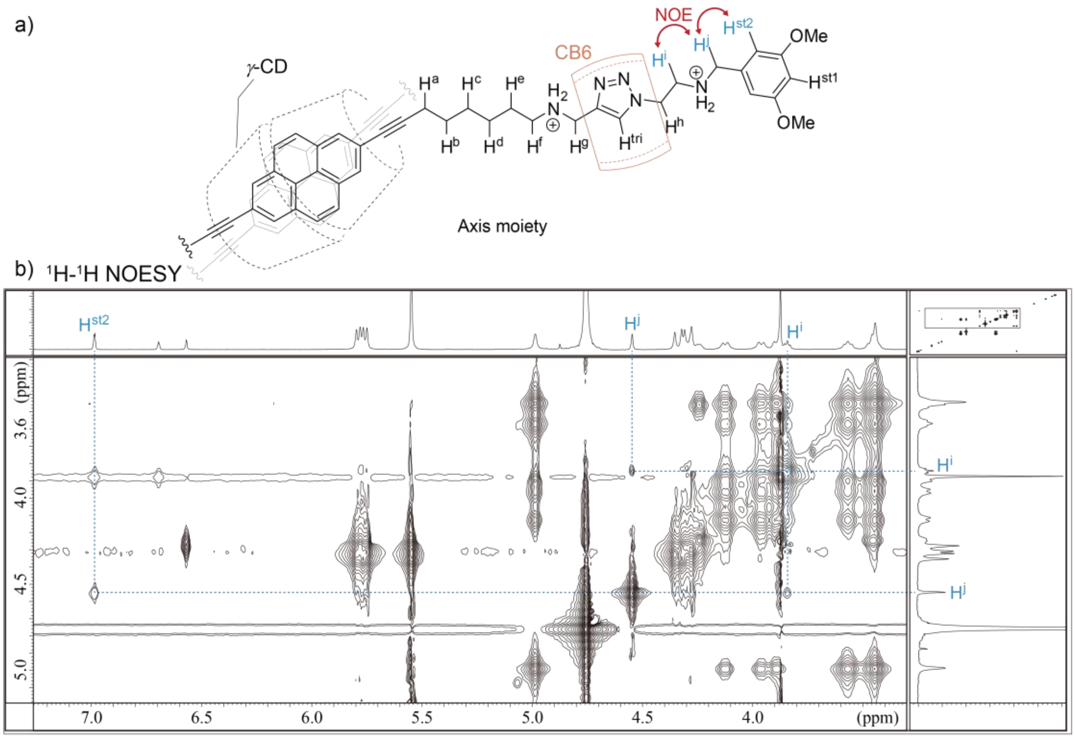

Figure S19. Assignment of ^{1}H NMR signals of methylene-linker and stopper protons of **Rota-C8**. (a) Chemical structure of components of **Rota-C8**. (b) ^{1}H-^{1}H NOESY NMR spectrum of **Rota-C8**. Conditions: 2.0×10^{-3} M, D_2O, 25 °C, 500 MHz.

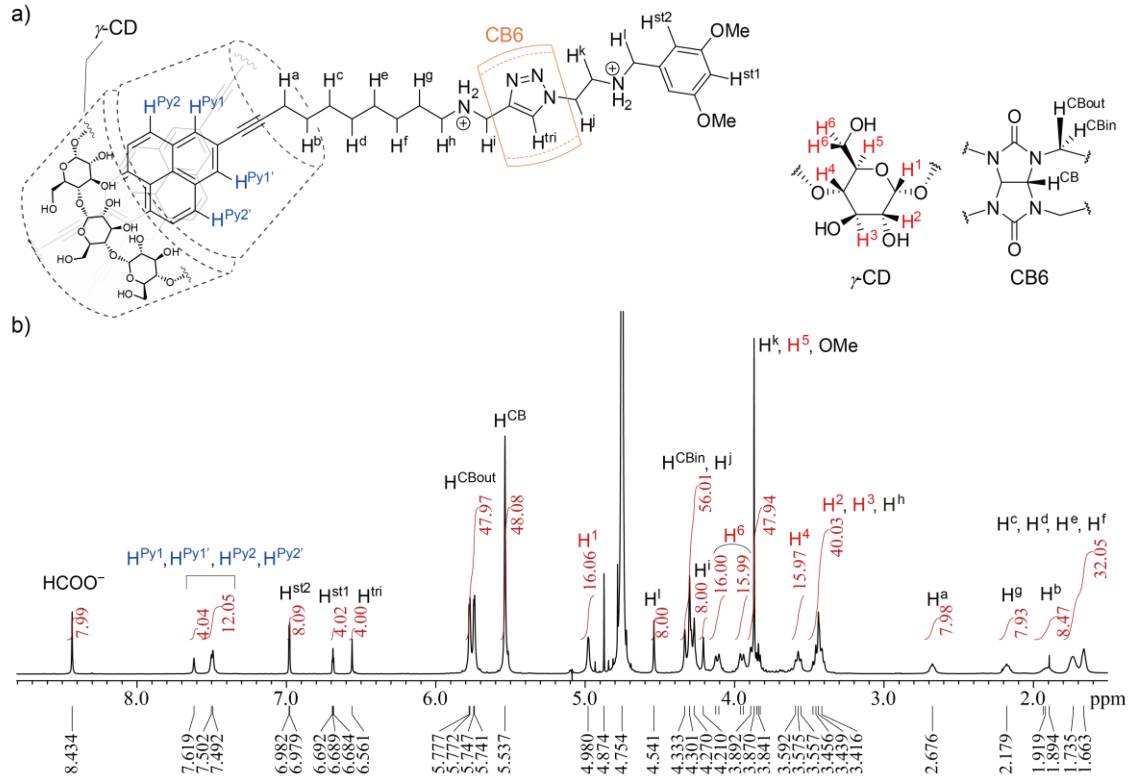

Figure S20. ^{1}H NMR signal assignment of **Rota-C10**. (a) Chemical structures of components of **Rota-C10**. (b) ^{1}H NMR spectrum of **Rota-C10**. Conditions: 2.0×10^{-3} M, D_2O, 25 °C, 500 MHz. The H^{Py1} and $H^{Py1'}$ are not equivalent due to the influence of γ-CD rings and the other alkynylpyrene axis (see Figure S25). Similarly, the H^{Py2} and $H^{Py2'}$ are also not equivalent.

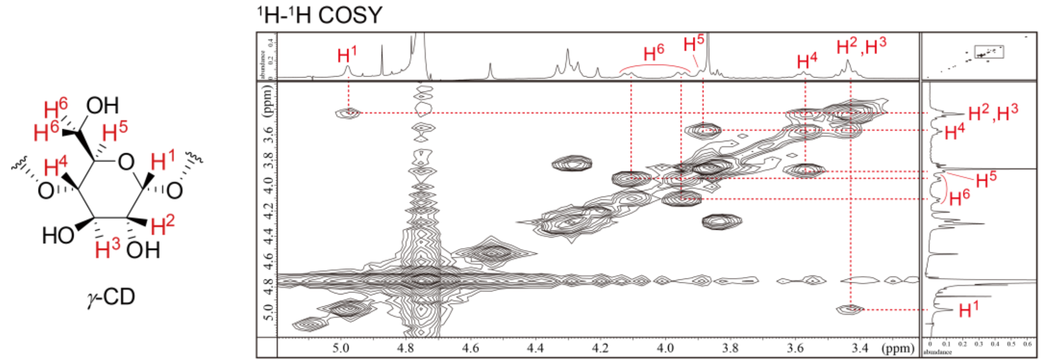

Figure S21. Assignment of ^{1}H NMR signals of γ-CD-ring protons of **Rota-C10**. Chemical structure of γ-CD and ^{1}H-^{1}H COSY NMR spectrum of **Rota-C10**. Conditions: 2.0×10^{-3} M, D_2O, 25 °C, 500 MHz.

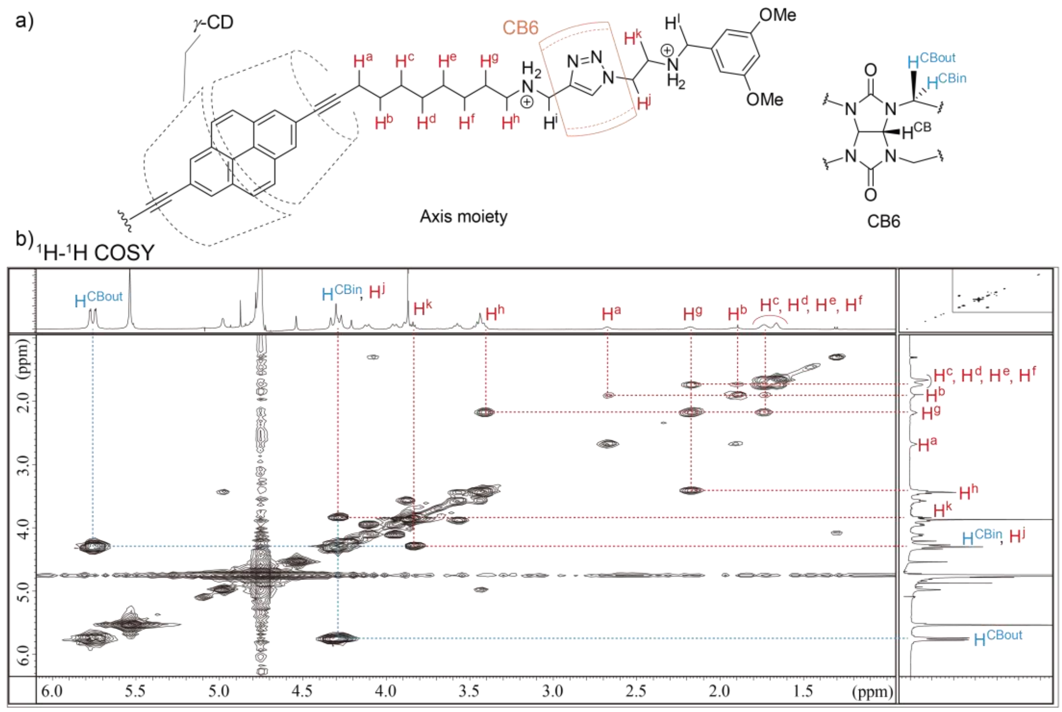

Figure S22. Assignment of ^{1}H NMR signals of methylene-linker and CB6-ring protons of **Rota-C10**. (a) Chemical structures of axis moiety of **Rota-C10** and CB6. (b) ^{1}H-^{1}H COSY NMR spectrum of **Rota-C10**. Conditions: 2.0 × 10^{-3} M, D_2O, 25 °C, 500 MHz.

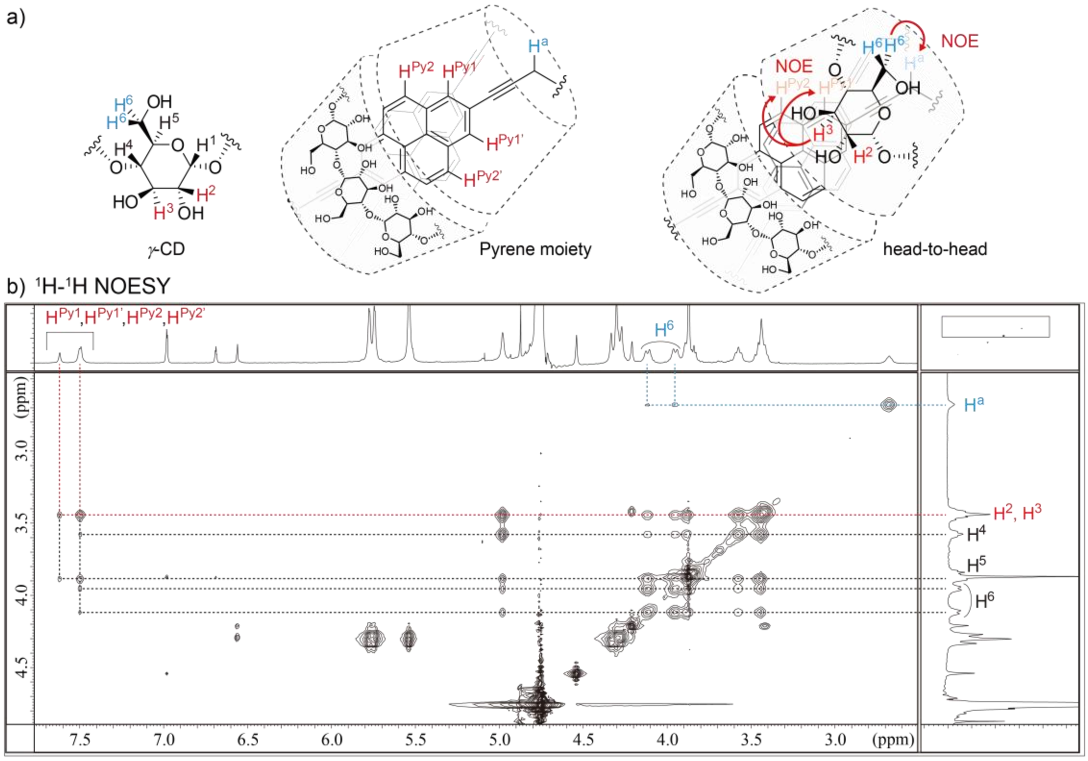

Figure S23. Assignment of head-to-head orientation of **Rota-C10**. (a) Chemical structures of components of **Rota-C10**. (b) ^{1}H-^{1}H NOESY NMR spectrum of **Rota-C10**. Correlation between axis and γ-CD were observed. Conditions: 2.0 × 10^{-3} M, D_2O, 25 °C, 500 MHz.

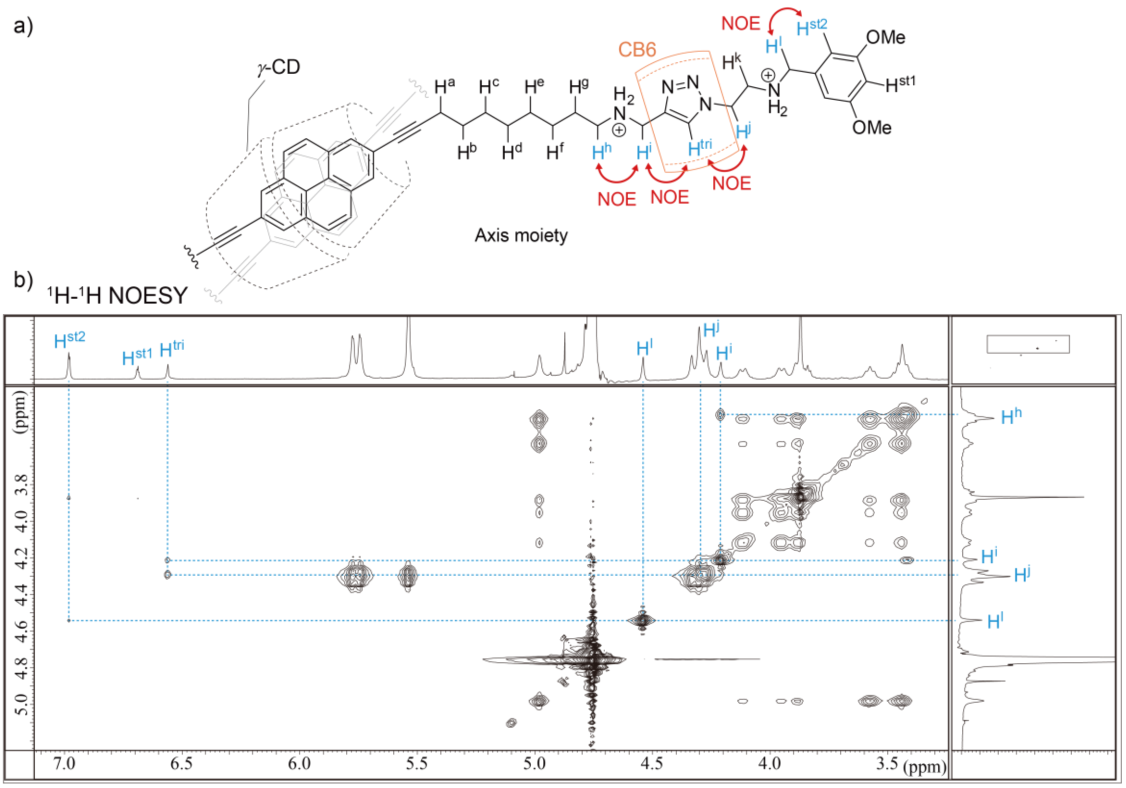

Figure S24. Assignment of ^{1}H NMR signals of methylene-linker and stopper protons of **Rota-C10**. (a) Chemical structure of components of **Rota-C10**. (b) ^{1}H-^{1}H NOESY NMR spectrum of **Rota-C10**. Conditions: 2.0×10^{-3} M, D_2O, 25 °C, 500 MHz.

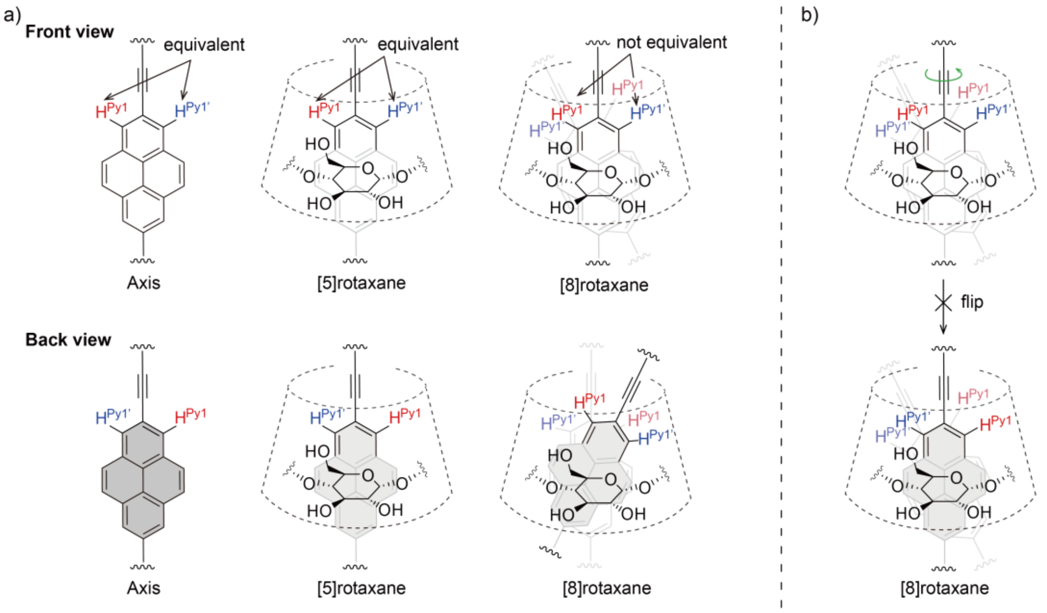

Figure S25. Differences in the protons of the pyrene moiety. (a) Chemical structures of Axis, [5]rotaxane, and [8]rotaxane from the front and back views. (b) Schematic illustration of the rotation of fluorophores. In [8]rotaxane, H^{Py1} and $H^{Py1'}$ are not equivalent in [8]rotaxane because inner pyrene cores do not rotate at least on the ^{1}H NMR timescale.

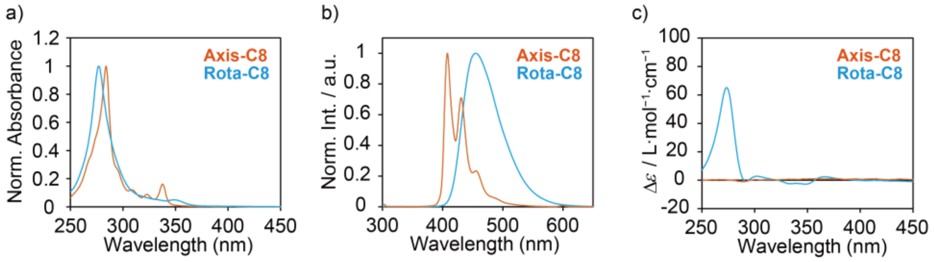

Figure S26. Photophysical properties of **Axis-C8** and **Rota-C8**. (a–c) UV-Vis absorption (a), emission (b), and circular dichroism (c) spectra of **Axis-C8** (orange) and **Rota-C8** (blue). Conditions: [**Axis-C8**] = [**Rota-C8**] = 5.0 × 10^{-6} M, H_2O with 0.1% HCO_2H (**Axis-C8**), H_2O (**Rota-C8**), path length = 10 mm, 25 °C, λ_{ex} = 280 nm.

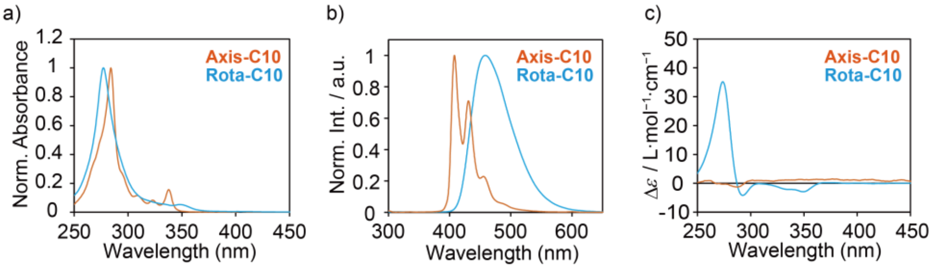

Figure S27. Photophysical properties of **Axis-C10** and **Rota-C10**. (a–c) UV-Vis absorption (a), emission (b), and circular dichroism (c) spectra of **Axis-C10** (orange) and **Rota-C10** (blue). Conditions: [**Axis-C10**] = [**Rota-C10**] = 5.0 × 10^{-6} M, H_2O with 0.1% HCO_2H (**Axis-C10**), H_2O (**Rota-C10**), path length = 10 mm, 25 °C, λ_{ex} = 280 nm.

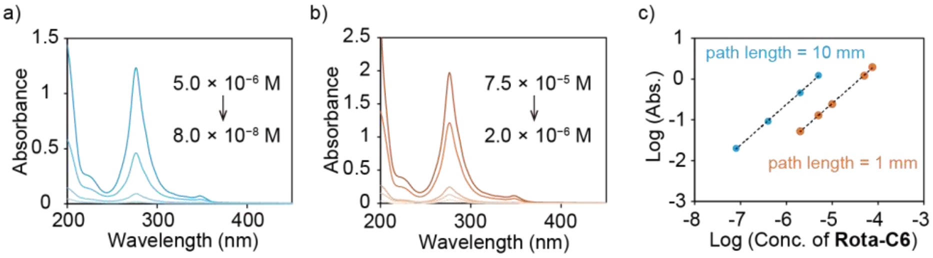

Figure S28. Concentration dependence on absorption property of **Rota-C6**. (a, b) Absorption spectra of **Rota-C6** in various concentrations. (c) Beer's law plots of **Rota-C6**. Conditions: [**Rota-C6**] = 7.5 ×10^{-5} M to 8.0 ×10^{-8} M, H_2O, 25 °C, path length = 10 mm (a) and 1 mm (b).

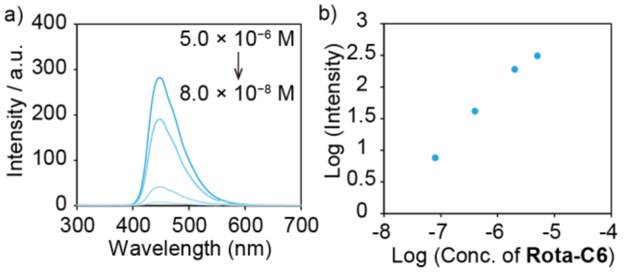

Figure S29. Concentration dependence on emission property of **Rota-C6**. (a) Fluorescence emission spectrum of **Rota-C6** in various concentrations. (b) Emission intensity of **Rota-C6** at 448 nm as a function of concentration in aqueous solution. Conditions: [**Rota-C6**] = 8.0 ×10^{-8} M to 5.0 ×10^{-6} M, H_2O, 25 °C, path length = 10 mm, λ_{ex} = 280 nm.

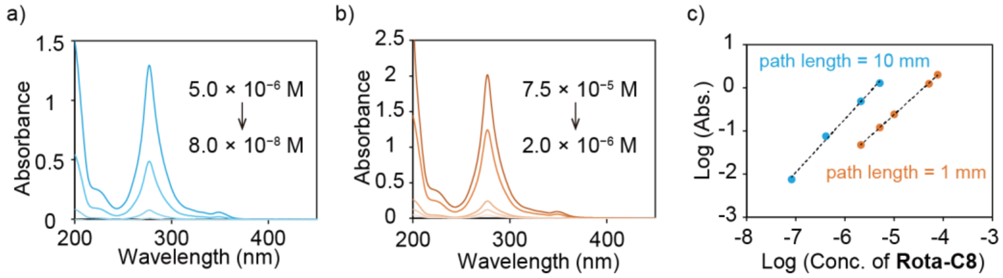

Figure S30. Concentration dependence on absorption property of **Rota-C8**. (a, b) Absorption spectra of **Rota-C8** in various concentrations. (c) Beer's law plots of **Rota-C8**. Conditions: [**Rota-C8**] = 7.5 $\times 10^{-5}$ M to 8.0 $\times 10^{-8}$ M, H_2O, 25 °C, path length = 10 mm (a) and 1 mm (b).

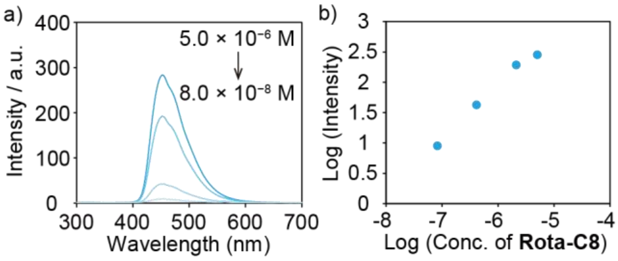

Figure S31. Concentration dependence on emission property of **Rota-C8**. (a) Fluorescence emission spectrum of **Rota-C8** in various concentrations. (b) Emission intensity of **Rota-C8** at 452 nm as a function of concentration in aqueous solution. Conditions: [**Rota-C8**] = 8.0 $\times 10^{-8}$ M to 5.0 $\times 10^{-6}$ M, H_2O, 25 °C, path length = 10 mm, λ_{ex} = 280 nm.

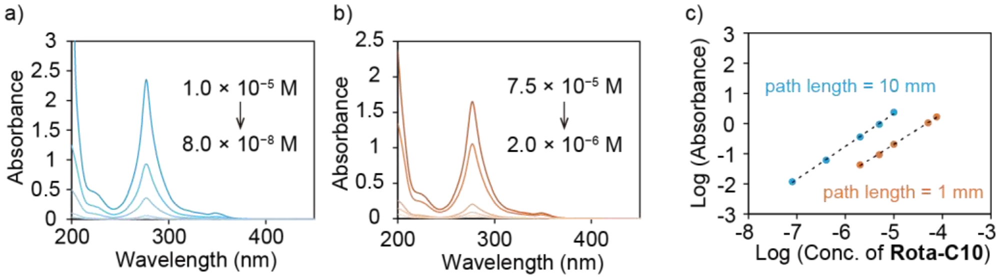

Figure S32. Concentration dependence on absorption property of **Rota-C10**. (a, b) Absorption spectra of **Rota-C10** in various concentrations. (c) Beer's law plots of **Rota-C10**. Conditions: [**Rota-C10**] = 7.5 $\times 10^{-5}$ M to 8.0 $\times 10^{-8}$ M, H_2O, 25 °C, path length = 10 mm (a) and 1 mm (b).

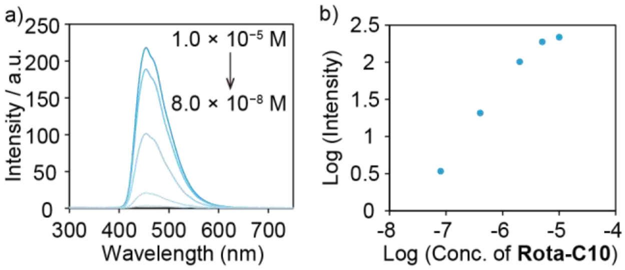

Figure S33. Concentration dependence on emission property of **Rota-C10**. (a) Fluorescence emission spectrum of **Rota-C10** in various concentrations. (b) Emission intensity of **Rota-C10** at 454 nm as a function of concentration in aqueous solution. Conditions: [**Rota-C10**] = 8.0 $\times$ 10^{-8} M to 1.0 $\times$ 10^{-5} M, H_2O, 25 °C, path length = 10 mm, λ_{ex} = 280 nm.

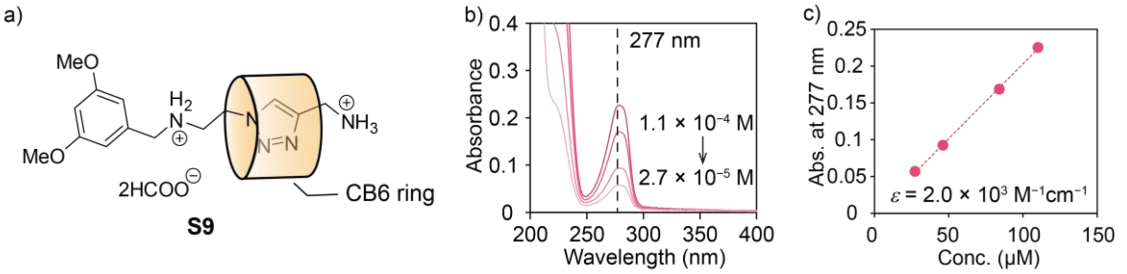

Figure S34. Molar absorption coefficient of stopper and triazole moieties. (a) Structure of reference compounds **S9**. (b) Absorption spectra of **S9** in various concentrations. (c) Beer's plot of **S9**. Conditions: [**S9**] = 1.1 × 10^{-4} M, 8.4 × 10^{-5} M, 4.6 × 10^{-5} M, and 2.7 × 10^{-5} M, H_2O, 25 °C, path length = 10 mm.

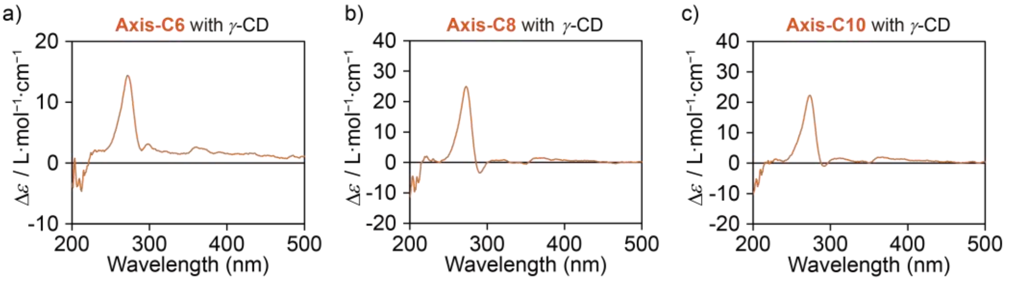

Figure S35. Chiroptical properties of the complexes of Axis and γ-CD. (a–c) Circular dichroism spectrum of complex of **Axis-C6** and γ-CD (a), complex of **Axis-C8** and γ-CD (b), and complex of **Axis-C10** and γ-CD (c). Conditions: [**Axis-C6**] = [**Axis-C8**] = [**Axis-C10**] = [γ-CD] = 5.0 × 10^{-6} M, H_2O with 0.1% HCO_2H, path length = 10 mm, 25 °C.

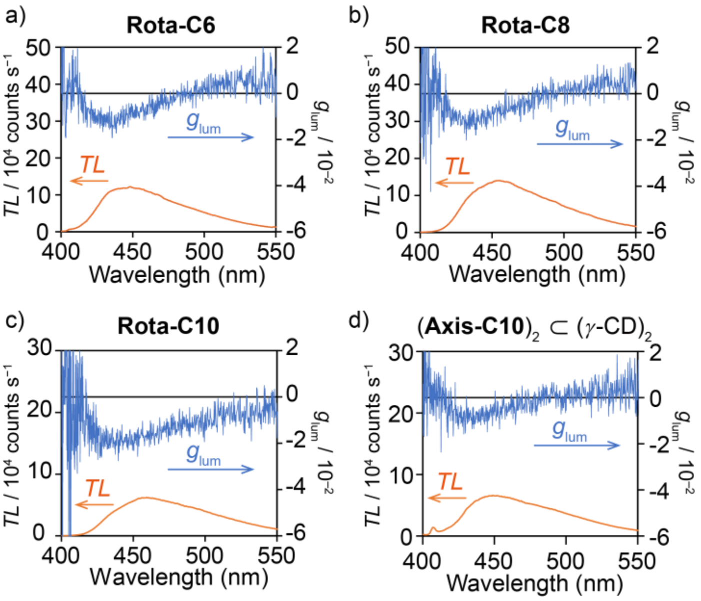

Figure S36. Chiroptical properties of the [8]rotaxanes. (a–d) g_{lum} spectra of **Rota-C6** (a), **Rota-C8** (b), **Rota-C10** (c), inclusion complex of (**Axis-C10**)$_2$ ⊂ (γ-CD)$_2$ (d). Conditions: [**Rota-C6**] = [**Rota-C8**] = [**Rota-C10**] = 5.0 × 10^{-6} M, [**Axis-C10**] = [γ-CD] = 2.0 × 10^{-5} M, H_2O, path length = 10 mm, 25 °C, λ_{ex} = 300–380 nm.

Table S1. Summary of CPL properties of previously reported organic CPL fluorophores exhibiting high B_{CPL} values.

compound[a]	solvent	λ_{abs} [nm]	ε [$\times 10^4$ M^{-1} cm^{-1}]	λ_{em} [nm]	Φ_{lum}	$\lvert g_{lum}\rvert$ [$\times 10^{-3}$]	B_{CPL} [M^{-1} cm^{-1}]	reference
**R1**	cyclohexane	420	7.2	436	0.45	9.5	154	1
**R2**	CH_2Cl_2	310	9.1	456	0.28	10	127	2
**R3**	CH_2Cl_2	291	8.4	515	0.82	17	583	3
**R4**	CH_2Cl_2	457	14	480	0.39	4.2	113	4
**R5**	THF	510	13	558	0.68	14	610	5,6
**R6**	CH_2Cl_2	472	14	684	0.10	45	304	7
**R7**	CH_2Cl_2	---	---	563	0.45	7.9	168	8
**R8**	$CHCl_3$	276	11	378	0.60	4.9	161	9
**R9**	$CHCl_3$	---	---	---	0.28	9.4	255	10
**R10**	CH_2Cl_2	331	---	---	0.84	7.4	189	11
**R11**	CH_2Cl_2	334	11	452	0.90	1.9	101	12
**R12**	$CHCl_3$	356	8.6	469	0.39	23	386	13
**R13**	$CHCl_3$	351	11	452	0.75	7.6	302	14
**R14**	$CHCl_3$	360	19	402	0.75	2.8	194	15
**R15**	$CHCl_3$	376	16	415	0.60	2.5	120	16
**R16**	toluene	511	4.7	602	0.68	13	207	17
**R17**	toluene	503	9.3	627	0.41	30	573	18
**R18**	CH_2Cl_2	479	18	580	0.45	9.4	369	19
**R19**	$CHCl_3$	631	6.1	663	0.58	9	159	20
**R20**	THF	---	---	---	0.45	16	190	21
**R21**	CH_2Cl_2	360	17	540	0.25	34	723	22
**R22**	CH_2Cl_2	386	9.9	565	0.44	6.2	110	23
**R23**	CH_2Cl_2	346	14	480	0.52	9.6	340	24
**R24**	99% H_2O	346	2.1	475	0.60	53	339	25
**R25**	CH_2Cl_2	345	4.5	495	0.32	17	122	26
**R26**	CH_2Cl_2	350	12	455	0.38	15	364	27

[a]The structures of compounds were shown in Figures S37, S38, and S39.

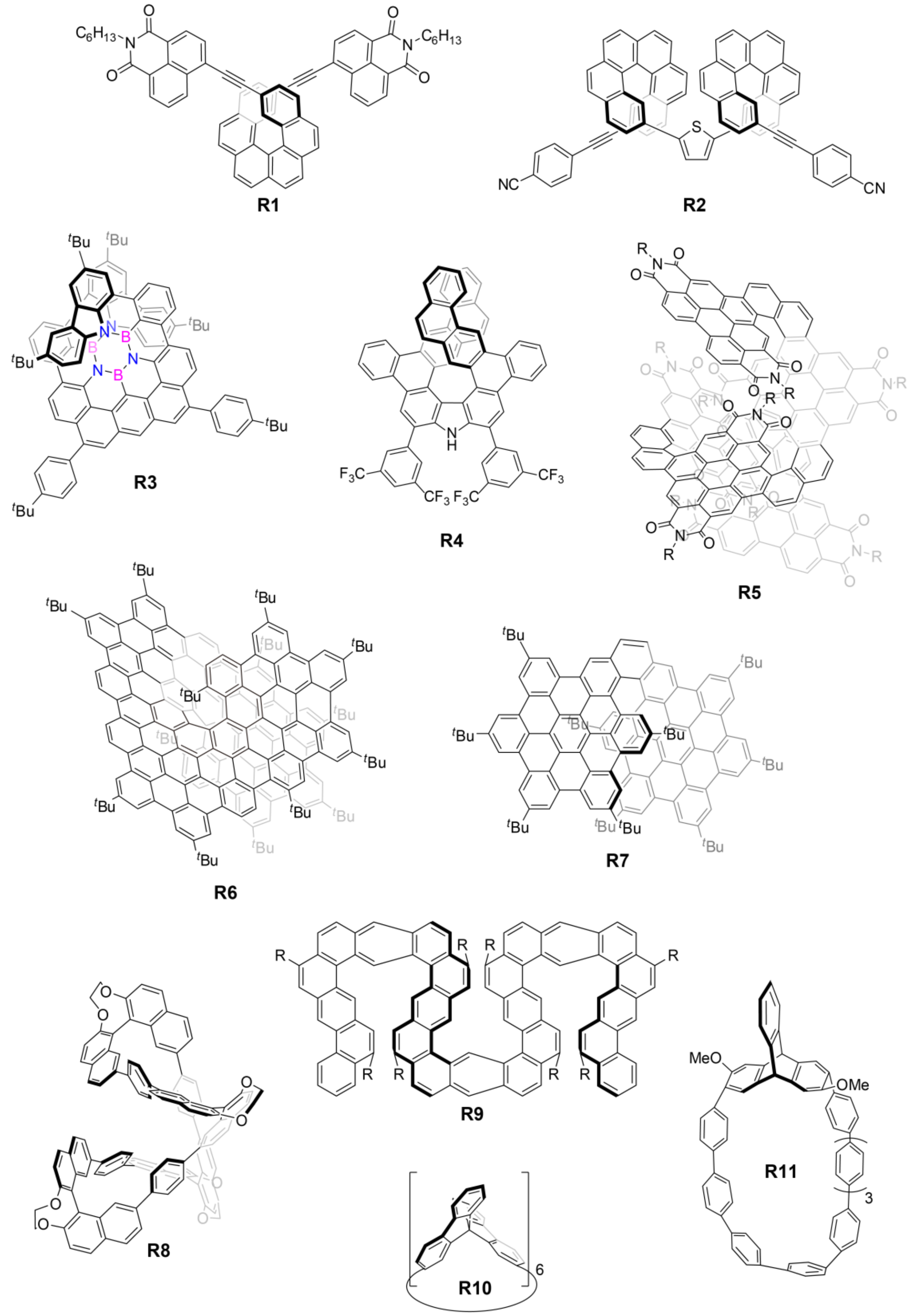

Figure S37. Structures of reference compounds **R1–R11**.

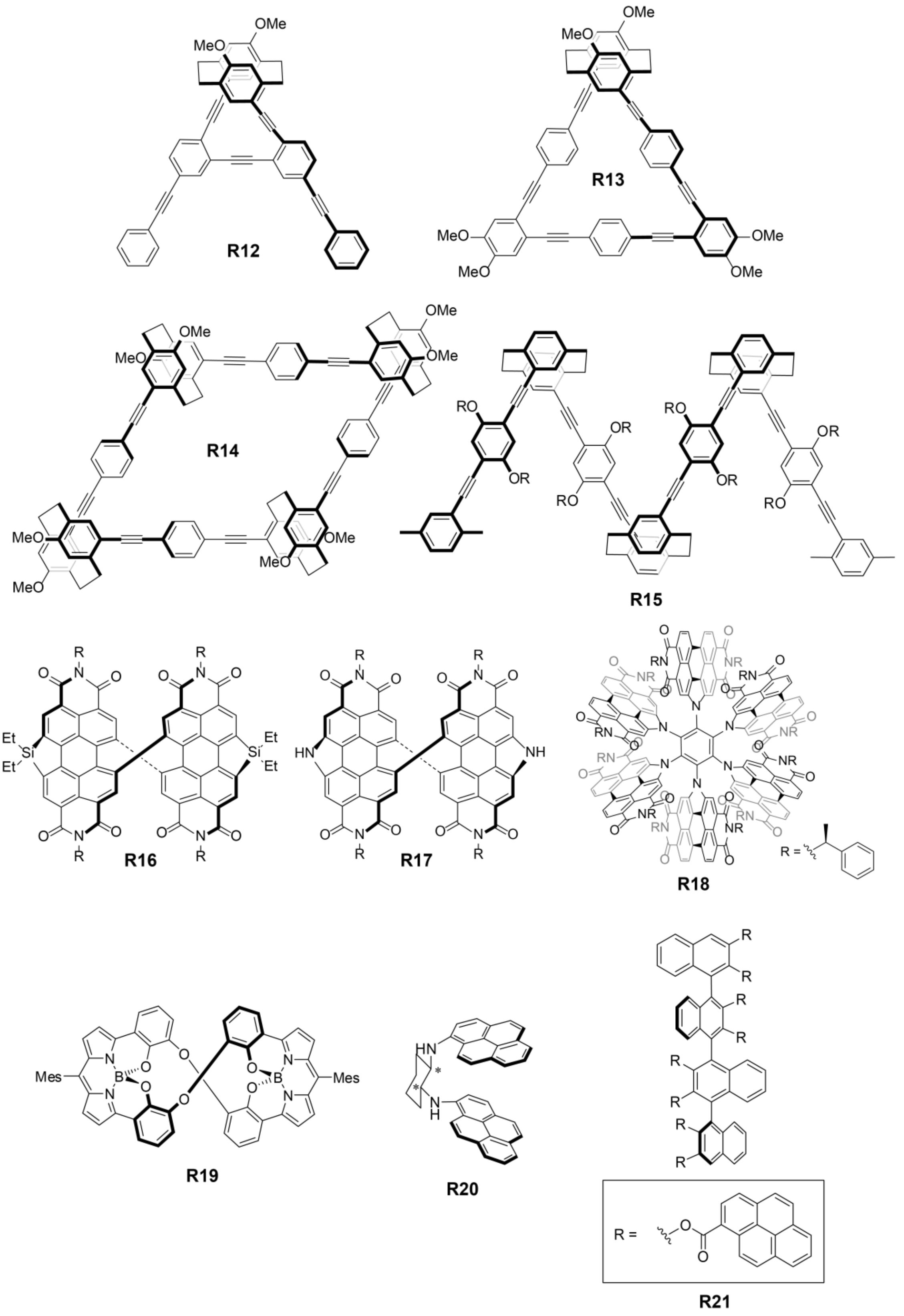

Figure S38. Structures of reference compounds **R12–R21**.

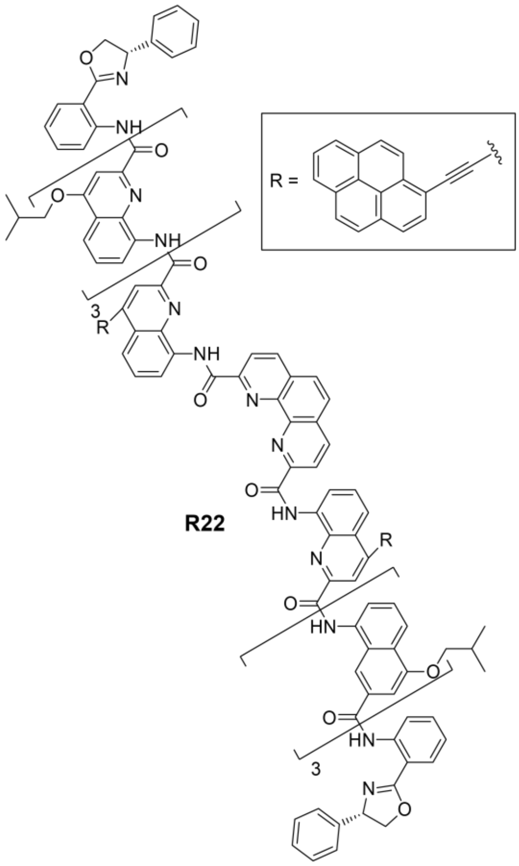

R22

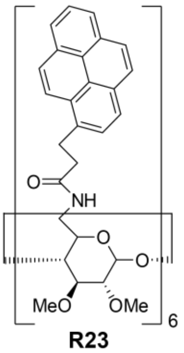

R23

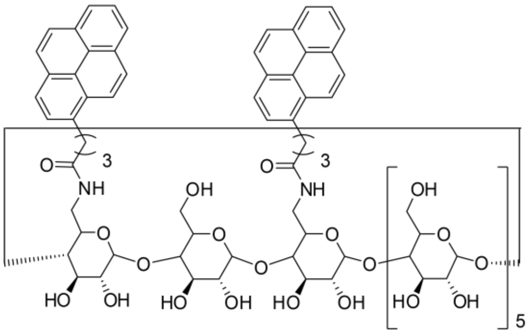

R24

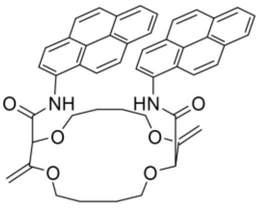

R25

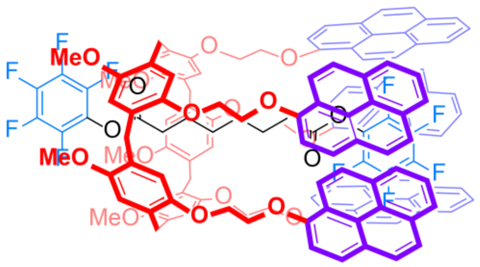

R26

Figure S39. Structures of reference compounds **R22–R26**.

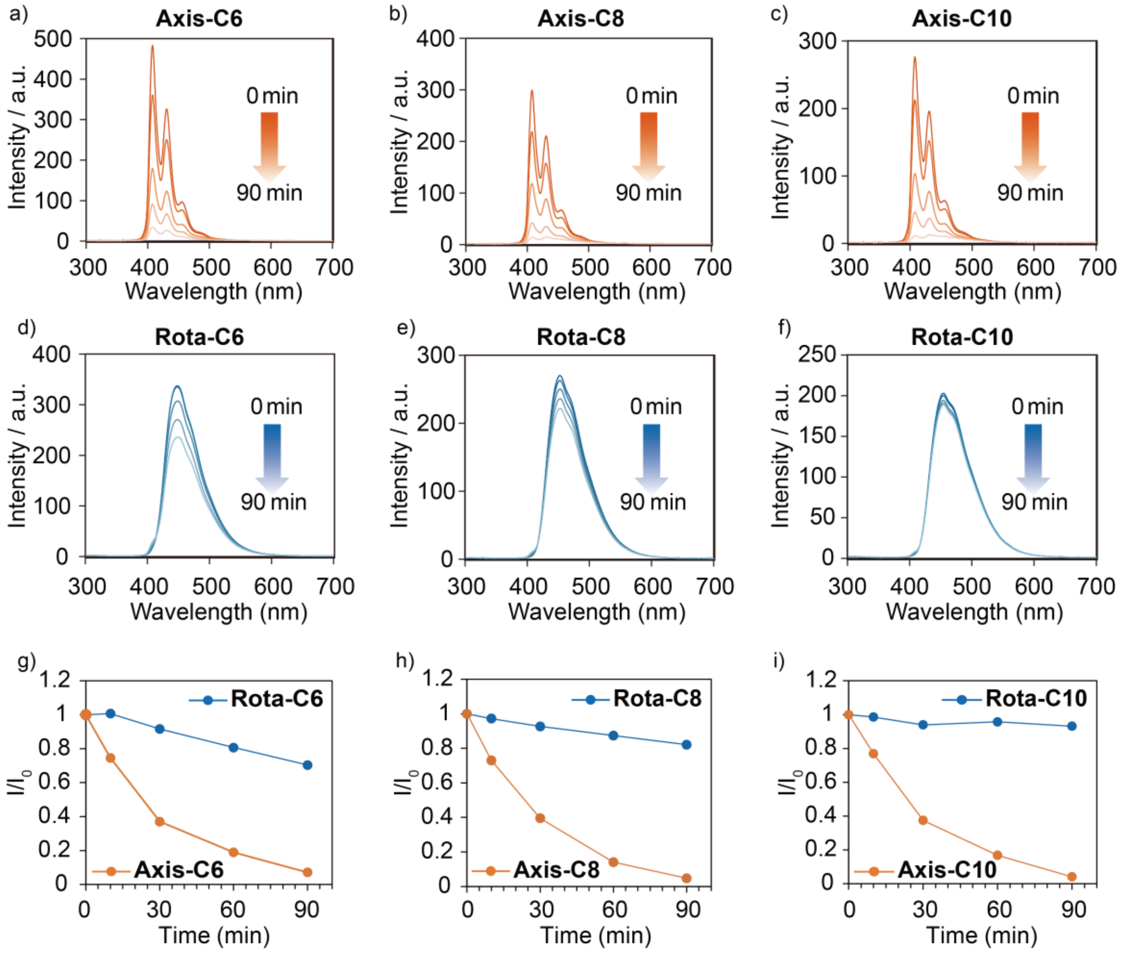

Figure S40. Photostability of [8]rotaxanes. Changes of emission spectra of (a) **Axis-C6**, (b) **Axis-C8**, (c) **Axis-C10**, (d) **Rota-C6**, (e) **Rota-C8**, and (f) **Rota-C10** during irradiation of high-pressure mercury lamp. Conditions: [**Axis-C6**] = [**Axis-C8**] = [**Axis-C10**] = 5.0 × 10^{-6} M in H_2O with 0.1% HCO_2H, [**Rota-C6**] = [**Rota-C8**] = [**Rota-C10**] = 5.0 × 10^{-6} M in H_2O, path length = 10 mm, 25 °C. Light source: 250 W high-pressure mercury lamp. (g–i) Emission changes of (g) **Axis-C6** and **Rota-C6**, (h) **Axis-C8** and **Rota-C8**, and (i) **Axis-C10** and **Rota-C10** over the time.

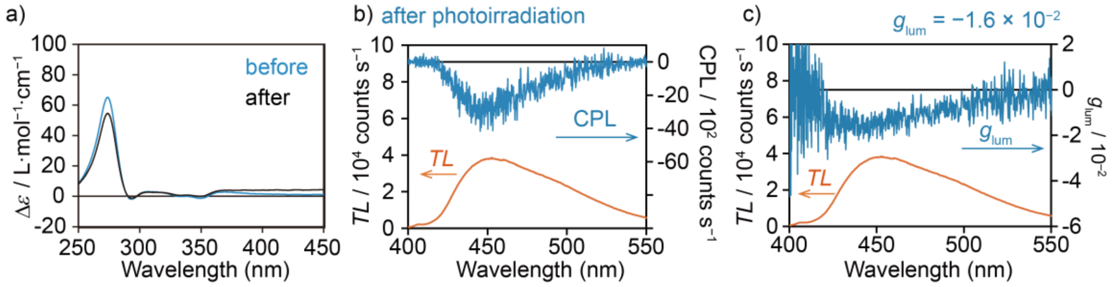

Figure S41. Chiroptical properties of **Rota-C10** before and after the photostability experiment. (a) CD spectrum of **Rota-C10** before and after photoirradiation for 90 min. (b) CPL spectrum of **Rota-C10** after photoirradiation for 90 min. (c) g_{lum} spectrum of **Rota-C10** after photoirradiation for 90 min. Conditions: [**Rota-C10**] = 5.0 × 10^{-6} M in H_2O, path length = 10 mm, 25 °C. Light source: 250 W high-pressure mercury lamp.

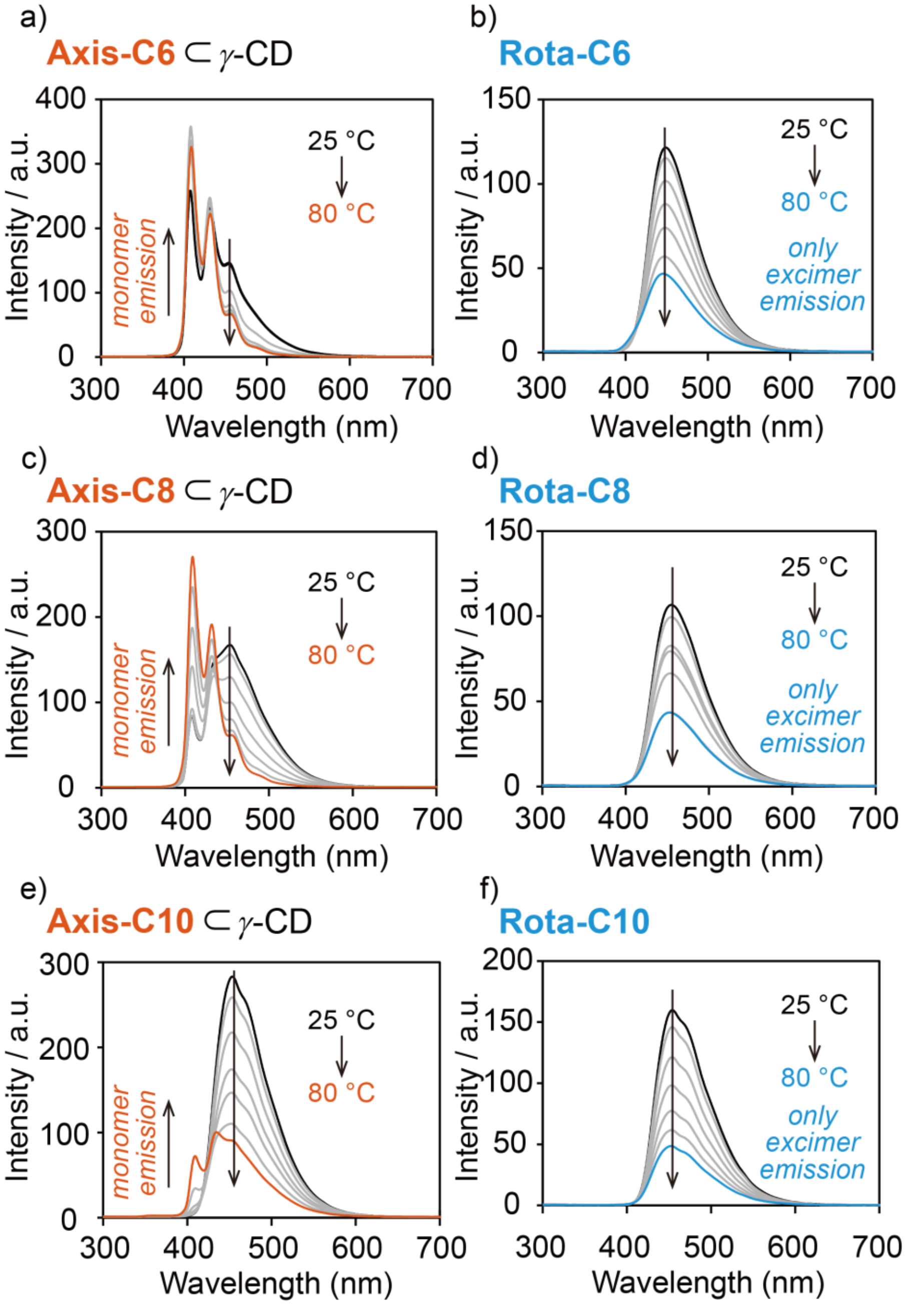

Figure S42. Temperature dependence on emission properties of complexes of axis and γ-CD and [8]rotaxanes. (a–f) Emission spectra of inclusion complex of **Axis-C6** and γ-CD (a), **Rota-C6** (b), inclusion complex of **Axis-C8** and γ-CD (c), **Rota-C8** (d), inclusion complex of **Axis-C10** and γ-CD (e), and **Rota-C10** (f) in various temperatures. Conditions: [**Axis-C6**] = [**Axis-C8**] = [**Axis-C10**] = [γ-CD] = 5.0 $\times 10^{-6}$ M in H_2O with 0.1% HCO_2H, [**Rota-C6**] = [**Rota-C8**] = [**Rota-C10**] = 5.0 $\times 10^{-6}$ M in H_2O, 25 °C to 80 °C, path length = 10 mm, λ_{ex} = 280 nm.

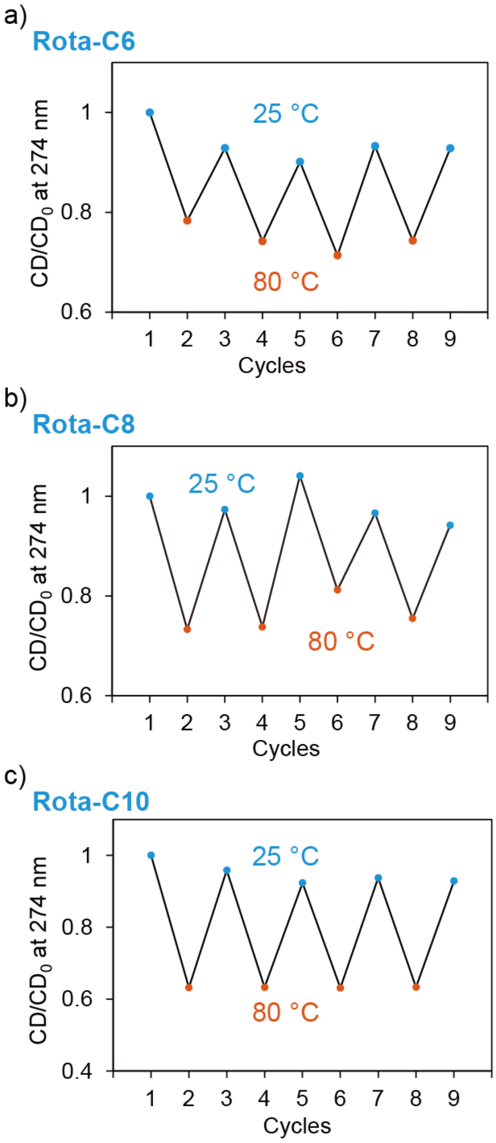

Figure S43. Thermal responses of circular dichroism properties of [8]Rotaxanes. (a-c) Changes of circular dichroism at 274 nm. (a) **Rota-C6**, (b) **Rota-C8**, (c) **Rota-C10**. Conditions: [**Rota-C6**] = [**Rota-C8**] = [**Rota-C10**] = 5.0 ×10^{-6} M, H_2O, 25 °C and 80 °C, path length = 10 mm.

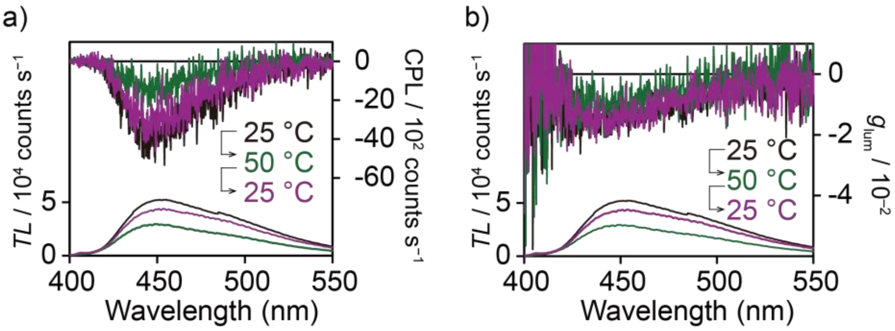

Figure S44. CPL property of **Rota-C10** at high temperature. (a) CPL spectrum of **Rota-C10.** (b) g_{lum} spectrum of **Rota-C10**. Conditions: [**Rota-C10**] = 5.0 ×10^{-6} M, H_2O, 25 °C and 50 °C, path length = 10 mm.

2. Experimental section

General

^{1}H and ^{13}C NMR spectra were collected on JEOL ECA500 spectrometers at 25 °C by using tetramethylsilane (TMS) and sodium 3-(trimethylsilyl)-1-propanesulfonate (DSS) as an internal reference. ESI-HRMS analyses were carried out on a Thermo LTQ Orbitrap XL ETD mass spectrometer by using solutions of the analytes with sodium trifluoroacetate. IR, UV/Vis, fluorescence, and circular dichroism spectra were measured by JASCO spectrometers FT/IR-4X, V-730, FP-6500, J-720WI respectively. The circularly polarized luminescence spectra were recorded with a lab-made spectroscopic measurement system reported previously.[28] Absolute emission quantum yields (Φ_{lum}) and solid-state emission spectra were measured by HAMAMATSU PHOTONICS Quantaurus-QY C11347-01. Melting and decomposition points were measured on a Yanaco MP-500D. Analytical HPLC was conducted using OSAKA SODA UG-120, UG-80, MG-III, ACR (4.6 × 250 mm).

Materials

Reagents were purchased from commercial sources and used without further purification. THF was freshly distilled from sodium benzophenone ketyl before use. The following compounds, 2,7-dibromopyrene (**S1**),[29] 2-nitro-*N*-(2-propyn-1-yl)benzenesulfonamide (**S3**),[30] 2-azido-*N*-[(3,5-dimethoxyphenyl)methyl]-ethylammonium chloride (Stopper)[31] were prepared on the basis of the procedure reported in the literatures previously.

Synthesis

2,7-Bis(6-hydroxyhexyn-1-yl)pyrene (S2)

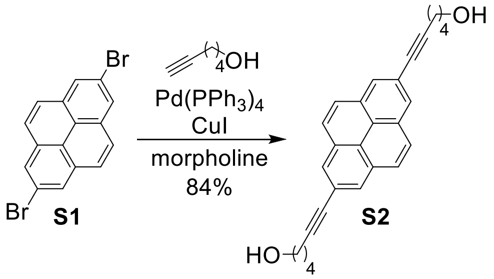

After morpholine (3 mL) had been degassed by freeze-pump-thaw cycling at 3 times, 2,7-dibromopyrene (**S1**) (108 mg, 300 μmol), 5-hexyn-1-ol (118 mg, 1.20 mmol), CuI (5.80 mg, 30.5 μmol) and $Pd(PPh_3)_4$ (35.1 mg, 30.4 μmol) were dissolved in the morpholine solvent at room temperature. The reaction mixture was stirred for 1.5 h at 85 °C. The mixture was concentrated by a rotary evaporator, and subjected to silica gel column chromatography (eluent: CH_2Cl_2 only to $CHCl_3$/MeOH = 30:1) to give a crude product of **S2**. This product was dispersed with *n*-hexane (20 mL) and the resulting precipitate was collected by filtration. The precipitate was washed with

MeOH (0.5 mL) and *n*-hexane (5 mL), and dried under vacuum to give **S2** (99.0 mg, 84%) as a pale beige solid. mp 153.5−156.4 °C; IR (KBr) ν_{max} = 3291, 2933, 2862, 2228, 1913, 1782, 1602, 1523, 1475 cm^{-1}; ^{1}H NMR ($CDCl_3$, 500 MHz) δ = 8.17 (s, 4H), 7.97 (s, 4H), 3.78 (td, *J* = 11.6, 6.2 Hz, 4H), 2.59 (t, *J* = 6.8 Hz, 4H), 1.88−1.77 (m, 8H), 1.36 (t, *J* = 5.4 Hz, 2H); ^{13}C NMR ($CDCl_3$, 125 MHz) δ = 131.0, 128.1, 127.4, 123.6, 121.6, 90.6, 81.4, 62.6, 32.0, 25.1, 19.4; HRMS (ESI-TOF): calcd for $C_{28}H_{26}O_2Na$ [M+Na]$^+$ 417.1825; found 417.1831.

2,7-Bis{6-[*N*-(2-nitrobenzenesulfonyl)-*N*-(2-propyn-1-yl)amino]hexyn-1-yl}pyrene (S4)

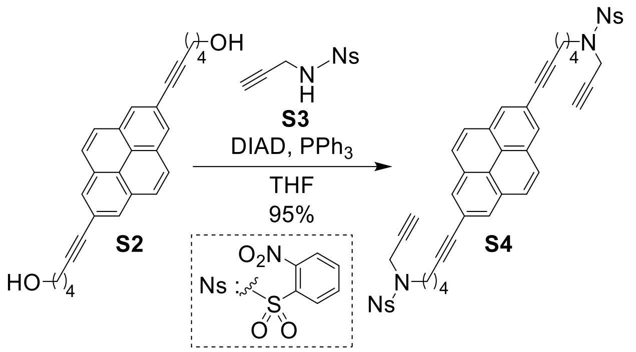

To a dry THF (10 mL) solution of **S2** (62.6 mg, 159 µmol), **S3** (122 mg, 508 µmol), and triphenylphosphine (138 mg, 527 µmol) was slowly added diisopropyl azodicarboxylate (DIAD) (106 µL, 540 µmol) at 0 °C. The mixture was allowed to warm to room temperature, stirred for 1 h, and concentrated by a rotary evaporator. The residue was subjected to silica gel column chromatography (eluent: Toluene/AcOEt = 1:0 to 10:1) to give a crude product of **S4**. This product was dispersed with Et_2O (20 mL) and the resulting precipitate was collected by filtration. The precipitate was washed with Et_2O (10 mL), dried under vacuum to give **S4** (127 mg, 95%) as a white solid. mp 169.1−170.5 °C; IR (KBr) ν_{max} = 3273, 3104, 3044, 2917, 2352, 2228, 2116, 1601, 1536, 1445 cm^{-1}; ^{1}H NMR ($CDCl_3$, 500 MHz) δ = 8.17 (s, 4H), 8.08−8.04 (m, 2H), 7.99 (s, 4H), 7.64−7.59 (m, 6H), 4.26 (d, *J* = 2.4 Hz, 4H), 3.55 (t, *J* = 7.2 Hz, 4H), 2.57 (t, *J* = 6.9 Hz, 4H), 2.18 (t, *J* = 2.4 Hz, 2H), 1.91−1.86 (m, 4H), 1.75−1.70 (m, 4H); ^{13}C NMR ($CDCl_3$, 125 MHz) δ = 148.3, 133.6, 132.7, 131.6, 131.0, 130.9, 128.1, 127.5, 124.2, 123.6, 121.6, 90.1, 81.7, 76.8, 73.9, 46.4, 36.3, 33.4, 26.4, 25.4, 19.1; HRMS (ESI-TOF): calcd for $C_{46}H_{38}N_4O_8S_2Na$ [M+Na]$^+$ 861.2023; found 861.2022.

Axis-C6

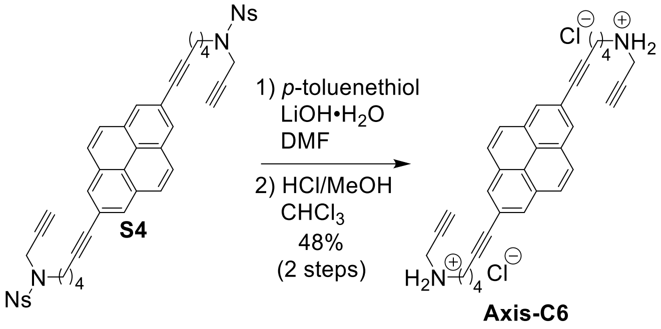

To a DMF (2 mL) solution of **S4** (113 mg, 135 μmol) and LiOH·H_2O (60.0 mg, 1.43 mmol) was slowly added *p*-toluenethiol (178 mg, 1.44 mmol) in DMF (2 mL) at 0 °C. The mixture was allowed to warm to room temperature, and additionally stirred for 50 min. To the resultant mixture was added CH_2Cl_2 (15 mL) and the resultant precipitate was removed by filtration through a celite bed. The filtrate was concentrated by a rotary evaporator and subjected to silica gel column chromatography (eluent: CH_2Cl_2/MeOH = 1:0 to 5:1) to give 2,7-bis{6-[*N*-(2-propyn-1-yl)amino]hexyn-1-yl}pyrene (33.7 mg) as a white solid. To a $CHCl_3$ (1 mL) solution of the product (33.7 mg) was added 5% HCl/MeOH (1 mL) at room temperature. After stirring for 10 min, Et_2O (10 mL) was added to the resultant solution to precipitate a white solid. The solid was collected by filtration, and dried under vacuum to give **Axis-C6** (35.1 mg, 48%; 2 steps) as a white solid. Decomposition point 229.0 °C; IR (KBr) ν_{max} = 3226, 2934, 2779, 2683, 2579, 2428, 2130, 1603, 1440 cm^{-1}; ^1H NMR (CD_3OD, 500 MHz) δ = 8.21 (s, 4H), 8.06 (s, 4H), 3.99 (d, J = 2.6 Hz, 4H), 3.27 (t, J = 2.6 Hz, 2H), 3.23 (t, J = 7.9 Hz, 4H), 2.66 (t, J = 6.8 Hz, 4H), 2.02−1.96 (m, 4H), 1.84−1.78 (m, 4H); ^{13}C NMR (CD_3OD, 125 MHz) δ= 132.5, 129.1, 128.6, 124.6, 123.2, 90.6, 82.8, 79.3, 74.6, 47.7, 37.4, 26.7, 26.4, 19.7; HRMS (ESI-TOF): calcd for $C_{34}H_{34}N_2$ $[M-2Cl]^{2+}$ 235.1356; found 235.1359.

Rota-C6

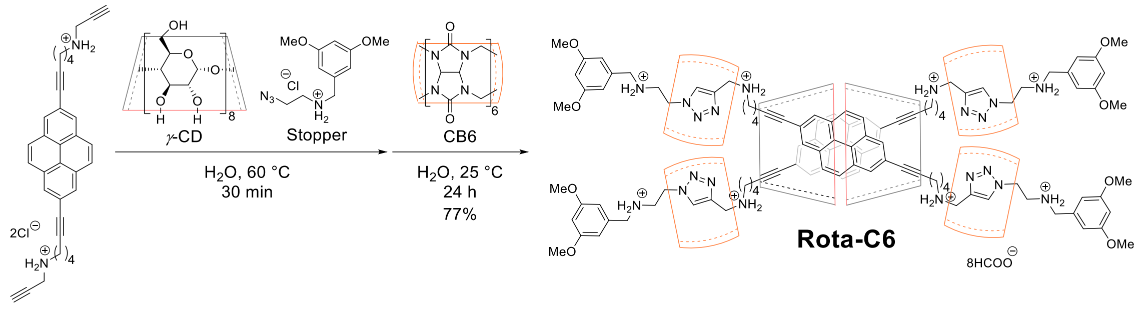

A H_2O (500 μL) solution of **Axis-C6** (1.08 mg, 1.99 μmol), 2-azido-*N*-[(3,5-dimethoxyphenyl)methyl]-ethylammonium chloride (Stopper) (1.25 mg, 4.57 μmol), and γ-CD (78.2 mg, 60.3 μmol) was stirred for 30 min at 60 °C. To the solution was added cucurbit[6]uril (4.90 mg, 5.06 μmol), and the mixture was stirred for 24 h at 25 °C. The resulting solution was filtered by a membrane filter (PTFE, pore size: 0.22 μm), and purified by reverse-phase HPLC to give **Rota-C6** (6.81 mg, 77%) as a white solid. Decomposition point 219.0 °C; IR (KBr) ν_{max} = 3444, 2928, 1739, 1599, 1473 cm^{-1}; ^1H NMR (500 MHz, D_2O) δ = 8.42 (s, 8H), 7.68 (s, 4H), 7.59 (s, 8H), 7.50 (s, 4H), 6.99 (d, J = 1.7 Hz, 8H), 6.69 (t, J = 2.3 Hz, 4H), 6.58 (s, 4H), 5.82−5.76 (m, 48H), 5.55 (s, 48H), 4.99 (s, 16H), 4.55 (s, 8H), 4.36−4.28 (m, 56H), 4.23 (t, J = 6.3 Hz, 8H), 4.14 (d, J = 10.3 Hz, 16H), 4.04 (d, J = 10.9 Hz, 16H), 3.91 (d, J = 9.2 Hz, 16H), 3.87 (s, 24H), 3.82 (t, J = 6.3 Hz, 8H), 3.58−3.54 (m, 24H), 3.49−3.45 (m, 32H), 2.90 (bs, 8H), 2.42 (bs, 8H), 2.26 (bs, 8H); ^{13}C NMR (125 MHz, D_2O) δ = 162.0, 157.9, 157.6, 140.5, 135.6, 130.8, 128.6, 128.5, 128.2, 127.7, 123.0, 121.9, 121.4, 109.2, 104.9, 102.6, 91.4, 82.8, 82.3, 74.2, 73.9, 73.7, 71.6, 61.5, 57.0, 52.9, 52.7, 52.6, 49.8, 48.0, 47.2, 46.7, 43.9, 28.5, 27.3, 20.5, 9.6 ppm, the ^{13}C NMR signals may be imperfect because of the poor signal-noise ratio; HRMS (ESI-TOF): calcd for $C_{352}H_{440}N_{116}O_{136}$ $[M-8HCOO]^{8+}$ 1058.8896, Found 1058.8904.

2,7-Bis(8-hydroxyoctyn-1-yl)pyrene (S5)

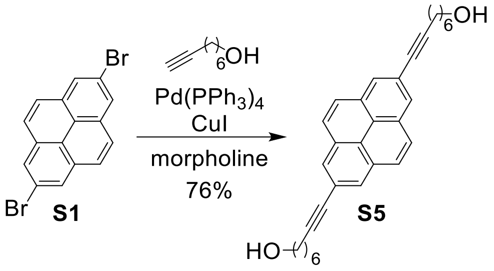

After morpholine (5 mL) had been degassed by freeze-pump-thaw cycling at 3 times, 2,7-dibromopyrene (**S1**) (173 mg, 480 μmol), 7-octyn-1-ol (252 mg, 2.00 mmol), CuI (9.3 mg, 49 μmol) and $Pd(PPh_3)_4$ (55.5 mg, 48.0 μmol) were dissolved in the morpholine solvent at room temperature. The reaction mixture was stirred for 3 h at 80 °C. The mixture was concentrated by a rotary evaporator, and subjected to silica gel column chromatography (eluent: CH_2Cl_2 only to CH_2Cl_2/MeOH = 30:1) to give a crude product of **S5**. This product was dispersed with MeOH (3 mL) and the resulting precipitate was collected by filtration. The precipitate was dried under vacuum to give **S5** (165 mg, 76%) as a white solid. mp 147.5−149.8 °C; IR (KBr) ν_{max} = 3414, 3308, 2929, 2851, 1602, 1462 cm^{-1}; ^1H NMR ($CDCl_3$, 500 MHz) δ = 8.17 (s, 4H), 7.97 (s, 4H), 3.69 (td, J = 11.6, 6.4 Hz, 4H), 2.54 (t, J = 7.1 Hz, 4H), 1.75−1.69 (m, 4H), 1.67−1.62 (m, 4H), 1.61−1.55 (m, 4H), 1.50−1.44 (m, 4H), 1.28 (t, J = 4.6 Hz, 2H); ^{13}C NMR ($CDCl_3$, 125 MHz) δ = 131.0, 128.0, 127.4, 123.6, 121.7, 91.0, 81.2, 63.0, 32.7, 28.8, 28.7, 25.4, 19.5; HRMS (ESI-TOF): calcd for $C_{32}H_{34}O_2Na$ $[M+Na]^+$ 473.2451; found 473.2453.

2,7-Bis{8-[*N*-(2-nitrobenzenesulfonyl)-*N*-(2-propyn-1-yl)amino]octyn-1-yl}pyrene (S6)

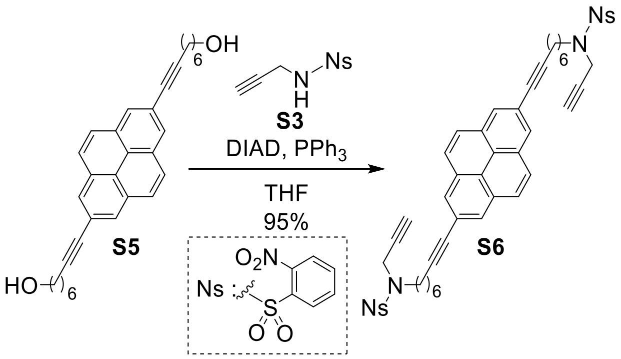

To a dry THF (7 mL) solution of **S5** (146 mg, 324 μmol), **S3** (211 mg, 878 μmol), and triphenylphosphine (236 mg, 901 μmol) was slowly added diisopropyl azodicarboxylate (DIAD) (187 μL, 950 μmol) at 0 °C. The mixture was allowed to warm to room temperature, stirred for 30 min, and concentrated by a rotary evaporator. The residue was subjected to silica gel column chromatography (eluent: Toluene/AcOEt = 1:0 to 10:1) to give a crude product of **S6**. This product was dispersed with Et_2O (20 mL) and the resulting precipitate was collected by filtration. The

precipitate was washed with Et_2O (10 mL), dried under vacuum to give **S6** (275 mg, 95%) as a pale yellow solid. mp 161.2−163.7 °C; IR (KBr) ν_{max} = 3284, 3088, 3041, 2937, 2853, 2235, 1760, 1602, 1542, 1440 cm^{-1}; ^{1}H NMR ($CDCl_3$, 500 MHz) δ = 8.18 (s, 4H), 8.05−8.03 (m, 2H), 7.98 (s, 4H), 7.67−7.60 (m, 6H), 4.22 (d, J = 2.4 Hz, 4H), 3.45 (t, J = 7.4 Hz, 4H), 2.52 (t, J = 7.0 Hz, 4H), 2.16 (t, J = 2.4 Hz, 2H), 1.71−1.64 (m, 8H), 1.59−1.53 (m, 4H), 1.44−1.38 (m, 4H); ^{13}C NMR ($CDCl_3$, 125 MHz) δ = 148.3, 133.6, 132.8, 131.5, 131.0, 130.8, 128.1, 127.4, 124.1, 123.6, 121.7, 90.9, 81.2, 76.8, 73.8, 46.7, 36.2, 28.5, 28.4, 27.3, 26.0, 19.5; HRMS (ESI-TOF): calcd for $C_{50}H_{46}N_4O_8S_2Na$ [M+Na]$^+$ 917.2649; found 917.2648.

Axis-C8

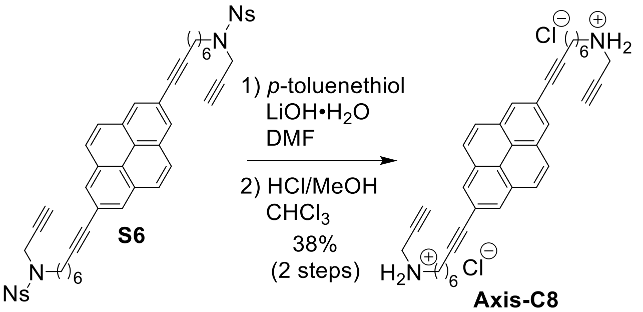

Axis-C8

To a DMF (5 mL) solution of **S6** (237 mg, 265 μmol) and LiOH·H_2O (134 mg, 3.18 mmol) was slowly added *p*-toluenethiol (309 mg, 2.48 mmol) in DMF (4 mL) at room temperature. The mixture was stirred for 1 h. To the resultant mixture was added CH_2Cl_2 (20 mL) and the resultant precipitate was removed by filtration through a celite bed. The filtrate was concentrated by a rotary evaporator and subjected to silica gel column chromatography (eluent: CH_2Cl_2/MeOH = 1:0 to 10:1) to give 2,7-bis{8-[*N*-(2-propyn-1-yl)amino]octyn-1-yl}pyrene (76.7 mg) as a colorless solid. To a $CHCl_3$ (4 mL) solution of the product (76.7 mg) was added 5% HCl/MeOH (1 mL) at room temperature. After stirring for 10 min, Et_2O (20 mL) was added to the resultant solution to precipitate a white solid. The solid was collected by filtration, and dried under vacuum to give **Axis-C8** (59.5 mg, 38%; 2 steps) as a white solid. Decomposition point 208.3 °C; IR (KBr) ν_{max} = 3431, 3223, 2934, 2780, 2594, 2424, 2129, 1603, 1443 cm^{-1}; ^{1}H NMR (CD_3OD, 500 MHz) δ = 8.17 (s, 4H), 8.04 (s, 4H), 3.95 (d, J = 2.4 Hz, 4H), 3.24 (t, J = 2.3 Hz, 2H), 3.13 (t, J = 7.8 Hz, 4H), 2.57 (t, J = 6.9 Hz, 4H), 1.80−1.72 (m, 8H), 1.67−1.61 (m, 4H), 1.55−1.49 (m, 4H); ^{13}C NMR (CD_3OD, 125 MHz) δ = 132.5, 129.0, 128.6, 124.6, 123.4, 91.6, 82.2, 79.2, 74.6, 48.1, 37.3, 29.6, 29.5, 27.1, 27.0, 20.0; HRMS (ESI-TOF): calcd for $C_{38}H_{42}N_2$ [M−2Cl]$^{2+}$ 263.1669; found 263.1669.

Rota-C8

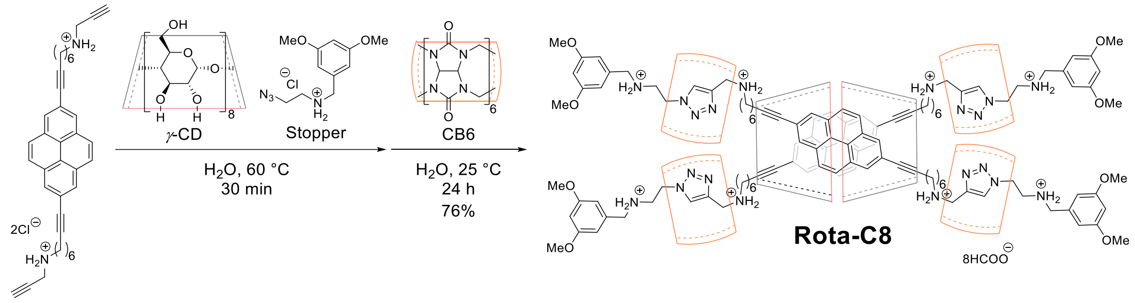

A H_2O (125 μL) solution of **Axis-C8** (323 μg, 0.540 μmol), 2-azido-*N*-[(3,5-dimethoxyphenyl)methyl]-ethylammonium chloride (Stopper) (360 μg, 1.32 μmol), and *γ*-CD (20.5 mg, 15.8 μmol) was stirred for 30 min at 60 °C. To the solution was added cucurbit[6]uril (1.33 mg, 1.34 μmol), and the mixture was stirred for 24 h at 25 °C. The resulting solution was filtered by a membrane filter (PTFE, pore size: 0.22 μm), and purified by reverse-phase HPLC to give **Rota-C8** (1.83 mg, 76%) as a white solid. Decomposition point 216.8 °C; IR (KBr) ν_{max} = 3394, 2917, 2108, 1738, 1598, 1474 cm^{-1}; ^1H NMR (500 MHz, D_2O) δ = 8.35 (s, 8H), 7.60 (s, 4H), 7.55 (s, 4H), 7.51 (s, 8H), 6.99 (s, 8H), 6.69 (s, 4H), 6.57 (s, 4H), 5.80–5.75 (m, 48H), 5.55 (s, 48H), 4.99 (s, 16H), 4.55 (s, 8H), 4.36–4.24 (m, 64H), 4.13 (d, *J* = 9.2 Hz, 16H), 3.96 (d, *J* = 10.3 Hz, 16H), 3.91–3.83 (m, 48H), 3.57 (t, *J* = 8.3 Hz, 16H), 3.46–3.45 (m, 40H), 2.72 (bs, 8H), 2.25 (bs, 8H), 1.97 (bs, 8H), 1.82 (bs, 16H); ^{13}C NMR (125 MHz, D_2O) δ = 170.1, 169.9, 161.8, 157.7, 157.4, 140.2, 135.4, 130.5, 128.2, 127.6, 122.8, 121.9, 121.3, 109.1, 104.8, 102.2, 91.8, 82.5, 82.2, 74.1, 74.0, 73.9, 73.3, 71.4, 71.1, 61.3, 61.2, 57.0, 56.9, 56.7, 52.7, 52.5, 47.1, 46.6, 43.5, 30.9, 27.7, 27.0, 20.6 ppm, the ^{13}C NMR signals may be imperfect because of the poor signal-noise ratio; HRMS (ESI-TOF): calcd for $C_{360}H_{456}N_{116}O_{136}$ $[M-8HCOO]^{8+}$ 1072.9053, Found 1072.9067.

2,7-Bis(10-hydroxydecyn-1-yl)pyrene (S7)

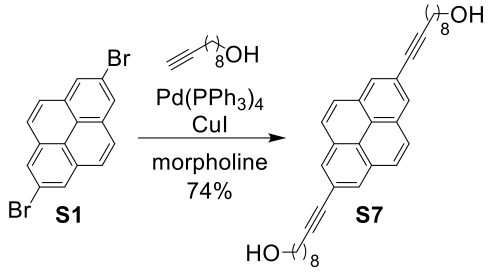

After morpholine (10 mL) had been degassed by freeze-pump-thaw cycling at 3 times, 2,7-dibromopyrene (**S1**) (359 mg, 1.00 mmol), 9-decyn-1-ol (629 mg, 4.08 mmol), CuI (22.9 mg, 120 µmol) and $Pd(PPh_3)_4$ (145 mg, 126 µmol) were dissolved in the morpholine solvent at room temperature. The reaction mixture was stirred for 2 h at 85 °C. The mixture was concentrated by a rotary evaporator, and subjected to silica gel column chromatography (eluent: CH_2Cl_2 only to $CHCl_3$/MeOH = 30:1) to give a crude product of **S7**. This product was dispersed with MeOH (20 mL) and the resulting precipitate was collected by filtration. The precipitate was washed with MeOH (10 mL), dried under vacuum to give **S7** (375 mg, 74%) as a white solid. mp 148.5−149.6 °C; IR (KBr) ν_{max} = 3326, 3043, 2928, 2850, 2226, 1601, 1467 cm^{-1}; ^1H NMR ($CDCl_3$, 500 MHz) δ = 8.18 (s, 4H), 7.98 (s, 4H), 3.66 (dd, J = 11.4, 6.5 Hz, 4H), 2.52 (t, J = 7.2 Hz, 4H), 1.73−1.67 (m, 4H), 1.61−1.57 (bs, 4H), 1.56−1.51 (bs, 4H), 1.40 (bs, 12H), 1.21 (t, J = 5.2 Hz, 2H); ^{13}C NMR ($CDCl_3$, 125 MHz) δ = 131.0, 128.0, 127.4, 123.5, 121.8, 91.2, 81.1, 63.1, 32.8, 29.3, 29.2, 28.9, 28.8, 25.7, 19.6; HRMS (ESI-TOF): calcd for $C_{36}H_{42}O_2Na$ $[M+Na]^+$ 529.3077; found 529.3077.

2,7-Bis{10-[*N*-(2-nitrobenzenesulfonyl)-*N*-(2-propyn-1-yl)amino]decyn-1-yl}pyrene (S8)

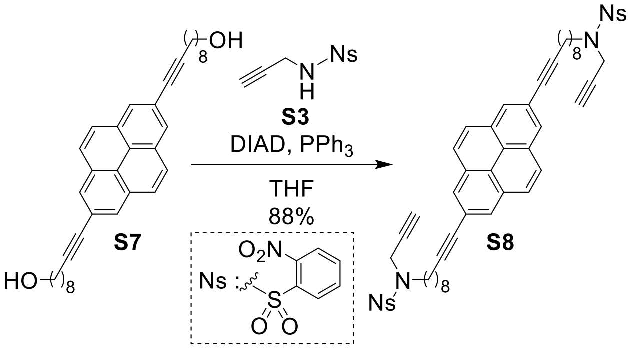

To a dry THF (10 mL) solution of **S7** (260 mg, 513 µmol), **S3** (307 mg, 1.28 mmol), and triphenylphosphine (345 mg, 1.32 mmol) was slowly added diisopropyl azodicarboxylate (DIAD) (270 µL, 1.35 mmol) at 0 °C. The mixture was allowed to warm to room temperature, stirred for 1 h, and concentrated by a rotary evaporator. The residue was subjected to silica gel column chromatography (eluent: Toluene/AcOEt = 1:0 to 10:1) to give a crude product of **S8**. This product

was dispersed with Et_2O (20 mL) and the resulting precipitate was collected by filtration. The precipitate was washed with Et_2O (10 mL), dried under vacuum to give **S8** (428 mg, 88%) as a white solid. mp 130.9−138.7 °C; IR (KBr) ν_{max} = 3289, 3258, 3096, 2923, 2855, 2228, 2117, 1602, 1544, 1466, 1444 cm^{-1}; ^{1}H NMR ($CDCl_3$, 500 MHz) δ = 8.18 (s, 4H), 8.03−8.02 (m, 2H), 7.98 (s, 4H), 7.69−7.61 (m, 6H), 4.20 (d, J = 2.4 Hz, 4H), 3.41 (t, J = 7.4 Hz, 4H), 2.52 (t, J = 7.1 Hz, 4H), 2.15 (t, J = 2.4 Hz, 2H), 1.71−1.65 (m, 4H), 1.64−1.60 (m, 4H), 1.54−1.48 (m, 4H), 1.38−1.33 (m, 12H); ^{13}C NMR ($CDCl_3$, 125 MHz) δ = 148.2, 133.6, 132.8, 131.5, 131.0, 130.8, 128.1, 127.4, 124.1, 123.6, 121.8, 91.2, 81.1, 73.7, 65.9, 46.7, 36.1, 29.0, 28.8, 28.7, 27.3, 26.4, 19.6, 15.3; HRMS (ESI-TOF): calcd for $C_{54}H_{54}N_4O_8S_2Na$ [M+Na]$^+$ 973.3275; found 973.3318.

Axis-C10

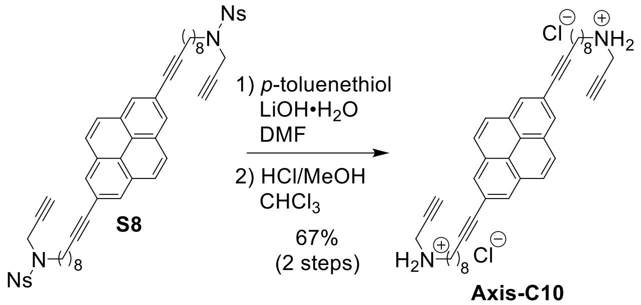

To a DMF (3 mL) solution of **S8** (191 mg, 201 μmol) and LiOH·H_2O (89.1 mg, 2.12 mmol) was slowly added *p*-toluenethiol (219 mg, 1.76 mmol) in DMF (2 mL) at 0 °C. The mixture was allowed to warm to room temperature, and additionally stirred for 1 h. To the resultant mixture was added CH_2Cl_2 (10 mL) and the resultant precipitate was removed by filtration through a celite bed. The filtrate was concentrated by a rotary evaporator and subjected to silica gel column chromatography (eluent: CH_2Cl_2/MeOH = 1:0 to 5:1) to give 2,7-bis{10-[*N*-(2-propyn-1-yl)amino]decyn-1-yl}pyrene (93.1 mg) as a white solid. To a $CHCl_3$ (5 mL) solution of the product (93.1 mg) was added 5% HCl/MeOH (1 mL) at room temperature. After stirring for 10 min, to the resultant solution was added Et_2O (10 mL) to precipitate a white solid, collected by filtration, and dried under vacuum to give **Axis-C10** (88.2 mg, 67%; 2 steps) as a white solid. Decomposition point 187.7 °C; IR (KBr) ν_{max} = 3428, 3238, 2929, 2853, 2781, 2727, 2602, 2422, 2131, 1603 cm^{-1}; ^{1}H NMR (CD_3OD, 500 MHz) δ = 8.16 (s, 4H), 8.03 (s, 4H), 3.92 (d, J = 2.6 Hz, 4H), 3.23 (t, J = 2.6 Hz, 2H), 3.07 (t, J = 7.9 Hz, 4H), 2.54 (t, J = 7.0 Hz, 4H), 1.73−1.68 (m, 8H), 1.59−1.55 (m, 4H), 1.44 (bs, 12H); ^{13}C NMR (CD_3OD, 125 MHz) δ = 132.4, 129.0, 128.5, 124.5, 123.4, 91.8, 82.0, 79.1, 74.6, 48.0, 37.3, 30.1, 30.0, 29.93, 29.90, 27.5, 27.0, 20.1; HRMS (ESI-TOF): calcd for $C_{42}H_{50}N_2$ [M−2Cl]$^{2+}$ 291.1982; found 291.1980.

Rota-C10

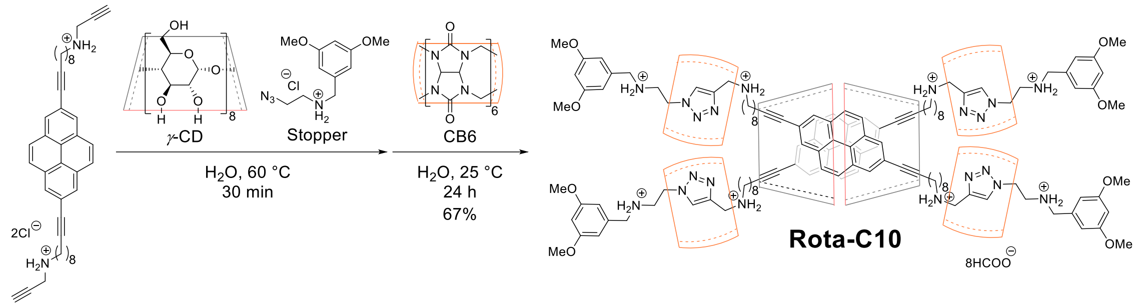

Axis-C10

A H_2O (500 μL) solution of **Axis-C10** (1.30 mg, 1.98 μmol), 2-azido-*N*-[(3,5-dimethoxyphenyl)methyl]-ethylammonium chloride (Stopper) (1.15 mg, 4.21 μmol), and *γ*-CD (2.73 mg, 2.10 μmol) was stirred for 30 min at 60 °C. To the solution was added cucurbit[6]uril (4.83 mg, 4.85 μmol), and the mixture was stirred for 24 h at 25 °C. The resulting solution was filtered by a membrane filter (PTFE, pore size: 0.22 μm), and purified by reverse-phase HPLC to give **Rota-C10** (6.05 mg, 67%) as a white solid. Decomposition point 286.0 °C; IR (KBr) ν_{max} = 3410, 2931, 2106, 1737, 1600, 1475 cm^{-1}; ^1H NMR (500 MHz, D_2O) δ = 8.43 (s, 8H), 7.62 (s, 4H), 7.50 (m, 12H), 6.98 (d, J = 1.5 Hz, 8H), 6.79 (t, J = 1.5 Hz, 4H), 6.56 (s, 4H), 5.78−5.74 (m, 48H), 5.54 (s, 48H), 4.98 (s, 16H), 4.54 (s, 8H), 4.33–4.27 (m, 56H), 4.21 (s, 8H), 4.12 (d, J = 10.0 Hz, 16H), 3.95 (d, J = 10.0 Hz, 16H), 3.89−3.81 (m, 48H), 3.62−3.56 (bs, 16H), 3.48−3.37 (m, 40H), 2.72−2.62 (bs, 8H), 2.21−2.15 (bs, 8H), 1.97−1.85 (m, 8H), 1.79−1.63 (bs, 32H); ^{13}C NMR (125 MHz, D_2O) δ = 171.1, 160.7, 156.6, 156.3, 139.1, 134.4, 129.4, 120.2, 107.9, 103.7, 101.3, 81.4, 72.8, 72.7, 72.4, 70.3, 68.6, 60.1, 55.7, 55.6, 51.6, 51.4, 49.2, 46.1, 45.5, 42.2, 29.0, 26.7, 25.9, 20.1 ppm, the ^{13}C NMR signals may be imperfect because of the poor signal-noise ratio; HRMS (ESI-TOF): calcd for $C_{368}H_{472}N_{116}O_{136}$ $[M−8HCOO]^{8+}$ 1086.9209, Found 1086.9266.

Reference compound **S9**

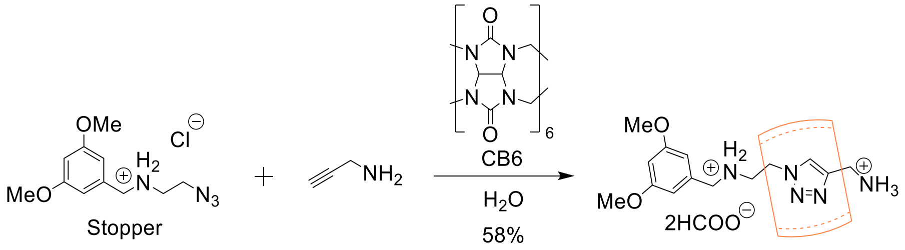

A H_2O (1 mL) solution of 2-azido-*N*-[(3,5-dimethoxyphenyl)methyl]-ethylammonium chloride (Stopper) (1.07 mg, 3.91 µmol), propargyl amine (0.834 mg, 15.1 µmol), and cucurbit[6]uril (5.70 mg, 5.72 µmol) was stirred for 32 h at 60 °C. The resulting solution was filtered by a membrane filter (PTFE, pore size: 0.22 µm), and purified by reverse-phase HPLC to give **S9** (3.16 mg, 58%) as a white solid. Decomposition point 242.4 °C; IR (KBr) ν_{max} = 3440, 2060, 1736, 1597, 1475 cm^{-1}; ^1H NMR (500 MHz, D_2O) δ = 8.40 (s, 2H), 7.00 (d, J = 2.3 Hz, 2H), 6.69–6.68 (m, 1H), 6.55 (s, 1H), 5.78–5.70 (m, 12H), 5.52 (s, 12H), 4.55 (s, 2H), 4.30–4.26 (m, 12H), 4.20–4.18 (m, 4H), 3.88 (s, 6H), 3.81 (t, J = 6.4 Hz, 2H); ^{13}C NMR (125 MHz, D_2O) δ = 161.3, 157.1, 156.9, 140.6, 134.9, 120.6, 108.5, 101.9, 70.9, 56.3, 52.1, 52.0, 51.9, 46.4, 45.9, 35.1; HRMS (ESI-TOF): calcd for $C_{50}H_{59}N_{29}O_{14}$ $[M-2HCOO]^{2+}$ 644.7393, Found 644.7411.

3. References

1 K. Dhbaibi, L. Favereau, M. Srebro-Hooper, C. Quinton, N. Vanthuyne, L. Arrico, T. Roisnel, B. Jamoussi, C. Poriel, C. Cabanetos, J. Autschbach and J. Crassous, *Chem. Sci.*, 2020, **11**, 567–576.

2 C. Schaack, L. Arrico, E. Sidler, M. Górecki, L. Di Bari and F. Diederich, *Chem. Eur. J.*, 2019, **25**, 8003–8007.

3 Y. Yu, C. Wang, F.-F. Hung, L. Jiang, C.-M. Che and J. Liu, *Angew. Chem. Int. Ed.*, 2025, **64**, e202501645.

4. C. Maeda, Y. Daigen, S. Michishita and T. Ema, *Org. Lett.*, 2025, **27**, 6648–6653.

5 C. Bræstrup, X. Xiao, F. García-González, T. Brock-Nannestad, D. Aranda, S. T. Bao, D. Cavlovic, H. Jiang, F. Ng, C. Nuckolls, F. Santoro and M. Pittelkow, *Adv. Opt. Mater.*, 2025, **13**, 2403061.

6 X. Xiao, S. K. Pedersen, D. Aranda, J. Yang, R. A. Wiscons, M. Pittelkow, M. L. Steigerwald, F. Santoro, N. J. Schuster and C. Nuckolls, *J. Am. Chem. Soc.*, 2021, **143**, 983–991.

7 Y.-J. Shen, N.-T. Yao, L.-N. Diao, Y. Yang, X.-L. Chen and H.-Y. Gong, *Angew. Chem. Int. Ed.*, 2023, **62**, e202300840.

8 W. Niu, Y. Fu, Z.-L. Qiu, C. J. Schürmann, S. Obermann, F. Liu, A. A. Popov, H. Komber, J. Ma and X. Feng, *J. Am. Chem. Soc.*, 2023, **145**, 26824–26832.

9 M. Hasegawa, C. Hasegawa, Y. Nagaya, K. Tsubaki and Y. Mazaki, *Chem. Eur. J.*, 2022, **28**, e202202218.

10 W. Zheng, T. Ikai, K. Oki and E. Yashima, *Nat. Sci.*, 2022, **2**, e20210047.

11 S. Kobayashi, R. Nakazono, R. Okada, M. P. Schramm and T. Amaya, *Bull. Chem. Soc. Jpn.*, 2024, **97**, uoae098.

12 S. Guo, L. Liu, X. Li, G. Liu, Y. Fan, J. He, Z. Lian, H. Yang, X. Chen and H. Jiang, *Small*, 2024, **20**, 2308429.

13 K. Matsumura, R. Inoue and Y. Morisaki, *Adv. Funct. Mater.*, 2024, **34**, 2310566.

14 Y. Fujita, R. Inoue and Y. Morisaki, *Asian. J. Org. Chem.*, 2025, **14**, e202500155.

15 A. Morisaki, R. Inoue and Y. Morisaki, *Chem. Eur. J.*, 2023, **29**, e202203533.

16 Y. Morisaki, K. Inoshita and Y. Chujo, *Chem. Eur. J.*, 2014, **20**, 8386–8390.

17 Z. Ma, Q. Sun, J. Zhou, Y. Liu, Z. Shuai, Z. Wang and W. Jiang, *ACS Mater. Lett.*, 2023, **5**, 450–457.

18 Y. Liu, Z. Ma, Z. Wang and W. Jiang, *J. Am. Chem. Soc.*, 2022, **144**, 11397–11404.

19 Y. Tanioka, M. Takase, M. Hamasu, S. Hata, K. Hashimoto, S. Mori, Y. Ishibashi, Y. Nukumi, M. Higashi, H. Sato, T. Okujima and H. Uno, *Angew. Chem. Int. Ed.*, 2025, **64**, e202509190.

20 M. Saikawa, T. Nakamura, J. Uchida, M. Yamamura and T. Nabeshima, *Chem. Commun.*, 2016, **52**, 10727–10730.

21 M. J. Álvaro-Martins, C. Billiaux, P. Godard, R. Oda, G. Raffy and D. M. Bassani, *Chem. Commun.*, 2023, **59**, 7963–7966.

22 K. Takaishi, R. Takehana and T. Ema, *Chem. Commun.*, 2018, **54**, 1449–1452.

23 D. Zheng, S. Guo, L. Zheng, Q. Xu, Y. Wang and H. Jiang, *Chem. Commun.*, 2021, **57**, 12016–12019.

24 H. Shigemitsu, K. Kawakami, Y. Nagata, R. Kajiwara, S. Yamada, T. Mori and T. Kida, *Angew. Chem. Int. Ed.*, 2022, **61**, e202114700.

25 C. Tu, W. Wu, W. Liang, D. Zhang, W. Xu, S. Wan, W. Lu and C. Yang, *Angew. Chem. Int. Ed.*, 2022, **61**, e202203541.

26 A. Homberg, E. Brun, F. Zinna, S. Pascal, M. Górecki, L. Monnier, C. Besnard, G. Pescitelli, L. D. Bari and J. Lacour, *Chem. Sci.*, 2018, **9**, 7043–7052.

27 J.-L. Song, C. Chen, X. Li, Y. Jiang, Z. Peng, X.-Q. Wang, H.-B. Yang and W. Wang, *Nat. Commun.*, 2024, **15**, 10531.

28 M. Iwamura, Y. Kimura, R. Miyamoto and K. Nozaki, *Inorg. Chem.*, 2012, **51**, 4094–4098.

29 A. G. Crawford, Z. Liu, I. A. I. Mkhalid, M.-H. Thibault, N. Schwarz, G. Alcaraz, A. Steffen, J. C. Collings, A. S. Batsanov, J. A. K. Howard and T. B. Marder, *Chem. Eur. J.*, 2012, **18**, 5022–5035.

30 Ò. Torres, T. Parella, M. Solà, A. Roglans and A. Pla-Quintana, *Chem. Eur. J.*, 2015, **21**, 16240–16245.

31 Y. Ohishi, K. Nishioki, Y. Miyaoka, K. Serizawa, S. Sugawara, K. Hayashi, D. Inoue, M. Iwamura, S. Yokoyama, J. Chiba and M. Inouye, *Adv. Opt. Mater.*, 2024, **12**, 2301457.

4. Calculated energies and geometric coordinates

[4]Rotaxane 2 (1707.213 kJ/mol)

C	5.5162	2.001	2.4462
C	6.1274	2.6002	3.5625
C	6.4442	1.8585	4.687
C	6.1643	0.4983	4.7678
C	5.2305	0.5933	2.4854
C	5.5491	-0.1816	3.665
C	5.2465	-1.566	3.6466
C	4.6882	-2.1759	2.536
C	4.3417	0.7469	0.1624
C	3.7814	0.0653	-0.9668
C	3.5553	-1.306	-0.9152
C	3.8544	-2.0491	0.2166
C	4.3885	-1.4436	1.3724
C	4.6485	-0.0301	1.345
C	4.6269	2.135	0.1911
C	5.1957	2.7363	1.298
H	6.3617	3.6554	3.531
H	5.4796	-2.1702	4.5113
H	4.4934	-3.2392	2.5537
H	3.6954	-3.117	0.2106
H	4.4105	2.7449	-0.6738
H	5.4015	3.7954	1.2921
C	9.1843	-0.2181	2.8446
C	9.7008	0.8805	3.6028
C	9.6718	2.1687	3.0821
C	9.1937	2.4312	1.8091
C	8.6858	0.0546	1.5147
C	8.7042	1.3951	0.9916
C	8.2166	1.6372	-0.3021
C	7.7248	0.6098	-1.0844
C	8.0912	-2.3292	1.2573
C	7.5283	-3.3499	0.4653
C	7.059	-3.0857	-0.8121
C	7.1364	-1.8173	-1.3743
C	7.6687	-0.726	-0.6169
C	8.1559	-0.9977	0.717
C	8.5699	-2.5683	2.5565
C	9.1145	-1.552	3.3201
H	9.1894	3.4455	1.4338
H	8.2102	2.6505	-0.6784
H	7.34	0.8403	-2.0648
H	7.4602	-4.3516	0.8672
H	8.5022	-3.5665	2.9655
H	9.4664	-1.7814	4.3159
C	7.3041	6.6877	5.8102
C	7.5864	6.9865	4.3257
C	8.7346	6.1106	3.7698
C	9.9636	6.2067	4.695
C	9.5458	5.8928	6.1546
C	10.7195	6.0443	7.1483
O	6.3912	6.9011	3.5702
O	9.0622	6.4738	2.4271
O	10.9427	5.2694	4.2286
O	8.4776	6.7633	6.5806
O	10.3247	5.7301	8.4768
C	3.2888	3.7288	7.7424
C	3.0345	4.6788	6.5516
C	4.329	4.944	5.7445
C	5.4998	5.3137	6.6843
C	5.6286	4.2389	7.7892
C	6.7682	4.5447	8.7872
O	1.9694	4.2123	5.7303
O	4.1006	5.9284	4.7364
O	6.7193	5.4133	5.928
O	4.3873	4.1465	8.5124
O	6.9872	3.4623	9.6841
C	2.0571	-1.459	8.0615
C	1.2683	-0.6091	7.0442
C	2.0877	0.6076	6.5515
C	2.6398	1.3919	7.7555
C	3.3986	0.4302	8.7025
C	3.9595	1.1144	9.9712
O	0.7714	-1.4127	5.9857
O	1.2982	1.4482	5.7099
O	3.5011	2.4277	7.2587
O	2.5699	-0.6763	9.1087
O	2.9481	1.7421	10.753
C	4.5928	-6.0417	6.9808
C	3.3277	-5.6994	6.1694
C	3.0957	-4.1715	6.1018
C	3.1503	-3.5584	7.5158
C	4.4434	-4.0047	8.2414
C	4.4877	-3.5076	9.7043
O	3.3461	-6.3072	4.8869
O	1.8622	-3.8847	5.4452
O	3.1023	-2.1267	7.4028
O	4.5547	-5.4423	8.2514
O	5.7091	-3.8617	10.3378
C	9.3579	-7.1456	4.8033
C	8.0973	-7.4804	3.9912
C	6.9129	-6.5719	4.3924
C	6.7127	-6.6023	5.9216
C	8.0537	-6.3256	6.652
C	7.9138	-6.5067	8.182
O	8.3675	-7.4627	2.5996
O	5.7328	-6.965	3.6945
O	5.7304	-5.6141	6.2752
O	9.0949	-7.2075	6.1815

O	9.1102	-6.1784	8.8745
C	13.3592	-4.0315	3.092
C	12.6497	-4.9569	2.0812
C	11.2055	-5.2873	2.5276
C	11.1761	-5.7448	4.0028
C	11.9318	-4.7244	4.8857
C	12.0275	-5.1806	6.3571
O	12.6957	-4.4407	0.7587
O	10.6229	-6.2478	1.6468
O	9.8144	-5.8655	4.4445
O	13.273	-4.5418	4.3961
C	14.3339	1.1473	2.2865
C	13.7974	0.3515	1.0769
C	12.9232	-0.8491	1.5133
C	13.6623	-1.6833	2.5759
C	14.0933	-0.752	3.735
C	14.8393	-1.4685	4.8824
O	13.1608	1.1977	0.1262
O	12.5793	-1.6521	0.3896
O	12.8024	-2.7418	3.031
O	14.9343	0.312	3.2454
C	12.264	5.716	4.0645
C	12.664	5.5737	2.5841
C	12.6951	4.0849	2.1647
C	13.513	3.2414	3.1665
C	13.1073	3.5494	4.6294
C	14.0443	2.8567	5.6443
O	11.8265	6.3442	1.7316
O	13.1556	3.9341	0.8226
O	13.2822	1.8564	2.8788
O	13.1325	4.9697	4.8783
O	13.6529	3.1306	6.982
H	6.6849	7.4855	6.2332
H	7.8994	8.0324	4.2774
H	8.4027	5.07	3.7422
H	10.343	7.2303	4.642
H	9.2072	4.8546	6.2017
H	11.534	5.3742	6.8726
H	11.1198	7.0595	7.1165
H	6.64	6.7995	2.6295
H	10.0183	6.3132	2.2966
H	9.7174	6.4235	8.7755
H	2.469	3.7888	8.4656
H	2.6995	5.6251	6.983
H	4.5709	4.018	5.2233
H	5.264	6.258	7.1805
H	5.8572	3.2781	7.3241
H	7.6993	4.7223	8.2487
H	6.553	5.4571	9.3466
H	2.0622	4.6473	4.8625
H	4.9729	6.2474	4.4187

H	7.7015	3.708	10.2907
H	1.3766	-2.1332	8.5913
H	0.3897	-0.2362	7.5764
H	2.9241	0.245	5.948
H	1.7841	1.8077	8.2927
H	4.2567	0.0311	8.1587
H	4.4846	0.3795	10.5843
H	4.7073	1.8576	9.6929
H	0.5039	-0.8139	5.2607
H	1.6664	2.354	5.7635
H	3.3688	2.1529	11.5234
H	4.6148	-7.1078	7.2286
H	2.4901	-6.152	6.7057
H	3.8884	-3.7331	5.4915
H	2.2986	-3.9471	8.0789
H	5.3012	-3.5778	7.7161
H	4.3935	-2.4218	9.7375
H	3.648	-3.9124	10.2726
H	2.6487	-5.8785	4.3541
H	1.5816	-2.9813	5.7067
H	5.7082	-4.8217	10.4657
H	10.1117	-7.9282	4.6706
H	7.8402	-8.5142	4.2353
H	7.1407	-5.5491	4.0824
H	6.3816	-7.6086	6.1892
H	8.347	-5.2901	6.4629
H	7.1174	-5.8682	8.5658
H	7.6298	-7.5344	8.4165
H	7.5083	-7.5084	2.1355
H	4.9568	-6.6542	4.2085
H	8.9902	-6.3882	9.812
H	14.4431	-4.0322	2.9348
H	13.2254	-5.8855	2.0662
H	10.6204	-4.3731	2.4269
H	11.707	-6.6969	4.0744
H	11.3984	-3.7704	4.8667
H	11.032	-5.2886	6.7904
H	12.5083	-6.1584	6.4242
H	12.0809	-4.9758	0.2237
H	9.8393	-6.6368	2.0927
H	15.1583	1.7914	1.9623
H	14.6805	-0.0444	0.5692
H	11.9896	-0.4763	1.9408
H	14.5579	-2.0902	2.0998
H	13.1925	-0.3198	4.1785
H	12.7509	0.6168	-0.5433
H	12.4945	-2.5763	0.7008
H	12.4182	6.7374	4.4275
H	13.6713	5.9878	2.4934
H	11.6626	3.7403	2.1617
H	14.5688	3.5052	3.0661

H	12.1005	3.1662	4.8044
H	14.0288	1.776	5.4947
H	15.076	3.1809	5.4957
H	12.0217	6.0491	0.8275
H	13.2011	2.9748	0.6277
H	13.7675	4.0799	7.1357
H	14.1922	-2.2202	5.3364
H	15.0702	-0.7525	5.6732
O	16.0422	-2.0881	4.4439
H	16.6588	-1.3896	4.1773
C	10.1696	2.0846	-4.9809
C	10.8839	3.1937	-4.1735
C	9.9497	3.7917	-3.0959
C	8.6021	4.1941	-3.7294
C	8.0038	2.9759	-4.4726
C	6.6152	3.2263	-5.1111
O	12.1147	2.7552	-3.6109
O	10.5749	4.8672	-2.3933
O	7.7315	4.6651	-2.695
O	8.9235	2.5104	-5.4782
O	6.5536	4.3967	-5.9172
C	11.1882	-3.0698	-4.2116
C	12.0009	-2.1302	-3.3073
C	11.2706	-0.7811	-3.0884
C	10.8431	-0.1742	-4.4421
C	10.0982	-1.2347	-5.2947
C	9.7004	-0.7603	-6.7121
O	12.3443	-2.8031	-2.106
O	12.0857	0.1362	-2.3579
O	9.9957	0.9519	-4.1738
O	10.8664	-2.4468	-5.426
O	10.8149	-0.3131	-7.4759
C	7.8041	-6.9103	-2.6925
C	9.1695	-6.7826	-1.9809
C	9.7425	-5.3478	-2.0875
C	9.6739	-4.8372	-3.5407
C	8.2481	-5.0267	-4.1001
C	8.1298	-4.5766	-5.5717
O	9.0869	-7.24	-0.6369
O	11.0644	-5.2655	-1.5536
O	10.0183	-3.4471	-3.5378
O	7.8625	-6.4112	-4.0036
O	6.7784	-4.4639	-5.9877
C	2.9002	-6.8541	-0.5552
C	4.053	-7.4508	0.2808
C	5.4237	-6.8537	-0.1218
C	5.6039	-6.885	-1.6507
C	4.387	-6.2029	-2.3179
C	4.4854	-6.1815	-3.8569
O	3.7855	-7.3371	1.6718
O	6.4956	-7.5274	0.5338
O	6.8339	-6.2131	-1.9565
O	3.1851	-6.8982	-1.9305
O	3.442	-5.4211	-4.4474
C	-0.5416	-3.0507	0.9646
C	0.0044	-3.9967	2.0529
C	1.3775	-4.5885	1.6553
C	1.3348	-5.1611	0.2226
C	0.7488	-4.1111	-0.754
C	0.6124	-4.6529	-2.195
O	0.026	-3.3595	3.3227
O	1.8074	-5.57	2.5979
O	2.6728	-5.5279	-0.1503
O	-0.5442	-3.6742	-0.2944
O	0.22	-3.6381	-3.1107
C	-0.5383	2.3006	0.9659
C	-0.5521	1.5593	2.3224
C	0.112	0.1628	2.2352
C	-0.4572	-0.6229	1.0399
C	-0.3014	0.2309	-0.2393
C	-0.8146	-0.4669	-1.5195
O	-0.0111	2.3659	3.361
O	-0.064	-0.5528	3.4559
O	0.2356	-1.8781	0.9329
O	-0.9824	1.4916	-0.0938
C	2.7354	6.2987	-0.3795
C	2.4501	6.1245	1.1264
C	2.0188	4.6756	1.4615
C	0.928	4.1743	0.4927
C	1.3254	4.4605	-0.9773
C	0.2007	4.0951	-1.9719
O	3.5464	6.5589	1.9194
O	1.6098	4.5726	2.826
O	0.7582	2.7632	0.7009
O	1.6462	5.8557	-1.1474
C	7.5953	6.0604	-2.5999
C	7.5432	6.4654	-1.1197
C	6.2505	5.9437	-0.4542
C	4.9987	6.3033	-1.2823
C	5.2146	5.9063	-2.7625
C	4.0591	6.3385	-3.6892
O	8.7294	6.0699	-0.4385
O	6.1353	6.4285	0.8785
O	3.8921	5.5801	-0.7211
O	6.4364	6.4793	-3.2701
O	4.2368	5.8268	-5.0064
H	10.7128	1.8559	-5.9036
H	11.1349	3.9796	-4.8902
H	9.7829	3.0081	-2.3541
H	8.766	4.9734	-4.4786
H	7.8547	2.1714	-3.7523
H	6.3206	2.3617	-5.708

H	5.8625	3.306	-4.3275
H	12.3887	3.4531	-2.994
H	9.9102	5.2628	-1.7959
H	5.8023	4.9413	-5.6088
H	11.8032	-3.9228	-4.5152
H	12.9409	-1.9349	-3.829
H	10.3705	-0.9536	-2.4923
H	11.7501	0.1418	-4.9648
H	9.1632	-1.4698	-4.7809
H	9.2072	-1.5766	-7.2424
H	8.9645	0.0431	-6.6472
H	12.4649	-2.1288	-1.4076
H	11.9133	1.0273	-2.7179
H	10.5172	-0.1144	-8.3756
H	7.5534	-7.9649	-2.8397
H	9.8495	-7.4631	-2.499
H	9.1268	-4.7064	-1.4575
H	10.3556	-5.4256	-4.1595
H	7.5664	-4.4067	-3.5155
H	8.5957	-3.6049	-5.721
H	8.6591	-5.2704	-6.2187
H	9.8338	-6.8457	-0.1495
H	11.4538	-4.4159	-1.8481
H	6.5073	-3.5364	-5.8587
H	2.0106	-7.4866	-0.4754
H	4.0691	-8.5204	0.0604
H	5.4508	-5.815	0.217
H	5.6299	-7.9283	-1.9714
H	4.3416	-5.1623	-1.987
H	5.4277	-5.7342	-4.1627
H	4.4811	-7.1923	-4.2609
H	4.6413	-7.3826	2.1407
H	7.3118	-7.3724	0.0147
H	3.5559	-5.4721	-5.4147
H	-1.6062	-2.8576	1.13
H	-0.7131	-4.8169	2.1337
H	2.1066	-3.7784	1.698
H	0.6679	-6.0264	0.2281
H	1.4255	-3.2571	-0.7944
H	1.568	-5.0489	-2.5352
H	-0.0979	-5.481	-2.2299
H	0.662	-3.8447	3.8828
H	2.4781	-6.1359	2.1609
H	0.2482	-4.0105	-4.0044
H	-1.2885	3.0977	0.9696
H	-1.6058	1.4152	2.5735
H	1.1882	0.2962	2.0955
H	-1.5249	-0.7718	1.2158
H	0.7623	0.4257	-0.3974
H	-0.6559	0.1809	-2.3837
H	0.2527	1.7692	4.0883
H	0.0066	-1.5102	3.2581
H	2.7978	7.3582	-0.6479
H	1.6182	6.7943	1.3573
H	2.8928	4.0366	1.3527
H	0.0146	4.731	0.7151
H	2.1939	3.8482	-1.2315
H	3.388	6.2417	2.8278
H	1.0585	3.7666	2.915
H	8.4022	6.6012	-3.107
H	7.5277	7.5579	-1.0997
H	6.3334	4.8567	-0.4013
H	4.8498	7.3841	-1.2226
H	5.2658	4.8178	-2.8016
H	3.1068	5.9704	-3.3065
H	3.9845	7.4273	-3.7202
H	8.5723	6.2168	0.5136
H	5.1874	6.4033	1.1282
H	3.5621	6.2284	-5.5752
H	-0.2255	-1.365	-1.7072
H	-0.7166	4.6352	-1.7296
H	-0.0305	3.0317	-1.9043
O	0.5775	4.38	-3.3118
H	0.617	5.343	-3.4073
O	12.7689	-4.2409	7.1208
H	13.6593	-4.2056	6.7433
O	-2.1913	-0.8228	-1.4439
H	-2.4145	-1.3371	-2.2341
H	6.9352	2.3616	5.5078
H	10.0272	2.9968	3.676
H	3.1544	-1.8188	-1.7791
H	6.6187	-3.8923	-1.3757
C	6.9286	-0.6523	7.0659
C	6.5496	-0.1755	6.0066
C	3.153	1.3738	-3.2269
C	3.4392	0.7614	-2.2082
C	10.7386	0.6363	6.063
C	10.2651	0.7189	4.9402
C	6.1546	-1.4699	-3.8475
C	6.6304	-1.6492	-2.7366
C	7.4121	-1.1387	8.3671
H	7.5058	-0.2691	9.0204
H	6.647	-1.7916	8.7841
O	8.6541	-1.8215	8.2473
C	9.1894	-2.2371	9.5008
H	9.3469	-1.3748	10.1517
H	8.4942	-2.9092	10.0078
C	10.525	-2.9576	9.2736
H	10.3491	-3.8558	8.682
H	11.1936	-2.3227	8.6912
O	11.1203	-3.2873	10.5279
C	12.3253	-4.0364	10.3904

H	12.1255	-4.9784	9.8755
H	13.0459	-3.484	9.7879
C	12.9434	-4.3274	11.7654
H	13.1602	-3.3903	12.2816
H	12.2293	-4.8836	12.3758
O	14.1399	-5.0801	11.5804
C	14.7742	-5.4726	12.7942
H	15.0812	-4.594	13.3644
H	14.083	-6.0436	13.4175
C	16.0017	-6.3374	12.4663
H	15.7047	-7.147	11.7965
H	16.745	-5.7356	11.9399
O	16.5529	-6.8691	13.6692
C	17.6713	-7.728	13.456
H	17.407	-8.537	12.772
H	18.4957	-7.1748	13.0023
C	18.1194	-8.3288	14.7971
H	18.3459	-7.5309	15.5062
H	17.2987	-8.9136	15.2177
O	19.2652	-9.1519	14.5943
C	19.6945	-9.8229	15.7749
H	20.0107	-9.108	16.5363
H	18.8906	-10.4303	16.1947
C	21.7469	-11.501	15.2999
C	20.8253	-10.7246	15.5038
C	24.8935	-14.2782	14.7263
C	23.6732	-14.7293	15.246
C	22.6423	-13.798	15.4192
C	22.8255	-12.4552	15.0871
C	24.0499	-12.0317	14.5697
C	25.1055	-12.9342	14.3903
H	25.6954	-14.9883	14.5813
H	21.6964	-14.1208	15.8318
H	24.193	-10.9882	14.3263
C	23.1222	-18.8583	16.3568
C	22.1102	-18.1591	15.682
C	22.2917	-16.8263	15.3217
C	23.4838	-16.1592	15.6226
C	24.4938	-16.8587	16.2918
C	24.3171	-18.1903	16.6573
H	21.1728	-18.637	15.4291
H	21.497	-16.3121	14.7991
H	25.4225	-16.3657	16.5444
H	25.1199	-18.6944	17.1799
C	28.9297	-11.6429	12.8744
C	27.7401	-11.1389	12.3306
C	26.5052	-11.5533	12.8234
C	26.4265	-12.4789	13.8692
C	27.6146	-12.9717	14.4196
C	28.8519	-12.5611	13.9315
H	27.7602	-10.4251	11.5174
H	25.6036	-11.1563	12.3782
H	27.5835	-13.6727	15.2422
H	29.7452	-12.9655	14.3885
C	30.2658	-11.1943	12.3228
O	30.3726	-10.3761	11.4113
O	31.3565	-11.7636	12.8928
H	32.1097	-11.3862	12.4292
C	22.942	-20.3063	16.7598
O	23.8083	-20.9496	17.3497
O	21.7507	-20.8624	16.427
H	21.799	-21.768	16.7486
C	11.289	0.6027	7.4252
H	11.4275	1.638	7.7413
H	10.5362	0.1533	8.0739
O	12.5121	-0.1246	7.4873
C	13.1056	-0.0788	8.7822
H	13.3825	0.9475	9.0311
H	12.3948	-0.4128	9.5411
C	14.3536	-0.9713	8.8329
H	14.1223	-1.9534	8.4177
H	15.1468	-0.5375	8.2216
O	14.7778	-1.0748	10.189
C	15.857	-1.9797	10.4
H	15.5537	-2.9982	10.1506
H	16.705	-1.7226	9.7623
C	16.2814	-1.9023	11.8743
H	16.6357	-0.8918	12.0889
H	15.4195	-2.0876	12.5181
O	17.3042	-2.8578	12.1424
C	17.8452	-2.75	13.4573
H	18.3111	-1.7722	13.5952
H	17.0547	-2.8444	14.2046
C	18.887	-3.8556	13.6777
H	18.385	-4.8232	13.6712
H	19.6038	-3.8518	12.8553
O	19.551	-3.6495	14.9244
C	20.4085	-4.7132	15.3438
H	20.7647	-4.4709	16.3456
H	19.8296	-5.6317	15.447
C	21.6265	-4.9332	14.4151
H	21.3194	-5.376	13.4659
H	22.061	-3.9642	14.167
O	22.6566	-5.7246	15.0083
C	22.3215	-7.0929	15.2291
H	21.5446	-7.1899	15.9879
H	21.9633	-7.5658	14.3128
C	24.4713	-8.4893	16.0525
C	23.489	-7.8572	15.6939
C	27.9149	-10.7126	17.115
C	26.6594	-11.1569	17.5539
C	25.5329	-10.4001	17.2068

C	25.6548	-9.2453	16.4345
C	26.9158	-8.8226	16.0164
C	28.066	-9.5475	16.3502
H	28.7916	-11.2972	17.3547
H	24.5521	-10.7192	17.5307
H	27.0047	-7.9208	15.4267
C	26.2796	-14.7962	19.8432
C	25.3211	-14.4522	18.8813
C	25.4459	-13.2754	18.1492
C	26.5255	-12.41	18.3548
C	27.4771	-12.7498	19.3231
C	27.3597	-13.9271	20.0585
H	24.4724	-15.0951	18.6862
H	24.6963	-13.0436	17.407
H	28.3178	-12.0976	19.5144
H	28.1204	-14.1519	20.7944
C	31.9617	-8.2279	15.048
C	30.8507	-8.1201	14.2009
C	29.5949	-8.5474	14.6237
C	29.4139	-9.0923	15.8996
C	30.5237	-9.1973	16.7453
C	31.7825	-8.7725	16.3281
H	30.9488	-7.7096	13.2041
H	28.759	-8.4641	13.943
H	30.4142	-9.6054	17.7405
H	32.6126	-8.8724	17.0151
C	26.1388	-16.085	20.6243
O	25.2124	-16.8748	20.4519
C	33.3225	-7.7626	14.5757
O	33.5176	-7.2945	13.4555
O	27.1098	-16.3224	21.5407
H	26.8748	-17.1649	21.9418
O	34.3302	-7.8945	15.4735
H	35.1118	-7.5578	15.0243
C	2.7804	2.176	-4.4041
H	1.6981	2.1073	-4.5237
H	3.0301	3.2115	-4.1644
O	3.4409	1.75	-5.595
C	3.2222	2.6521	-6.6818
H	2.1516	2.7791	-6.8562
H	3.6233	3.6344	-6.4314
C	3.8857	2.1694	-7.9827
H	3.8744	2.9894	-8.7037
H	4.9317	1.9227	-7.7925
O	3.203	1.0359	-8.517
C	3.7146	0.6174	-9.7828
H	3.7121	1.4538	-10.485
H	4.751	0.293	-9.6871
C	2.8634	-0.519	-10.3743
H	3.3842	-0.9273	-11.2414
H	2.756	-1.3275	-9.6488

O	1.581	-0.0224	-10.7545
C	0.7921	-0.9603	-11.4843
H	1.3695	-1.3933	-12.3044
H	0.4859	-1.782	-10.8346
C	-0.4416	-0.2497	-12.0659
H	-1.1512	-0.014	-11.2709
H	-0.1292	0.6957	-12.5148
O	-1.0555	-1.0865	-13.0431
C	-2.0906	-0.4461	-13.7888
H	-2.9188	-0.1761	-13.1311
H	-1.7197	0.4746	-14.2437
C	-2.5822	-1.388	-14.9007
H	-3.278	-0.8677	-15.5605
H	-1.7225	-1.6726	-15.511
O	-3.2337	-2.5296	-14.3348
C	-3.2772	-3.6894	-15.169
H	-4.0384	-4.3664	-14.7807
H	-3.5647	-3.4421	-16.1925
C	-0.9345	-5.0229	-15.105
C	-2.0017	-4.4325	-15.1793
C	2.7495	-7.0159	-14.4901
C	2.146	-6.3517	-13.4126
C	0.9318	-5.6943	-13.6482
C	0.3367	-5.7038	-14.9091
C	0.9662	-6.3602	-15.9664
C	2.1861	-7.0199	-15.7732
H	3.6909	-7.5225	-14.3377
H	0.4469	-5.1578	-12.8449
H	0.5064	-6.3609	-16.9449
C	4.0168	-6.0233	-9.5379
C	2.6222	-6.0856	-9.6486
C	2.0201	-6.228	-10.896
C	2.7907	-6.3062	-12.0634
C	4.1844	-6.3031	-11.937
C	4.7924	-6.1651	-10.6951
H	1.9909	-5.998	-8.7739
H	0.9409	-6.2549	-10.9469
H	4.8184	-6.374	-12.8074
H	5.8732	-6.1448	-10.6526
C	4.227	-8.9593	-19.0365
C	3.6373	-7.6982	-19.2103
C	2.9724	-7.0778	-18.1557
C	2.8798	-7.6969	-16.905
C	3.4639	-8.9559	-16.7339
C	4.1312	-9.5808	-17.7839
H	3.6889	-7.1824	-20.1602
H	2.5362	-6.1011	-18.3133
H	3.4067	-9.4563	-15.7772
H	4.5738	-10.553	-17.6098
C	4.6624	-5.7813	-8.1941
O	4.0191	-5.7012	-7.1496

C	4.9565	-9.6481	-20.1699	H	6.6997	-10.6683	-14.1268
O	5.487	-10.7512	-20.0516	C	6.7426	-9.3104	-11.1869
O	6.0033	-5.596	-8.2257	C	6.4165	-9.6149	-12.3245
H	6.2758	-5.3335	-7.3265	C	7.7856	-8.11	-7.26
O	4.9923	-8.9673	-21.3421	C	8.5774	-7.6873	-8.336
H	5.4829	-9.5336	-21.9459	C	8.2241	-8.1005	-9.6255
C	5.5142	-1.2128	-5.146	C	7.1061	-8.9072	-9.8364
H	4.5002	-1.614	-5.0917	C	6.3268	-9.3073	-8.7505
H	5.4435	-0.1307	-5.268	C	6.6494	-8.9091	-7.4468
O	6.245	-1.7981	-6.2223	H	8.0346	-7.7749	-6.2632
C	5.5895	-1.6306	-7.48	H	8.8214	-7.7832	-10.4689
H	4.6358	-2.1626	-7.4776	H	5.4574	-9.927	-8.9202
H	5.3708	-0.575	-7.6432	C	11.8983	-5.0289	-7.6644
C	6.4669	-2.1428	-8.6361	C	11.7119	-6.1552	-6.8484
H	7.3012	-1.4582	-8.796	C	10.6461	-7.023	-7.0753
H	6.8927	-3.1127	-8.3779	C	9.7421	-6.7864	-8.1164
O	5.6823	-2.234	-9.8234	C	9.9292	-5.6642	-8.9297
C	6.369	-2.8241	-10.9262	C	10.9937	-4.7951	-8.7088
H	7.259	-2.2426	-11.1738	H	12.3916	-6.3756	-6.0359
H	6.7	-3.8323	-10.6704	H	10.5225	-7.8848	-6.4342
C	5.4329	-2.8793	-12.1456	H	9.2355	-5.4528	-9.7317
H	4.4856	-3.3367	-11.8528	H	11.1048	-3.936	-9.3576
H	5.2137	-1.865	-12.4834	C	4.1629	-9.9963	-4.0959
O	6.0392	-3.62	-13.2027	C	3.5897	-9.6385	-5.325
C	5.1978	-3.7762	-14.3471	C	4.3966	-9.2925	-6.4056
H	4.2394	-4.2135	-14.061	C	5.7912	-9.2995	-6.291
H	4.9856	-2.8005	-14.7878	C	6.3614	-9.6676	-5.0677
C	5.867	-4.6805	-15.3986	C	5.5598	-10.0114	-3.9821
H	5.2026	-4.7865	-16.2585	H	2.5158	-9.6082	-5.454
H	6.7885	-4.2139	-15.7512	H	3.9269	-9.0015	-7.3349
O	6.1468	-5.9593	-14.836	H	7.4362	-9.6902	-4.9536
C	6.7307	-6.8882	-15.7451	H	6.038	-10.2889	-3.0517
H	6.0476	-7.0858	-16.5736	C	13.0617	-4.0855	-7.4477
H	7.65	-6.4783	-16.1673	O	13.3296	-3.1641	-8.2154
C	7.0487	-8.1999	-15.0066	C	3.3015	-10.338	-2.9003
H	7.5725	-8.8843	-15.6758	O	3.7633	-10.5205	-1.7762
H	7.7217	-7.981	-14.1756	O	13.8082	-4.3236	-6.3411
O	5.841	-8.8095	-14.5446	H	14.5128	-3.6696	-6.3685
C	5.9978	-9.9498	-13.7001	O	1.9715	-10.4342	-3.145
H	5.0347	-10.4552	-13.6262	H	1.5715	-10.6732	-2.3038

Rota-C6 (−1893.927 kJ/mol)

C	1.9078	1.5196	-0.2813	C	1.3737	-0.8211	-3.7997
C	2.0412	2.3533	0.8524	C	1.5742	-0.2524	-5.0527
C	2.3966	3.6927	0.7256	C	1.9933	1.0679	-5.1718
C	2.6599	4.2416	-0.5243	C	2.218	1.8748	-4.0356
C	2.1622	2.0899	-1.5764	C	1.9952	1.2955	-2.7393
C	2.5522	3.4688	-1.7026	C	1.3468	-0.6065	-1.3321
C	2.7841	3.998	-2.9883	C	1.5051	0.1715	-0.196
C	2.6218	3.2218	-4.1243	C	1.3107	-1.0363	-6.2495
C	1.5729	-0.0741	-2.6163	C	1.0268	-1.6857	-7.2438

C	0.612	-2.4699	-8.4189
C	2.4486	4.5324	1.9128
C	2.4067	5.1858	2.9435
C	2.2545	5.8826	4.2335
H	1.825	1.9391	1.8275
H	2.9276	5.2828	-0.6119
H	3.0746	5.0335	-3.0926
H	2.7842	3.6631	-5.0975
H	1.0265	-1.8405	-3.7161
H	2.1212	1.5109	-6.1448
H	1.0148	-1.6311	-1.2391
H	1.2782	-0.2496	0.7717
C	-1.3459	2.8248	-2.1236
C	-1.3765	2.7554	-0.715
C	-1.7478	1.586	-0.0625
C	-2.1447	0.4613	-0.7794
C	-1.7369	1.6604	-2.872
C	-2.1479	0.4611	-2.1933
C	-2.5003	-0.6693	-2.9578
C	-2.4729	-0.6374	-4.3438
C	-1.229	2.8691	-4.9637
C	-1.1644	2.8486	-6.3738
C	-1.5698	1.735	-7.1002
C	-2.0159	0.5894	-6.4491
C	-2.074	0.5219	-5.0386
C	-1.6883	1.6862	-4.2885
C	-0.848	3.9885	-4.1981
C	-0.9129	3.9705	-2.8159
C	-1.5017	1.7517	-8.5536
C	-1.3936	1.6677	-9.7672
C	-1.1602	1.4195	-11.2008
C	-1.6289	1.5193	1.3828
C	-1.3933	1.4443	2.577
C	-1.0095	1.297	3.986
H	-1.0504	3.6157	-0.147
H	-2.4166	-0.4476	-0.2615
H	-2.7834	-1.5795	-2.448
H	-2.7365	-1.5221	-4.9056
H	-0.7892	3.7219	-6.8832
H	-2.3052	-0.2806	-7.0202
H	-0.4955	4.8787	-4.6965
H	-0.5935	4.8369	-2.2553
C	5.9242	7.4001	-0.8275
C	6.5764	6.6881	-2.0272
C	6.361	5.1594	-1.9313
C	6.818	4.6387	-0.5543
C	6.2086	5.4909	0.5887
C	6.7018	5.0973	2.0019
O	6.1267	7.207	-3.2711
O	6.9968	4.4796	-3.013
O	6.4002	3.2746	-0.4395

O	6.4454	6.8988	0.3782
O	8.1087	4.917	2.0976
C	0.9183	9.2583	-0.9538
C	1.6183	9.099	-2.3077
C	2.7333	8.0294	-2.2285
C	3.6792	8.3257	-1.0402
C	2.8559	8.5568	0.2555
C	3.7159	8.9946	1.4591
O	0.648	8.8561	-3.3118
O	3.4377	7.9203	-3.4662
O	4.5376	7.1917	-0.8746
O	1.8499	9.5657	0.0461
O	2.9443	9.1055	2.6506
C	-3.6449	7.177	0.8233
C	-3.5341	7.7256	-0.6252
C	-2.0898	7.6531	-1.1783
C	-1.0926	8.1978	-0.1377
C	-1.2861	7.4175	1.1801
C	-0.252	7.7487	2.2832
O	-4.4723	7.1173	-1.5055
O	-1.9835	8.3195	-2.4378
O	0.2402	8.0647	-0.646
O	-2.6195	7.6456	1.6666
O	-0.0525	9.1367	2.5164
C	-6.1552	2.7213	2.3204
C	-6.4324	3.2095	0.8816
C	-5.2513	4.0306	0.306
C	-4.8015	5.1044	1.3188
C	-4.5307	4.4439	2.6962
C	-4.0911	5.4565	3.7812
O	-6.8297	2.1501	0.0245
O	-5.616	4.6083	-0.9499
O	-3.6385	5.7723	0.801
O	-5.7276	3.7758	3.1429
O	-3.8079	4.8104	5.0138
C	-4.5414	-2.2331	3.5391
C	-5.3852	-2.0565	2.2663
C	-5.2369	-0.6232	1.7091
C	-5.5565	0.4122	2.8039
C	-4.7889	0.0876	4.1144
C	-5.2538	0.9867	5.2815
O	-5.1138	-3.0577	1.3024
O	-6.0645	-0.4398	0.5639
O	-5.1882	1.705	2.297
O	-4.9456	-1.296	4.5034
O	-4.501	0.7484	6.4581
C	0.4969	-4.0747	3.2799
C	-0.5778	-4.6959	2.3799
C	-1.7624	-3.7246	2.1582
C	-2.2733	-3.1453	3.5056
C	-1.0718	-2.619	4.3365

C	-1.4429	-2.1011	5.7487
O	0.0032	-5.1676	1.1722
O	-2.7767	-4.3884	1.4064
O	-3.179	-2.0626	3.2336
O	-0.0681	-3.6407	4.4869
C	5.1269	-1.7862	1.9809
C	4.6453	-3.0621	1.2392
C	3.1026	-3.2002	1.2436
C	2.5378	-2.9376	2.6539
C	3.0442	-1.5589	3.1349
C	2.4344	-1.0655	4.4695
O	5.1798	-3.1575	-0.0761
O	2.7001	-4.4661	0.7198
O	1.1061	-3.0012	2.603
O	4.4795	-1.5932	3.2167
C	7.4041	2.3009	-0.5286
C	6.9397	1.1782	-1.4719
C	5.7784	0.36	-0.8553
C	6.1093	-0.0612	0.5939
C	6.579	1.1724	1.4077
C	7.03	0.8421	2.8467
O	6.6605	1.6852	-2.7713
O	5.4949	-0.7839	-1.6547
O	4.9368	-0.6584	1.1687
O	7.6957	1.7989	0.7481
O	7.3783	2.0269	3.5489
H	6.1974	8.459	-0.7835
H	7.6471	6.8995	-1.9715
H	5.2916	4.9755	-2.0464
H	7.904	4.7367	-0.487
H	5.1295	5.3295	0.5981
H	6.3977	5.8619	2.7148
H	6.2141	4.1748	2.3184
H	6.424	6.5729	-3.9441
H	6.8938	3.5165	-2.8643
H	8.4087	5.2654	2.9611
H	0.2645	10.1367	-0.9711
H	2.0733	10.0637	-2.5446
H	2.2599	7.0605	-2.0575
H	4.2476	9.2295	-1.2728
H	2.3853	7.6099	0.5248
H	4.523	8.282	1.6374
H	4.1808	9.956	1.2413
H	1.0847	8.3685	-4.0385
H	4.3579	7.6464	-3.2728
H	3.548	9.3824	3.3709
H	-4.5314	7.5924	1.3132
H	-3.8155	8.7798	-0.5658
H	-1.8633	6.6021	-1.3691
H	-1.3299	9.2436	0.0728
H	-1.1666	6.3495	0.9795
H	-0.5243	7.2557	3.2181
H	0.7081	7.3245	1.9958
H	-4.1647	7.2879	-2.4145
H	-1.0347	8.4931	-2.6105
H	0.9111	9.2875	2.5588
H	-7.0836	2.4132	2.8114
H	-7.2987	3.8715	0.9563
H	-4.4135	3.3545	0.1173
H	-5.6339	5.8035	1.4307
H	-3.7198	3.719	2.5874
H	-3.1857	5.9752	3.4671
H	-4.8565	6.219	3.9343
H	-6.8515	2.5076	-0.8826
H	-5.0856	5.422	-1.0735
H	-3.3951	5.4492	5.6289
H	-4.7543	-3.1896	4.0267
H	-6.426	-2.2083	2.5631
H	-4.2043	-0.4872	1.3806
H	-6.6284	0.3514	3.0074
H	-3.73	0.2847	3.9467
H	-5.1436	2.0399	5.0195
H	-6.3121	0.8275	5.4938
H	-5.5362	-2.7749	0.4669
H	-6.2273	0.5228	0.4622
H	-4.7655	1.4157	7.1225
H	1.2084	-4.8443	3.5969
H	-0.9478	-5.5785	2.9076
H	-1.3994	-2.9024	1.5362
H	-2.7558	-3.939	4.08
H	-0.6468	-1.7772	3.789
H	-2.2474	-1.3655	5.6896
H	-1.814	-2.9151	6.3737
H	-0.7232	-5.3329	0.5432
H	-3.6026	-3.8591	1.465
H	6.1698	-1.8952	2.296
H	5.0593	-3.9047	1.7983
H	2.704	-2.449	0.5589
H	2.9401	-3.6825	3.3447
H	2.764	-0.8019	2.4005
H	4.6523	-3.8218	-0.5541
H	1.7679	-4.6219	0.98
H	8.3637	2.7035	-0.8699
H	7.7989	0.5134	-1.5897
H	4.8775	0.9785	-0.8427
H	6.922	-0.7915	0.5464
H	5.7409	1.8701	1.482
H	6.231	0.3485	3.4002
H	7.8813	0.159	2.8324
H	6.3285	0.9404	-3.309
H	5.1755	-1.4846	-1.0541
H	7.9844	2.5368	2.9878

H	1.4	-0.7703	4.2937
H	2.9435	-0.1612	4.8072
O	2.4336	-2.0278	5.5146
H	1.5381	-2.0162	5.9054
C	5.244	-3.6791	-5.778
C	5.9443	-2.5255	-5.0416
C	5.4223	-1.1528	-5.5313
C	5.4835	-1.069	-7.0729
C	4.8228	-2.3228	-7.7077
C	4.9653	-2.3595	-9.2458
O	5.8542	-2.7023	-3.6364
O	6.1561	-0.0886	-4.9283
O	4.8068	0.1287	-7.4842
O	5.3862	-3.5367	-7.1689
O	4.3304	-3.5015	-9.8023
C	0.9722	-6.3193	-3.9156
C	2.0766	-5.8986	-2.9296
C	2.8756	-4.679	-3.4481
C	3.3301	-4.9075	-4.9088
C	2.1233	-5.3456	-5.7747
C	2.5185	-5.6498	-7.2387
O	1.5461	-5.7076	-1.6265
O	3.9642	-4.3961	-2.5681
O	3.8848	-3.6884	-5.4223
O	1.4998	-6.5136	-5.2013
O	1.384	-6.0479	-7.9952
C	-4.257	-5.2757	-3.599
C	-3.4583	-5.6616	-2.3343
C	-1.957	-5.298	-2.4628
C	-1.3915	-5.7843	-3.8167
C	-2.2839	-5.2294	-4.95
C	-1.8178	-5.6115	-6.3704
O	-4.0571	-5.1233	-1.1609
O	-1.2223	-5.8034	-1.349
O	-0.0333	-5.3361	-3.9438
O	-3.6245	-5.727	-4.7669
O	-2.5651	-4.8788	-7.3303
C	-7.2618	-0.9267	-4.271
C	-7.2146	-1.5189	-2.8554
C	-5.9256	-2.3465	-2.65
C	-5.7199	-3.3509	-3.8036
C	-5.8833	-2.6516	-5.1769
C	-5.8277	-3.6238	-6.3751
O	-7.3907	-0.4922	-1.8901
O	-5.9212	-3.008	-1.3866
O	-4.3981	-3.8826	-3.6476
O	-7.1351	-1.9417	-5.23
O	-5.8558	-2.9331	-7.6195
C	-6.097	3.9092	-6.1365
C	-6.724	3.761	-4.7206
C	-6.3867	2.4033	-4.0492
C	-6.56	1.2504	-5.0555
C	-5.6711	1.5572	-6.2795
C	-5.5413	0.4165	-7.317
O	-6.412	4.8659	-3.8756
O	-7.1609	2.2032	-2.8652
O	-6.2249	0.015	-4.4115
O	-6.1862	2.7386	-6.9127
O	-6.7743	-0.1709	-7.7075
C	-2.0661	7.0667	-7.5819
C	-2.7406	7.4562	-6.2488
C	-3.4737	6.2527	-5.606
C	-4.3533	5.508	-6.6291
C	-3.5595	5.2413	-7.9326
C	-4.3487	4.5347	-9.0539
O	-1.8071	8.0527	-5.3584
O	-4.2265	6.6492	-4.4576
O	-4.7547	4.2798	-6.0084
O	-2.9877	6.4585	-8.4488
C	3.0493	5.6514	-8.2183
C	2.7336	6.5395	-7.0037
C	1.2578	6.3835	-6.5771
C	0.3073	6.5445	-7.7796
C	0.7704	5.6285	-8.9392
C	-0.0841	5.7855	-10.2125
O	3.6529	6.3194	-5.9396
O	0.9201	7.2991	-5.5424
O	-1.0032	6.1879	-7.32
O	2.1476	5.9009	-9.2657
C	5.4124	0.9066	-8.4831
C	6.0866	2.1442	-7.8475
C	5.0307	3.1363	-7.3068
C	3.9917	3.4465	-8.402
C	3.4013	2.1227	-8.9507
C	2.3054	2.3107	-10.0281
O	7.0418	1.8036	-6.8518
O	5.6418	4.3158	-6.7808
O	2.9869	4.2991	-7.8386
O	4.4557	1.2754	-9.4493
O	2.6552	3.2132	-11.0696
H	5.7504	-4.6284	-5.5757
H	7.0061	-2.6003	-5.2885
H	4.3802	-1.0336	-5.2264
H	6.5372	-1.0318	-7.3626
H	3.7559	-2.2964	-7.4846
H	4.5082	-1.4734	-9.6886
H	6.0185	-2.3528	-9.5329
H	5.8746	-1.8188	-3.2181
H	6.2837	0.6013	-5.6086
H	4.8208	-4.2837	-9.5107
H	0.5955	-7.312	-3.6485
H	2.759	-6.7492	-2.8572

H	2.2135	-3.812	-3.4143
H	4.0614	-5.7188	-4.9229
H	1.4045	-4.5231	-5.8041
H	2.9515	-4.7616	-7.6997
H	3.2783	-6.4317	-7.2841
H	2.2335	-5.2682	-1.0931
H	4.5961	-3.8126	-3.0398
H	1.4653	-5.7414	-8.9188
H	-5.2136	-5.8072	-3.6214
H	-3.5383	-6.748	-2.2471
H	-1.876	-4.209	-2.4157
H	-1.454	-6.8741	-3.862
H	-2.2681	-4.1377	-4.8981
H	-0.7639	-5.3729	-6.5088
H	-1.926	-6.6839	-6.5406
H	-3.3878	-5.155	-0.4532
H	-0.2674	-5.7711	-1.5722
H	-2.5653	-5.3588	-8.1807
H	-8.2553	-0.5119	-4.4698
H	-8.0766	-2.184	-2.7639
H	-5.0921	-1.6431	-2.6374
H	-6.4867	-4.1244	-3.7168
H	-5.0539	-1.9543	-5.2941
H	-4.9114	-4.2103	-6.3398
H	-6.6527	-4.3368	-6.3336
H	-6.9443	-0.7794	-1.0692
H	-5.2702	-3.7392	-1.4341
H	-5.6678	-3.5929	-8.3186
H	-6.6767	4.6173	-6.7382
H	-7.8054	3.7938	-4.874
H	-5.3416	2.4333	-3.7283
H	-7.5947	1.2385	-5.4064
H	-4.6518	1.7556	-5.9386
H	-5.0119	0.772	-8.2029
H	-4.9129	-0.37	-6.9008
H	-6.4733	4.5488	-2.9567
H	-7.1764	1.2435	-2.6723
H	-6.6386	-1.1386	-7.7094
H	-1.7572	7.9554	-8.141
H	-3.4792	8.2239	-6.4912
H	-2.714	5.5594	-5.2487
H	-5.2093	6.1485	-6.8548
H	-2.7501	4.5548	-7.695
H	-3.7167	4.4594	-9.9402
H	-2.2231	8.0788	-4.4772
H	-4.919	5.9733	-4.3115
H	4.0219	5.9313	-8.6378
H	2.8908	7.5703	-7.3308
H	1.1408	5.3786	-6.167
H	0.3472	7.5858	-8.1085
H	0.6618	4.5936	-8.613
H	3.3386	6.8518	-5.1855
H	-0.0486	7.4424	-5.5713
H	6.1237	0.3267	-9.0801
H	6.6439	2.6337	-8.6503
H	4.5357	2.6496	-6.4631
H	4.4903	3.9368	-9.2423
H	2.91	1.5906	-8.1374
H	2.0277	1.3465	-10.4574
H	1.4018	2.6877	-9.5508
H	7.2803	2.6384	-6.4166
H	4.9345	4.9542	-6.5646
H	1.8976	3.8179	-11.2007
H	-4.5821	3.5086	-8.7667
H	-0.0033	6.7993	-10.6082
H	-1.137	5.6244	-9.9808
O	0.2753	4.8489	-11.2202
H	-0.3978	4.9309	-11.9254
O	-0.3058	-1.4967	6.3646
H	-0.5702	-1.0567	7.1967
O	-5.5519	5.2014	-9.3988
H	-5.7849	4.9173	-10.2964
C	-2.4207	1.1271	-12.039
C	-3.1627	2.3804	-12.5427
H	-3.5594	2.9469	-11.7005
H	-2.437	3.0281	-13.0356
N	-4.9742	3.2384	-14.1499
H	-5.4642	3.7865	-13.4402
C	-4.0451	4.1685	-14.9456
H	-3.4717	4.6648	-14.1736
H	-3.3827	3.4989	-15.485
C	-4.759	5.1678	-15.8483
C	-4.2169	6.3034	-16.4193
H	-3.236	6.7295	-16.3531
N	-6.0202	5.0825	-16.2412
N	-6.29	6.1106	-17.1329
N	-5.1841	6.8491	-17.2198
C	-5.0314	8.0019	-18.1187
H	-4.7452	8.8606	-17.5206
H	-4.1735	7.7858	-18.7428
C	-6.2013	8.3685	-19.0686
H	-5.8497	9.0213	-19.8716
H	-6.7031	7.5137	-19.5228
N	-7.2773	9.1712	-18.3229
H	-6.8539	9.7694	-17.621
C	-8.2568	9.9791	-19.1856
H	-7.6508	10.6678	-19.7787
H	-8.7244	9.2543	-19.8572
C	-11.1117	12.0064	-16.6812
C	-11.4067	10.7295	-17.1518
C	-10.4754	10.081	-17.9761
C	-9.2688	10.6999	-18.312

C	-8.9991	11.9846	-17.8336
C	-9.92	12.6481	-17.009
H	-11.8235	12.5107	-16.046
H	-10.6687	9.086	-18.3496
H	-8.0651	12.4555	-18.1017
O	-12.6144	10.1917	-16.7563
O	-9.7347	13.9099	-16.4815
C	-12.9703	8.8828	-17.1983
H	-13.9554	8.6227	-16.8108
H	-13.0228	8.8205	-18.2866
H	-12.2695	8.1319	-16.8321
C	-8.5254	14.6119	-16.7686
H	-8.5415	15.5828	-16.2733
H	-7.6487	14.0753	-16.402
H	-8.4078	14.7954	-17.8379
H	-5.7101	2.8883	-14.7691
H	-7.8366	8.5226	-17.7551
C	-0.8173	-2.1407	-8.8799
C	-1.3346	-3.0777	-9.9814
H	-0.673	-3.0153	-10.8461
H	-1.2848	-4.1083	-9.6287
N	-3.4714	-3.8834	-11.108
H	-4.4292	-3.5739	-11.314
C	-2.8576	-4.3824	-12.4224
H	-2.6929	-3.4807	-12.9967
H	-1.9024	-4.8119	-12.1402
C	-3.7815	-5.3641	-13.1158
C	-4.3487	-5.3158	-14.3815
H	-4.2887	-4.5963	-15.1747
N	-4.9505	-7.2004	-13.4129
N	-4.1803	-6.4751	-12.5298
N	-5.023	-6.5008	-14.5379
C	-5.8374	-6.9724	-15.6626
H	-6.871	-6.7888	-15.4011
H	-5.62	-6.3587	-16.5307
C	-5.6989	-8.4705	-16.0339
H	-5.8233	-9.1506	-15.187
H	-6.4048	-8.7548	-16.8181
N	-4.2995	-8.7377	-16.6046
H	-3.6251	-8.6372	-15.836
C	-4.0574	-10.1031	-17.2612
H	-4.4551	-10.8434	-16.5621
H	-4.6574	-10.1197	-18.1738
C	0.175	-10.5661	-17.9009
C	-0.5844	-9.8872	-18.8524
C	-1.9675	-9.7579	-18.653
C	-2.5739	-10.2968	-17.516
C	-1.7954	-10.9913	-16.5899
C	-0.4194	-11.1222	-16.7597
H	1.2364	-10.6463	-18.0707
H	-2.5783	-9.2236	-19.3653

H	-2.2571	-11.4157	-15.7098
O	0.103	-9.3706	-19.9317
O	0.2629	-11.7876	-15.7617
C	-0.6136	-8.6387	-20.9259
H	0.0776	-8.3004	-21.6979
H	-1.3701	-9.2544	-21.4153
H	-1.0933	-7.7518	-20.5084
C	1.6789	-11.9307	-15.8645
H	2.0536	-12.4724	-14.9959
H	1.9653	-12.5004	-16.7501
H	2.1834	-10.9633	-15.8858
H	-3.5775	-4.6631	-10.4577
H	-4.0161	-7.9895	-17.2303
C	3.4253	5.6674	5.2086
C	4.6112	6.6306	5.0233
H	4.7949	6.8039	3.9626
H	4.336	7.6015	5.436
N	6.9925	7.1522	5.746
H	7.1322	7.6068	4.8388
C	6.8481	8.1884	6.8718
H	5.9739	8.7696	6.6016
H	6.614	7.5997	7.7596
C	8.1237	8.9972	7.0544
C	8.8781	9.1802	8.2022
H	8.7646	8.8084	9.2024
N	9.9057	10.1865	6.5255
N	8.7334	9.6197	6.0612
N	9.9578	9.9403	7.8354
C	11.1333	10.2804	8.6413
H	10.9718	9.8813	9.6326
H	11.9795	9.7232	8.2592
C	11.497	11.7771	8.7878
H	10.6715	12.3966	9.135
H	12.3324	11.8996	9.4816
N	11.9626	12.3925	7.4531
H	11.1244	12.5774	6.9063
C	12.7304	13.723	7.5642
H	13.5332	13.5692	8.2886
H	13.1841	13.8565	6.5791
C	10.1441	17.02	8.5656
C	10.4282	16.7602	7.2276
C	11.2767	15.6888	6.9116
C	11.8251	14.8923	7.9208
C	11.5402	15.1862	9.2567
C	10.6866	16.2484	9.5899
H	9.4897	17.8406	8.8155
H	11.5103	15.458	5.883
H	11.9736	14.5705	10.0298
O	9.839	17.5966	6.3013
O	10.3363	16.6007	10.8776
C	10.1257	17.4128	4.9157

H	9.6123	18.1783	4.3338
H	11.1923	17.5066	4.7048
H	9.776	16.4448	4.556
C	10.8143	15.8104	11.9656
H	10.4232	16.2064	12.9027
H	10.4846	14.773	11.8893
H	11.9031	15.8322	12.0331
H	7.8665	6.6545	5.9333
H	12.505	11.7118	6.9177
C	-1.6195	2.3354	4.9357
C	-1.2879	1.9985	6.3962
H	-1.683	1.0103	6.6407
H	-0.2037	1.9294	6.5032
N	-1.3442	2.6208	8.793
H	-0.3429	2.4091	8.7073
C	-1.5489	3.6619	9.9136
H	-1.1659	4.5949	9.5127
H	-2.6233	3.7331	10.0401
C	-0.8565	3.292	11.2134
C	-0.6562	4.0862	12.3346
H	-0.9279	5.0959	12.5848
N	0.1972	2.0985	12.7278
N	-0.3335	2.1025	11.4549
N	-0.0428	3.2924	13.2617
C	0.443	3.6012	14.6102
H	0.5352	2.6681	15.171
H	1.4543	3.9843	14.5066
C	-0.3857	4.6408	15.4063
H	0.0371	4.787	16.403
H	-0.4654	5.6172	14.9229
N	-1.8293	4.152	15.6143
H	-2.3653	4.3105	14.7538
C	-2.6016	4.7953	16.7764
H	-2.0189	4.5984	17.6792
H	-2.5862	5.8703	16.5795
C	-6.5909	3.1993	17.0624
C	-6.4002	4.4436	16.4669
C	-5.0983	4.9596	16.3776
C	-4.0114	4.2379	16.8788
C	-4.2309	2.9967	17.4825
C	-5.5248	2.463	17.5722
H	-7.5891	2.7952	17.1296
H	-4.9173	5.9176	15.9132
H	-3.3845	2.449	17.8692
O	-7.532	5.0769	15.9962
O	-5.8319	1.2383	18.1291
C	-7.4107	6.366	15.3978
H	-6.8092	6.331	14.4894
H	-6.9779	7.0953	16.0846
H	-8.3987	6.7314	15.1175
C	-4.7764	0.4228	18.6374
H	-5.1902	-0.5075	19.0267
H	-4.0585	0.1582	17.8592
H	-4.2473	0.9106	19.4577
H	-1.7875	1.7316	9.0413
H	-1.8571	3.1394	15.6947
C	5.0739	9.6653	9.2478
O	4.4755	8.6716	8.8283
C	6.1626	11.1491	10.7091
H	5.7954	11.5791	11.6471
C	5.6276	11.8851	9.5285
H	4.7009	12.4149	9.7695
C	7.7951	12.5927	10.0876
N	5.3701	10.8102	8.5671
N	5.5662	9.8487	10.4992
N	6.7057	12.8324	9.3132
N	7.609	11.336	10.5892
O	8.7326	13.3649	10.303
C	8.573	10.8354	11.6112
H	8.1609	11.1023	12.5873
H	9.4897	11.4311	11.5549
C	5.6676	8.763	11.4847
H	5.5543	9.196	12.4806
H	4.7942	8.1128	11.4003
C	8.0871	8.3298	12.1965
H	7.8554	8.4858	13.2552
C	8.935	7.119	11.9898
H	8.8573	6.4173	12.8258
N	6.8871	7.9425	11.4758
N	8.9507	9.3978	11.695
C	10.2315	9.0643	12.0306
O	11.1745	9.816	12.293
C	7.0374	6.8432	10.6903
O	6.1671	6.252	10.0475
C	6.6975	13.8646	8.2611
H	5.7283	14.366	8.2817
H	7.4084	14.6611	8.5031
C	4.7122	11.064	7.2539
H	3.9485	11.8261	7.4249
H	4.1128	10.2017	6.9455
N	6.9643	13.4115	6.8934
C	5.942	12.9201	5.9929
H	5.073	13.5846	5.9905
C	6.6608	12.9661	4.6828
H	6.3442	13.8214	4.0802
N	6.2407	11.7147	4.0767
N	5.5333	11.5285	6.1083
N	8.0457	13.1274	5.102
C	9.1654	13.3778	4.1868
C	6.725	11.2602	2.7556
C	10.3791	6.4077	4.3325
O	9.2139	6.004	4.3577

C	12.3195	7.599	3.7298
H	12.9232	7.2535	2.8864
C	12.6145	6.8126	4.9646
H	13.516	6.209	4.8301
C	12.8257	9.1028	5.4637
N	11.4395	5.9585	5.0506
N	10.9137	7.3201	3.4883
N	12.8435	7.8433	5.9749
N	12.7108	8.9397	4.1249
O	12.9613	10.166	6.0727
C	12.6806	10.0854	3.1966
H	12.9617	9.7315	2.2029
H	13.4963	10.7649	3.4624
C	10.1867	7.8652	2.3285
H	10.8664	7.9003	1.4749
H	9.4081	7.1713	2.0025
C	10.3129	10.4278	2.2173
H	10.6175	10.4362	1.1676
C	9.2832	11.4731	2.495
H	9.1714	12.148	1.6419
N	9.5947	9.1971	2.5172
N	11.4164	10.8447	3.0731
C	11.169	11.9784	3.7785
O	11.9736	12.6645	4.4141
C	8.327	9.3812	2.9567
O	7.5546	8.5467	3.4316
C	13.4815	7.5537	7.2814
H	14.2804	6.832	7.098
H	14.0278	8.4369	7.6281
C	11.3247	4.8632	6.0283
H	12.2318	4.2582	5.9778
H	10.5299	4.1729	5.7285
N	12.6389	7.006	8.3738
C	12.1613	5.6275	8.3502
H	12.9973	4.9252	8.2844
C	11.4434	5.4947	9.654
H	11.683	4.5519	10.1527
N	10.0478	5.514	9.2462
N	11.1019	5.2634	7.4257
N	11.9663	6.6276	10.392
C	11.5141	6.9467	11.7611
C	8.916	5.3213	10.1634
H	12.3257	7.4552	12.2902
H	11.4138	6.008	12.3094
H	9.2351	4.6478	10.9616
H	8.1397	4.7474	9.6464
H	9.8838	14.0386	4.6806
H	8.7978	13.982	3.3549
H	6.6686	12.1031	2.0644
H	6.0243	10.5365	2.3368
N	9.8357	12.1846	3.6361

N	8.0763	10.6804	2.6751
N	8.3596	6.5433	10.7805
N	10.2621	7.7118	11.9429
C	12.7833	7.4312	9.6614
O	13.4728	8.3666	10.0753
C	9.8876	5.6081	7.9095
O	8.8597	5.8563	7.2902
C	8.2024	13.1226	6.4418
O	9.2403	12.9837	7.0791
C	5.4235	10.9715	4.8688
O	4.7388	10.0016	4.5302
C	-7.8006	-8.1182	-12.4835
O	-7.8317	-8.521	-13.6482
C	-7.2857	-7.9217	-10.1965
H	-7.7329	-8.6773	-9.545
C	-8.2832	-6.8674	-10.5458
H	-9.1601	-6.9181	-9.895
C	-6.3358	-5.8206	-9.734
N	-8.6366	-7.2175	-11.9116
N	-6.9518	-8.5058	-11.49
N	-7.5509	-5.6368	-10.3036
N	-6.2856	-7.1504	-9.4708
O	-5.513	-4.9566	-9.427
C	-5.1742	-7.7667	-8.72
H	-5.5696	-8.6071	-8.1468
H	-4.8223	-7.0701	-7.9519
C	-6.1456	-9.7442	-11.6049
H	-6.4765	-10.421	-10.8144
H	-6.426	-10.2855	-12.514
C	-3.9971	-9.5127	-10.2116
H	-4.3094	-10.3197	-9.5432
C	-2.5457	-9.6418	-10.5541
H	-2.0552	-10.4006	-9.9392
N	-4.6682	-9.637	-11.5027
N	-4.0214	-8.2388	-9.5083
C	-2.9667	-7.4482	-9.8217
O	-2.8467	-6.2322	-9.6702
C	-3.8568	-10.2035	-12.4389
O	-4.1833	-10.7304	-13.5077
C	-8.1533	-4.3069	-10.5058
H	-9.204	-4.3475	-10.2122
H	-7.7184	-3.5829	-9.8094
C	-9.6601	-6.4853	-12.6881
H	-10.4523	-6.1786	-12.0026
H	-10.1633	-7.198	-13.3486
N	-8.0635	-3.7731	-11.8738
C	-9.0784	-3.9699	-12.8952
H	-10.0352	-3.5604	-12.561
C	-8.5177	-3.2061	-14.0612
H	-9.2685	-2.5383	-14.4941
N	-8.1568	-4.2602	-15.0064

N	-9.2476	-5.2936	-13.4658
N	-7.4728	-2.4379	-13.4111
C	-6.8177	-1.2697	-14.0159
C	-7.6105	-4.0065	-16.3689
C	-2.0562	-5.4659	-16.8274
O	-2.9416	-6.3009	-17.0127
C	-0.9915	-3.3776	-16.6256
H	-0.4147	-2.8933	-17.418
C	-0.1765	-4.4019	-15.8947
H	0.861	-4.3812	-16.2404
C	-0.6213	-2.662	-14.4272
N	-0.8109	-5.6407	-16.3212
N	-2.0691	-4.1685	-17.2026
N	-0.2487	-3.9742	-14.4948
N	-1.3081	-2.4325	-15.5738
O	-0.4311	-1.866	-13.5048
C	-2.2693	-1.3219	-15.745
H	-2.0702	-0.8493	-16.7089
H	-2.0521	-0.5233	-15.0298
C	-3.0717	-3.6207	-18.1359
H	-2.6236	-2.784	-18.6753
H	-3.258	-4.3598	-18.9214
C	-4.5173	-1.9031	-16.8559
H	-4.4345	-1.0694	-17.5552
C	-5.903	-2.0346	-16.2977
H	-6.6013	-1.3701	-16.8123
N	-4.3534	-3.1573	-17.5603
N	-3.705	-1.6541	-15.6756
C	-4.4141	-1.5907	-14.5269
O	-3.9727	-1.4729	-13.3861
C	-5.5141	-3.8487	-17.6538
O	-5.8047	-4.7023	-18.4963
C	0.643	-4.5402	-13.4398
H	1.6529	-4.5404	-13.8555
H	0.7247	-3.8396	-12.6032
C	-0.2152	-6.9658	-16.0995
H	0.8344	-6.9333	-16.3967
H	-0.662	-7.6946	-16.7828
N	0.42	-5.9077	-12.8999
C	0.6191	-7.1428	-13.6541
H	1.6531	-7.226	-14
C	0.2938	-8.2085	-12.6389
H	1.1345	-8.8881	-12.476
N	-0.8117	-8.9059	-13.2687
N	-0.3025	-7.479	-14.7261
N	0.0118	-7.4255	-11.4505
C	-0.5827	-7.9826	-10.2153
C	-1.3888	-10.1669	-12.7806
H	-0.3982	-7.31	-9.3743
H	-0.0228	-8.8817	-9.9516
H	-0.6268	-10.7037	-12.2126
H	-1.5761	-10.8172	-13.6374
H	-6.477	-0.6034	-13.2174
H	-7.5642	-0.6782	-14.5494
H	-8.3104	-3.3223	-16.8544
H	-7.7074	-4.924	-16.9579
N	-5.7073	-1.5657	-14.9342
N	-6.2501	-3.4304	-16.5806
N	-2.5995	-10.0637	-11.9426
N	-2.0204	-8.3189	-10.2337
C	0.5023	-6.1679	-11.5642
O	0.8856	-5.4247	-10.657
C	-1.3527	-8.1886	-14.2684
O	-2.4906	-8.2467	-14.7078
C	-7.0009	-3.0667	-12.3105
O	-5.9055	-2.9554	-11.7745
C	-8.9994	-5.3143	-14.7985
O	-9.4104	-6.1367	-15.6223
C	-3.0488	9.9753	-15.6149
O	-3.5161	10.7674	-16.4388
C	-2.4084	9.0963	-13.5297
H	-1.9667	9.6994	-12.7313
C	-1.4013	8.7632	-14.5785
H	-0.5336	9.4274	-14.5171
C	-1.8698	6.8293	-13.3137
N	-2.1263	8.9924	-15.8255
N	-3.3594	9.8894	-14.296
N	-1.0206	7.418	-14.1928
N	-2.7905	7.7819	-13.0162
O	-1.7865	5.6913	-12.8461
C	-3.6582	7.6387	-11.8243
H	-3.427	8.4473	-11.1279
H	-3.4099	6.7401	-11.2498
C	-4.585	10.4969	-13.7334
H	-4.3142	10.9954	-12.8009
H	-4.9324	11.3169	-14.3694
C	-5.8738	8.8687	-12.2313
H	-5.7546	9.5229	-11.3638
C	-7.2798	8.3643	-12.3385
H	-7.9214	8.834	-11.5886
N	-5.7248	9.6062	-13.4711
N	-5.1066	7.6477	-12.0673
C	-5.8363	6.5231	-12.2446
O	-5.4343	5.3689	-12.3825
C	-6.6134	9.2241	-14.4157
O	-6.5178	9.3412	-15.6348
C	0.0517	6.6719	-14.8697
H	0.8616	7.3664	-15.101
H	0.5112	5.9795	-14.1572
C	-1.4687	8.8639	-17.156
H	-0.4654	9.2838	-17.054
H	-1.9422	9.5472	-17.8679

N	-0.2959	5.9378	-16.0983
C	-0.2839	6.5508	-17.4131
H	0.7175	6.9308	-17.6396
C	-0.6426	5.3808	-18.2692
H	0.2465	4.8939	-18.682
N	-1.3966	6.0313	-19.3261
N	-1.3027	7.528	-17.7901
N	-1.3234	4.4944	-17.3296
C	-1.8244	3.1567	-17.736
C	-1.9862	5.3203	-20.4764
C	-8.9152	5.1902	-19.0896
O	-9.1009	6.2583	-19.6816
C	-8.3029	2.9845	-18.8544
H	-8.6693	2.4453	-19.7332
C	-9.4077	3.2296	-17.8801
H	-10.276	2.5984	-18.0866
C	-7.5634	2.3163	-16.7379
N	-9.7117	4.6264	-18.1428
N	-7.8686	4.3273	-19.2238
N	-8.8016	2.8492	-16.6083
N	-7.4234	2.1369	-18.0729
O	-6.7818	1.9916	-15.8441
C	-6.3138	1.3785	-18.6642
H	-6.6372	0.9828	-19.6288
H	-6.1449	0.4837	-18.0635
C	-6.8343	4.5605	-20.2643
H	-7.0706	3.889	-21.093
H	-6.9795	5.5449	-20.7178
C	-4.8108	3.0125	-19.9559
H	-4.9995	2.5207	-20.9152
C	-3.3649	3.286	-19.7666
H	-2.7478	2.6018	-20.3578
N	-5.3983	4.3518	-19.9385
N	-5.0385	2.0898	-18.8594
C	-4.054	2.1054	-17.9214
O	-3.9635	1.3843	-16.9254
C	-4.4742	5.2575	-20.3791
O	-4.6753	6.395	-20.8091
C	-9.5407	2.8386	-15.33
H	-10.5876	2.6054	-15.5336
H	-9.2065	1.9965	-14.7153
C	-10.679	5.4058	-17.341
H	-11.564	4.7885	-17.1764
H	-11.0545	6.2459	-17.9325
N	-9.4859	4.0772	-14.5307
C	-10.3615	5.2104	-14.7833
H	-11.4053	4.9367	-14.6109
C	-9.8912	6.235	-13.7893
H	-10.7124	6.5643	-13.148
N	-9.4506	7.3282	-14.6494
N	-10.2146	5.9215	-16.0419
N	-8.9083	5.4827	-13.0206
C	-8.239	6.0301	-11.8247
C	-9.043	8.666	-14.1793
H	-7.9074	5.2098	-11.181
H	-8.9841	6.5484	-11.2183
H	-9.7291	8.9782	-13.3898
H	-9.2032	9.4038	-14.9726
H	-1.7283	2.4436	-16.9109
H	-1.1139	2.7579	-18.4638
H	-1.2552	4.5898	-20.8282
H	-2.0804	6.0058	-21.3241
N	-3.1742	3.0575	-18.3418
N	-3.274	4.6297	-20.2897
N	-7.6627	8.7838	-13.6787
N	-7.1093	6.9461	-12.0642
C	-8.5465	4.302	-13.5815
O	-7.6272	3.558	-13.2439
C	-9.3804	6.9752	-15.9476
O	-8.7881	7.542	-16.8546
C	-0.7994	4.6765	-16.0853
O	-0.7944	3.8807	-15.1442
C	-1.548	7.364	-19.1208
O	-1.8774	8.2185	-19.9483
C	-3.6075	0.0692	10.383
O	-3.1563	0.6473	9.3999
C	-3.5377	-1.0011	12.4492
H	-3.8466	-2.0041	12.7539
C	-4.7071	-0.0632	12.4276
H	-5.6229	-0.5644	12.7508
C	-3.033	0.8544	13.8137
N	-4.7751	0.2908	11.0188
N	-3.1116	-0.9898	11.0607
N	-4.3148	0.9643	13.379
N	-2.6678	-0.3975	13.4453
O	-2.3865	1.6511	14.4935
C	-1.4768	-1.0717	13.991
H	-1.7303	-2.1234	14.1382
H	-1.3169	-0.7075	15.011
C	-2.1129	-1.9233	10.5226
H	-2.2983	-2.915	10.9391
H	-2.2852	-2.0521	9.4492
C	0.003	-1.8403	12.0108
H	-0.0965	-2.8993	12.265
C	1.4149	-1.5122	11.6373
H	2.0211	-2.416	11.5334
N	-0.7047	-1.5663	10.765
N	-0.2213	-1.0189	13.2008
C	0.9636	-0.716	13.798
O	1.1747	-0.4471	14.9848
C	-0.0094	-0.7965	9.895
O	-0.4255	-0.2544	8.8686

C	-5.1827	2.1189	13.6734
H	-6.2035	1.7605	13.818
H	-4.9097	2.5269	14.6495
C	-5.9148	0.9818	10.3988
H	-6.8273	0.6829	10.9179
H	-6.05	0.5836	9.3884
N	-5.2013	3.2071	12.6755
C	-6.1124	3.2853	11.536
H	-7.1486	3.1964	11.8723
C	-5.8226	4.6523	10.9884
H	-6.7446	5.21	10.8004
N	-5.1117	4.3816	9.7449
N	-5.8685	2.4573	10.3659
N	-5.0783	5.2342	12.0851
C	-4.8066	6.6698	12.2223
C	-4.6622	5.4599	8.8257
C	1.2704	5.5467	8.4056
O	0.7848	4.9026	7.4793
C	1.5986	7.2164	10.026
H	2.0521	8.1682	9.7392
C	2.6266	6.1537	10.2284
H	3.6272	6.5853	10.2198
C	1.5746	6.5064	12.2648
N	2.4522	5.3394	9.0397
N	0.7916	6.6975	8.935
N	2.3206	5.6148	11.5534
N	1.0015	7.315	11.3402
O	1.4298	6.5665	13.4877
C	-0.1698	8.1639	11.6277
H	-0.0232	9.1117	11.1057
H	-0.1439	8.4664	12.6797
C	-0.2623	7.5033	8.2819
H	0.0154	8.5567	8.3531
H	-0.246	7.3161	7.2032
C	-2.1331	7.9906	10.0068
H	-2.1661	9.0782	9.8938
C	-3.5061	7.4105	10.1247
H	-4.2679	8.1392	9.831
N	-1.6453	7.3692	8.7892
N	-1.505	7.6341	11.2708
C	-2.4099	7.2963	12.2239
O	-2.2015	7.149	13.4317
C	-2.5998	6.6348	8.1677
O	-2.6319	6.3065	6.978
C	3.2872	4.7387	12.2688
H	4.2832	5.1405	12.0679
H	3.2007	4.8705	13.3507
C	3.3811	4.2566	8.669
H	4.4018	4.6374	8.7338
H	3.2601	3.9961	7.6142
N	3.3474	3.2935	11.9436
C	3.9623	2.7994	10.7178
H	5.0088	3.1121	10.6533
C	3.8443	1.3121	10.8539
H	4.7902	0.8095	10.6363
N	2.8548	0.9839	9.8413
N	3.2847	3.0191	9.4546
N	3.4854	1.1695	12.2538
C	3.2138	-0.1365	12.8912
C	2.4104	-0.3862	9.5455
H	3.5068	-0.0849	13.9442
H	3.9222	-0.8575	12.479
H	3.2505	-1.0688	9.6865
H	2.1841	-0.4686	8.4772
H	-4.7841	6.917	13.2878
H	-5.6642	7.2283	11.8426
H	-5.5072	6.1429	8.7133
H	-4.5537	5.0443	7.8192
N	-3.591	7.1759	11.5603
N	-3.4773	6.3044	9.1601
N	1.2672	-0.8666	10.3406
N	1.862	-0.7289	12.7804
C	3.4585	2.3506	12.9215
O	3.4994	2.5188	14.1431
C	2.3478	2.0735	9.2264
O	1.3257	2.1617	8.5571
C	-4.4011	4.288	12.7693
O	-3.3569	4.4081	13.3954
C	-5.4575	3.1507	9.2732
O	-5.3845	2.7328	8.1151
H	-0.4961	0.5545	-11.2449
H	-0.595	2.2554	-11.6148
H	-2.1009	0.5498	-12.9081
H	-3.105	0.4703	-11.4979
H	-1.4941	-2.2134	-8.0255
H	-0.8626	-1.1064	-9.2216
H	0.6791	-3.5208	-8.144
H	1.3322	-2.3047	-9.2218
H	3.0507	5.8234	6.2173
H	3.7505	4.6244	5.1764
H	1.3368	5.4921	4.6777
H	2.0824	6.9436	4.0518
H	0.0795	1.3253	4.0394
H	-1.3151	0.3	4.2889
H	-1.25	3.3325	4.6855
H	-2.7025	2.3721	4.8098
C	-4.3006	2.0039	-13.513
H	-3.9503	1.4035	-14.3543
H	-5.108	1.4621	-13.0158
C	-2.7667	-2.7102	-10.4029
H	-3.4129	-2.5127	-9.5438
H	-2.8135	-1.855	-11.0799

C	5.8833	6.0847	5.6947	C	-1.8435	3.0186	7.3931
H	5.7292	5.7542	6.725	H	-1.4711	4.0189	7.1819
H	6.3168	5.2511	5.1368	H	-2.933	3.0307	7.4242

(Py')$_2$ ⊂ (γ-CD)$_2$ (−11465.356930 Hartree)

C	-2.17211902	0.46632105	1.83958498	C	2.59517258	-2.05870288	0.09746726
C	-2.07723195	0.13491754	3.1957318	C	2.47467226	-1.61739863	1.37993704
C	-1.68241946	-1.15048985	3.60546631	C	1.82278788	-1.35990217	-4.72584166
C	-1.37749187	-2.11950215	2.63254039	C	1.64200435	-1.84585241	-5.82337612
C	-1.87386468	-0.52245133	0.85471283	C	1.38702836	-2.42910581	-7.13814213
C	-1.47224451	-1.82997433	1.26522391	C	1.26145052	1.9201655	4.60309473
C	-1.1482332	-2.79828076	0.24993453	C	1.06698041	2.25132081	5.75449635
C	-1.20196911	-2.47846098	-1.07248771	C	0.80345871	2.65643655	7.1320512
C	-2.30116739	1.12798393	-0.93633592	H	2.09912918	-0.48578773	3.81696918
C	-2.27452485	1.45419629	-2.29752924	H	0.83498758	3.36517454	2.39120335
C	-1.88840863	0.51124838	-3.26551289	H	0.77040847	3.83786321	-0.05897642
C	-1.57831659	-0.80016219	-2.86581602	H	0.93180343	3.0233533	-2.37460908
C	-1.58916697	-1.16489247	-1.51380224	H	2.58329703	-2.68715481	-2.54201085
C	-1.93645596	-0.19119758	-0.52874295	H	1.33502326	1.16682973	-3.97630745
C	-2.62323041	2.09728846	0.07729493	H	2.89952746	-3.08329133	-0.10342816
C	-2.54822961	1.78346203	1.40066348	H	2.66993996	-2.28921867	2.21097497
C	-1.70155512	0.91394613	-4.62655497	H	1.21770329	-1.64832839	-7.88963971
C	-1.43242921	1.26871421	-5.75618315	H	2.23556795	-3.03547229	-7.47926852
C	-1.05063615	1.70388491	-7.0971564	H	0.98026038	1.82871336	7.82898224
C	-1.59357149	-1.45604045	5.00029062	H	1.44227297	3.49993039	7.42143448
C	-1.54088397	-1.67688608	6.19282101	C	-6.03850371	-3.8300885	3.64180621
C	-1.49470521	-1.95255808	7.62609505	C	-6.67532133	-3.26479525	2.35491198
H	-2.29096875	0.88649894	3.94990052	C	-6.31055808	-1.79421863	2.14815677
H	-1.05144391	-3.10605604	2.94873962	C	-6.57019503	-1.01510799	3.44218054
H	-0.84412507	-3.79428473	0.56324566	C	-5.81159346	-1.6538601	4.61087569
H	-0.91595198	-3.20762252	-1.8252558	C	-5.9889977	-0.93566939	5.95606245
H	-2.52777578	2.4609217	-2.61396498	O	-6.28586307	-4.10004463	1.27380398
H	-1.26548731	-1.52147341	-3.61464686	O	-7.11474943	-1.29061256	1.08395893
H	-2.91736293	3.09781357	-0.23266211	O	-6.16696811	0.34238334	3.2096554
H	-2.77350667	2.53304929	2.15358926	O	-6.31261615	-3.00661469	4.74738505
H	-1.17054072	0.89530835	-7.82869098	O	-7.26080886	-0.32749968	6.12771257
H	-1.64616209	2.56488655	-7.42111295	C	-1.4556302	-6.61327874	3.82951837
H	-1.88564021	-2.95555858	7.8355933	C	-2.32191415	-6.75617554	2.5667373
H	-0.467694	-1.89599454	8.00943761	C	-3.25240125	-5.56297186	2.37873997
C	2.07328777	-0.26731697	1.67389509	C	-4.01780201	-5.23324689	3.66728748
C	1.91204622	0.18881177	2.98675358	C	-3.07044205	-5.15576464	4.87221745
C	1.47075092	1.49625498	3.25341994	C	-3.76701499	-5.09396376	6.22691854
C	1.20575342	2.36622739	2.18105774	O	-1.44315721	-6.938166	1.47080573
C	1.80622234	0.61929419	0.5873744	O	-4.16061959	-5.87800034	1.32917702
C	1.37610901	1.95494418	0.85305754	O	-4.64568072	-3.97166044	3.4431785
C	1.09048634	2.81842925	-0.26271629	O	-2.26809939	-6.35511268	4.94348529
C	1.19328175	2.37186201	-1.54533215	O	-2.81862233	-5.05477796	7.28439863
C	2.29801386	-1.19608561	-1.01600343	C	3.70826539	-5.31418902	4.14809265
C	2.3088095	-1.65793801	-2.33706281	C	3.17408962	-6.12900795	2.95112317
C	1.94790232	-0.81878728	-3.40617896	C	1.70797373	-5.82374163	2.64354195
C	1.62717468	0.52532521	-3.15064042	C	0.88384935	-5.92060636	3.93146808
C	1.59827467	1.02323177	-1.84161483	C	1.4642586	-4.97720578	4.99303884
C	1.91771782	0.15513023	-0.75397959	C	0.74527275	-5.04235264	6.33449636

O	4.03654631	-5.89925538	1.85176211	O	-6.06163659	4.07502308	1.06878385
O	1.26880274	-6.78228895	1.68873052	O	-4.16458419	4.33329377	3.22489483
O	-0.47790514	-5.60331734	3.62879656	O	-6.76568358	2.11332148	4.60824906
O	2.82718941	-5.3904297	5.24804079	O	-5.13990316	2.27526844	6.85472686
O	0.66493761	-6.36512101	6.84707438	H	-6.4855735	-4.8025453	3.87667435
C	6.91764789	-1.0508161	3.92198006	H	-7.7664128	-3.31407766	2.48583416
C	6.99601338	-2.04158768	2.74592546	H	-5.24278939	-1.71515564	1.89555098
C	5.68922789	-2.82041606	2.60033372	H	-7.63913022	-1.0448198	3.67919923
C	5.25740017	-3.42579249	3.94315127	H	-4.73931744	-1.70261953	4.38496727
C	5.2639998	-2.37199217	5.05669785	H	-5.8555166	-1.69007147	6.74142578
C	5.05990802	-2.93054096	6.46195828	H	-5.19121918	-0.19443675	6.06792385
O	7.32851354	-1.30254505	1.58437763	H	-6.42421424	-3.65391672	0.40970819
O	5.89126131	-3.84958892	1.63717129	H	-7.09518352	-0.30433527	1.11955798
O	3.94099305	-3.97531006	3.76304913	H	-7.1223763	0.62761288	5.99869196
O	6.55792108	-1.74086873	5.09929609	H	-0.95926633	-7.56406986	4.04539184
O	5.14284577	-1.90325372	7.43497337	H	-2.94465421	-7.65228874	2.70687835
C	5.81868026	4.16259706	3.6721977	H	-2.65367765	-4.68128266	2.11596318
C	6.52997274	3.49671702	2.4790526	H	-4.77446725	-6.01282221	3.83803674
C	6.18919471	2.01008414	2.38235378	H	-2.41926131	-4.28083341	4.76607641
C	6.40739069	1.33586898	3.74162418	H	-4.37453797	-4.18917793	6.29545763
C	5.61793237	2.06310364	4.83914702	H	-4.43310051	-5.96583985	6.33016468
C	5.87388604	1.53263478	6.24415602	H	-1.91928849	-6.9621899	0.61142935
O	6.1905983	4.23075285	1.31407228	H	-4.85653234	-5.17331212	1.30806152
O	7.04452549	1.43845752	1.40109416	H	-2.22004963	-5.80918491	7.14486242
O	5.9935055	-0.0278833	3.61778684	H	4.64316697	-5.76275516	4.49983341
O	6.0562438	3.4444787	4.86292216	H	3.22006044	-7.18922785	3.24416628
O	7.25597027	1.52392052	6.57539589	H	1.61800676	-4.80351421	2.23953787
C	1.23212134	6.93353009	3.38363642	H	0.94392283	-6.94251038	4.32320124
C	2.15705894	6.93255476	2.15150132	H	1.45153241	-3.94017961	4.63223425
C	3.07984147	5.71870534	2.11758043	H	1.25855117	-4.37050073	7.03845451
C	3.78670675	5.52395192	3.46297228	H	-0.28050844	-4.6907454	6.21657202
C	2.78697568	5.55999725	4.62622566	H	3.60772676	-6.15352573	1.00551072
C	3.44828976	5.61259339	5.99915425	H	0.28222513	-6.73772138	1.62951938
O	1.3417323	7.01859983	0.99881321	H	1.57789343	-6.66511043	6.98613438
O	4.02798898	5.92926687	1.0787096	H	7.90471994	-0.62453105	4.12382418
O	4.43712973	4.25298959	3.40617625	H	7.79726722	-2.7591982	2.97817322
O	1.9968788	6.77204509	4.55566292	H	4.91187003	-2.12362509	2.26332935
C	-3.98810343	5.71239718	3.47269808	H	5.960311	-4.23040783	4.2011008
C	-3.359338	6.39807722	2.24567951	H	4.49609228	-1.61575375	4.85270934
C	-1.87242684	6.07573117	2.10131223	H	4.06800489	-3.38049551	6.54504747
C	-1.14148616	6.31940068	3.42957324	H	5.80907537	-3.71818251	6.64802843
C	-1.83411325	5.5247297	4.55285286	H	7.16693848	-1.82062239	0.76342995
C	-1.24252992	5.77559533	5.9284131	H	5.14544834	-4.49235518	1.71131158
O	-4.11525091	6.03513381	1.10690384	H	6.01731334	-1.49523256	7.31762651
O	-1.36133862	6.92375476	1.08186781	H	6.2378633	5.16061423	3.84190184
O	0.23149535	5.93781822	3.26093654	H	7.6123357	3.57256147	2.66314993
O	-3.20768397	5.95131197	4.62014999	H	5.13298761	1.89472831	2.0972725
C	-7.09955273	1.36568839	3.45043224	H	7.47240012	1.37846667	3.99838289
C	-7.16577577	2.29954638	2.23077975	H	4.54273364	2.01989835	4.62510535
C	-5.86637791	3.08816873	2.07986649	H	5.29631325	2.14238702	6.95522159
C	-5.47001315	3.75763745	3.4056402	H	5.52251854	0.50257246	6.32338719
C	-5.46967807	2.73856396	4.55573948	H	6.40041851	3.72794503	0.49530977
C	-5.22523119	3.35026884	5.92332448	H	7.00386796	0.45312851	1.48051127
O	-7.46746469	1.49687942	1.10371632	H	7.5589896	2.44345818	6.49842553

H	0.75998009	7.91712623	3.47930259	C	2.34487082	5.91527188	-2.59748994
H	2.78910772	7.82961826	2.23570109	C	1.60401363	6.05567763	-3.93542921
H	2.48377963	4.81878934	1.91947725	C	2.2143714	5.10344306	-4.97682635
H	4.52948023	6.32506537	3.58899604	C	1.5900153	5.22367618	-6.35601091
H	2.12661668	4.68714722	4.57353755	O	4.60752235	5.82490878	-1.63623299
H	4.02073769	4.69930686	6.17195788	O	1.89066589	6.89409479	-1.66668543
H	4.14332461	6.46724264	6.03424447	O	0.22055333	5.76229362	-3.70409631
H	1.86611007	6.94467498	0.16917396	O	3.61095481	5.42820539	-5.11081754
H	4.72691984	5.23277014	1.15702534	O	1.94955822	4.06399146	-7.11155907
H	-4.95742963	6.17405268	3.68821954	C	7.05656369	0.65106658	-3.57563217
H	-3.43539637	7.48307325	2.41616663	C	7.17437379	1.64560756	-2.40889123
H	-1.75005662	5.01669279	1.82742897	C	5.93207278	2.53084787	-2.31301006
H	-1.1979842	7.39000621	3.6711358	C	5.62519993	3.15759135	-3.68074072
H	-1.78335053	4.44985689	4.33823241	C	5.52082242	2.06842272	-4.75975239
H	-3.63806284	6.2463757	0.27519174	C	5.31129765	2.60087461	-6.16547937
H	-0.37511776	6.84920818	1.07207112	O	7.42320367	0.88948725	-1.23879238
H	-8.08792274	0.94550456	3.6555769	O	6.18990265	3.54789404	-1.35011293
H	-7.97973214	3.01814751	2.41120309	O	4.40446676	3.89341513	-3.54106932
H	-5.07057347	2.39183593	1.78903967	O	6.76654514	1.34177007	-4.77729647
H	-6.19505249	4.55303684	3.62771172	O	5.16618602	1.47566852	-7.0291023
H	-4.70486009	1.9757227	4.36826943	C	5.61694	-4.46314728	-3.41486422
H	-4.30097296	3.9391024	5.90215786	C	6.34026164	-3.86835309	-2.18936151
H	-6.06388919	4.02202188	6.16859178	C	6.12664443	-2.36030786	-2.07960048
H	-7.2280176	1.94539171	0.26356401	C	6.38757887	-1.68946549	-3.43115633
H	-5.30012656	4.7023669	1.10162845	C	5.54576815	-2.34224524	-4.53070962
H	-4.83464049	2.63614537	7.69985997	C	5.75005962	-1.73539086	-5.92780962
H	-1.43621551	6.82325272	6.20739884	O	5.89848542	-4.57777064	-1.03856627
H	-0.15864805	5.61553805	5.88658645	O	7.02394388	-1.86237434	-1.08895723
O	-1.86201272	4.87956478	6.8489746	O	6.06766704	-0.30095302	-3.27679353
H	-1.4898508	5.05878919	7.7256989	O	5.91564648	-3.74290389	-4.5803379
C	-5.47549955	4.28933661	-3.98195292	O	7.05416059	-1.22488069	-6.15753504
C	-6.21683184	3.74650858	-2.74814126	C	0.96854968	-7.10131224	-3.35189613
C	-5.98795983	2.24957593	-2.57259294	C	1.81662556	-7.11472466	-2.06764555
C	-6.27049661	1.50476181	-3.87963553	C	2.67611389	-5.85733702	-1.97976518
C	-5.53087954	2.1292221	-5.07199382	C	3.51368614	-5.72410961	-3.25279015
C	-6.00624909	1.58445316	-6.41649317	C	2.62877566	-5.75713689	-4.50861396
O	-5.79559225	4.50922571	-1.62886403	C	3.43670111	-5.87998967	-5.79884553
O	-6.86042155	1.79261845	-1.54723016	O	0.93341324	-7.26728863	-0.97359496
O	-5.84011257	0.15799929	-3.68278137	O	3.51411431	-5.95584834	-0.83536975
O	-5.81268743	3.55220773	-5.13186681	O	4.21542875	-4.48483647	-3.16343295
O	-7.38180751	1.85618647	-6.63795999	O	1.787419	-6.93619127	-4.488741
C	-0.73293331	6.77725965	-3.95392897	C	-4.13125575	-5.50650415	-3.70813874
C	-1.65791858	6.92700676	-2.73494286	C	-3.67560093	-6.3079912	-2.47682623
C	-2.57431028	5.71579672	-2.59801925	C	-2.17646252	-6.14495274	-2.23482931
C	-3.32217152	5.47127169	-3.91377843	C	-1.37940554	-6.41248979	-3.52118073
C	-2.3298046	5.35930768	-5.08678193	C	-1.92531719	-5.56429745	-4.68022418
C	-3.0264398	5.30537393	-6.43522948	C	-1.27481585	-5.86036101	-6.01999262
O	-0.84649202	7.1388742	-1.59639256	O	-4.47337859	-5.88189434	-1.38996693
O	-3.48875658	5.97254563	-1.54286191	O	-1.7959054	-7.06455935	-1.21820315
O	-4.07779986	4.26861432	-3.75022582	O	-0.00908252	-6.08410334	-3.2616292
O	-1.48819042	6.52747603	-5.1150673	O	-3.32997643	-5.84337511	-4.8326598
O	-2.06423045	4.95320931	-7.4303708	C	-6.73020669	-0.89991799	-3.98521243
C	4.38173092	5.24392045	-3.9444765	C	-6.91778733	-1.78211399	-2.73251437
C	3.84507187	6.1194011	-2.79424825	C	-5.68198769	-2.63695355	-2.44390111

C	-5.24979338	-3.34848891	-3.72962027	H	5.9701963	-5.48256925	-3.59782973
C	-5.00119593	-2.32285404	-4.84026649	H	7.41660002	-4.03795563	-2.33846959
C	-4.51864437	-2.92510406	-6.16653746	H	5.08659045	-2.15873135	-1.78866445
O	-7.27562937	-0.9324337	-1.65395746	H	7.44333336	-1.80032999	-3.70100181
O	-6.03714272	-3.57752442	-1.43497591	H	4.4809754	-2.27478246	-4.28256482
O	-4.08036767	-4.13048784	-3.4340964	H	5.55824837	-2.53504584	-6.65460679
O	-6.2626596	-1.65748168	-5.07581344	H	4.99876013	-0.95471892	-6.08349897
O	-5.07553969	-4.19526591	-6.46951361	H	6.14718757	-4.10093088	-0.21672606
H	-5.80053069	5.3159542	-4.183816	H	7.04465366	-0.87717072	-1.163393
H	-7.2915286	3.90442306	-2.92364373	H	6.98248348	-0.25628094	-6.08366259
H	-4.94011138	2.07280919	-2.29588422	H	0.48375638	-8.07466929	-3.48396622
H	-7.34838801	1.5349174	-4.08142503	H	2.49001143	-7.98314708	-2.13038961
H	-4.45153803	1.96855231	-4.9649679	H	2.01418804	-4.98304898	-1.89454751
H	-5.37988886	2.01519633	-7.21380257	H	4.2247427	-6.55795146	-3.30335871
H	-5.8899988	0.49830364	-6.43335678	H	2.00632194	-4.85363555	-4.54019456
H	-6.09802414	4.1036398	-0.78408017	H	2.74582509	-5.82084438	-6.65486143
H	-6.88251095	0.80315418	-1.58088512	H	1.37874451	-7.06386369	-0.12001219
H	-7.48569464	2.81470336	-6.51808811	H	4.30277057	-5.37007109	-0.96398678
H	-0.22234762	7.72695411	-4.1454423	H	-5.14570126	-5.80475009	-3.98679935
H	-2.29082797	7.80925536	-2.91537676	H	-3.8641896	-7.37113681	-2.69182388
H	-1.96130195	4.82985119	-2.37549489	H	-1.98474842	-5.11113123	-1.91721218
H	-4.00150246	6.31737065	-4.09217708	H	-1.46792415	-7.47796272	-3.77624639
H	-1.71897981	4.45664495	-4.95824838	H	-1.78209749	-4.50149034	-4.44933161
H	-3.8400737	4.57081692	-6.38992477	H	-4.09231347	-6.13144878	-0.51954994
H	-3.45753248	6.29671824	-6.64077135	H	-0.80919195	-7.05436824	-1.14415362
H	-1.36006453	7.03881138	-0.76440435	H	-7.69692696	-0.49684061	-4.30285439
H	-4.24614972	5.33703625	-1.60626222	H	-7.74413385	-2.4756901	-2.94851174
H	-2.49191723	5.04450284	-8.29527058	H	-4.85998113	-1.99172964	-2.10009306
H	5.39087569	5.57402606	-4.21285968	H	-6.0539368	-4.01135528	-4.06681483
H	4.00073106	7.16897682	-3.08690111	H	-4.26603996	-1.57624359	-4.51202172
H	2.1752108	4.9028431	-2.20737918	H	-4.80919978	-2.22714873	-6.96090654
H	1.70891006	7.08952927	-4.29310781	H	-3.42430569	-2.97780796	-6.15296946
H	2.1058925	4.06639305	-4.63552289	H	-7.19752476	-1.39514529	-0.79007986
H	0.50220012	5.30894128	-6.26375118	H	-5.35697099	-4.2917036	-1.42016531
H	1.97191895	6.14003626	-6.83193102	H	-4.40707058	-4.85330707	-6.20720394
H	4.14697787	6.10881555	-0.81785281	H	4.1483499	-5.05397844	-5.86736218
H	0.90186277	6.88516943	-1.66417037	H	-1.66898097	-6.81483478	-6.39613625
H	1.46632904	4.11153374	-7.95018251	H	-0.1906995	-5.95837612	-5.88808482
H	8.02003877	0.16353118	-3.74959614	O	-1.59234001	-4.78328205	-6.90455626
H	8.03206078	2.30159067	-2.62255148	H	-1.52854984	-5.10505358	-7.81560141
H	5.07080838	1.91761772	-2.01084244	H	0.49684838	-3.0722082	-7.11621266
H	6.44296103	3.84371614	-3.9427543	H	0.00022511	2.02359643	-7.1089469
H	4.69975035	1.38465616	-4.51405601	H	-2.09628883	-1.22461418	8.18393592
H	4.42161514	3.23915965	-6.18518281	H	-0.23970955	2.97973799	7.23587001
H	6.18481106	3.20797943	-6.45411132	O	2.47862837	5.71152874	7.03404318
H	7.26776849	1.41577229	-0.42398086	H	1.93578071	6.48370191	6.80393437
H	5.51111858	4.2567009	-1.45783264	O	4.19364251	-7.07965926	-5.8292084
H	4.78367476	1.78974985	-7.86116431	H	3.55720759	-7.79933159	-5.68398406

5. ^{1}H NMR, ^{13}C NMR, and ESI-MS spectra of new compounds

^{1}H NMR (500 MHz, $CDCl_3$) of **S2**.

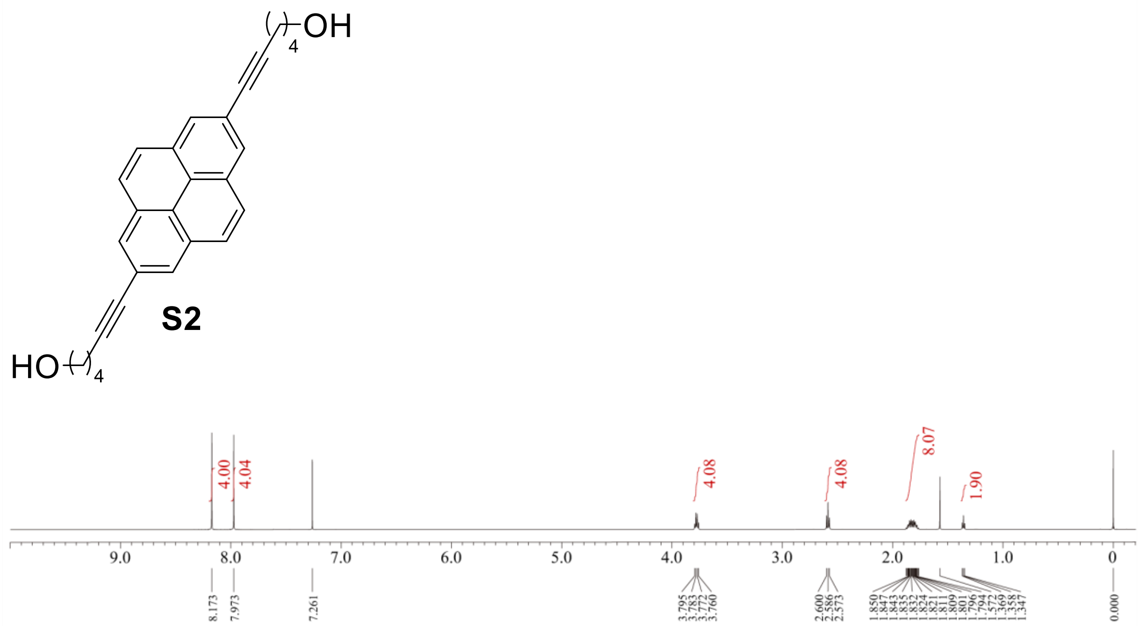

^{13}C NMR (125 MHz, $CDCl_3$) of **S2**.

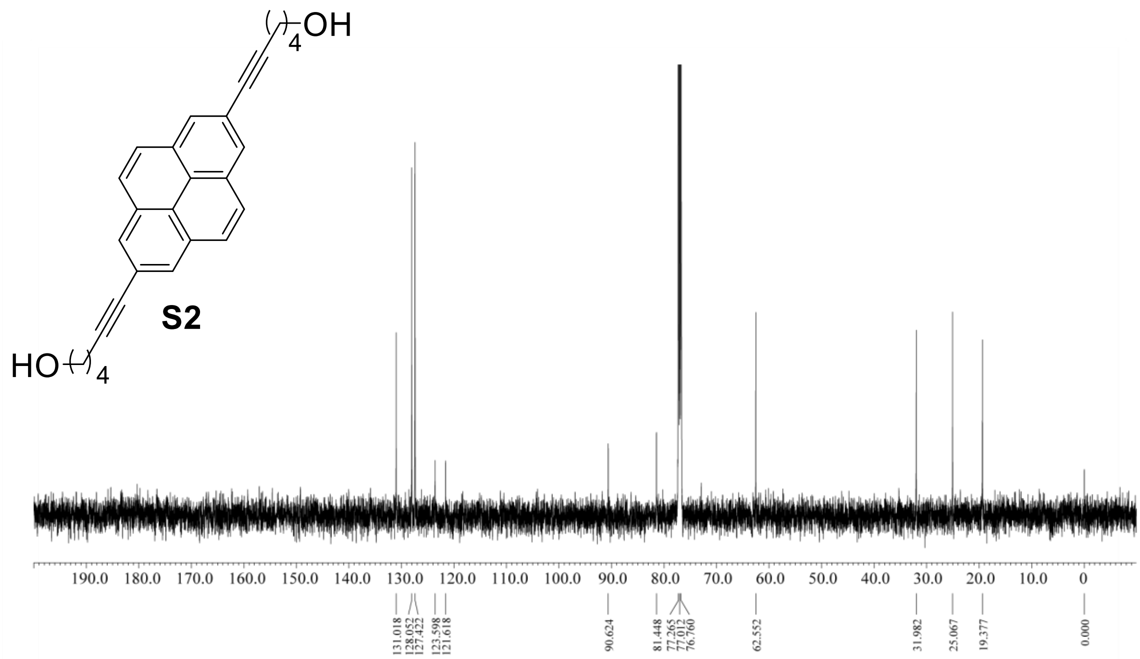

ESI-MS spectra of **S2**.

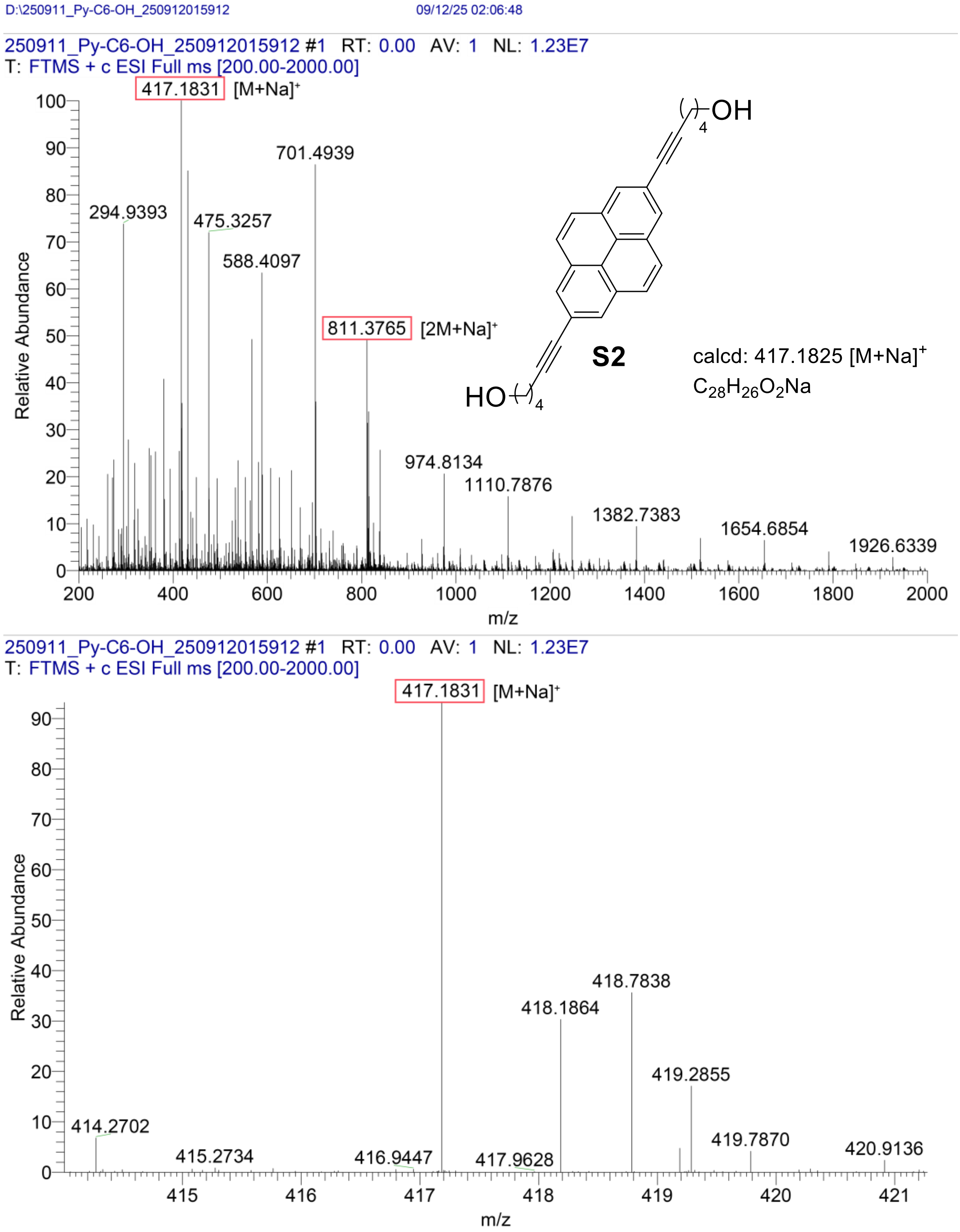

^{1}H NMR (500 MHz, $CDCl_3$) of **S4**.

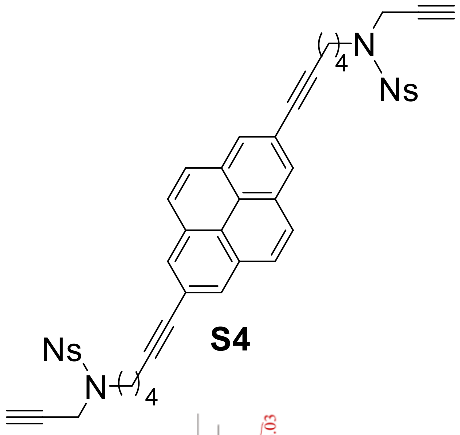

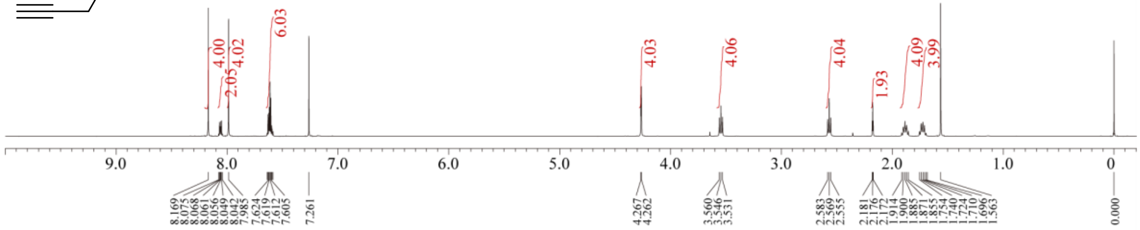

^{13}C NMR (125 MHz, $CDCl_3$) of **S4**.

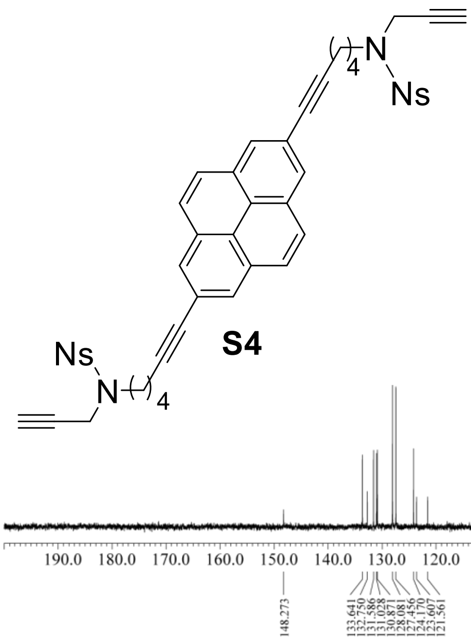

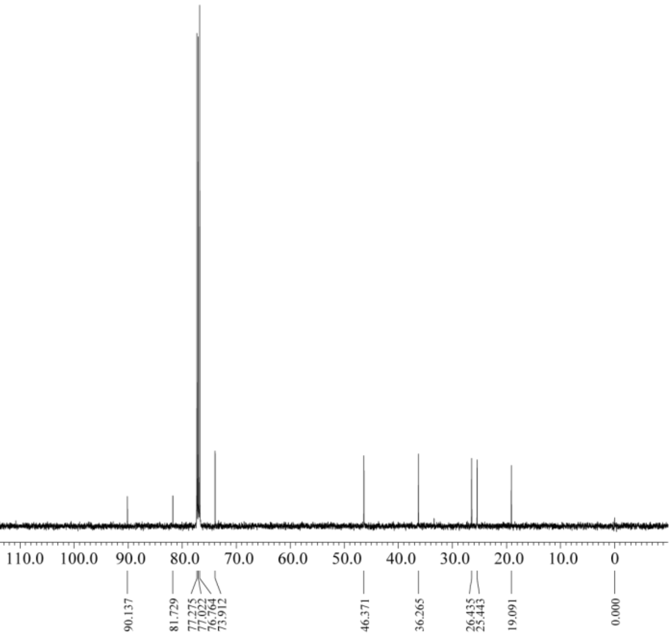

ESI-MS spectra of **S4**.

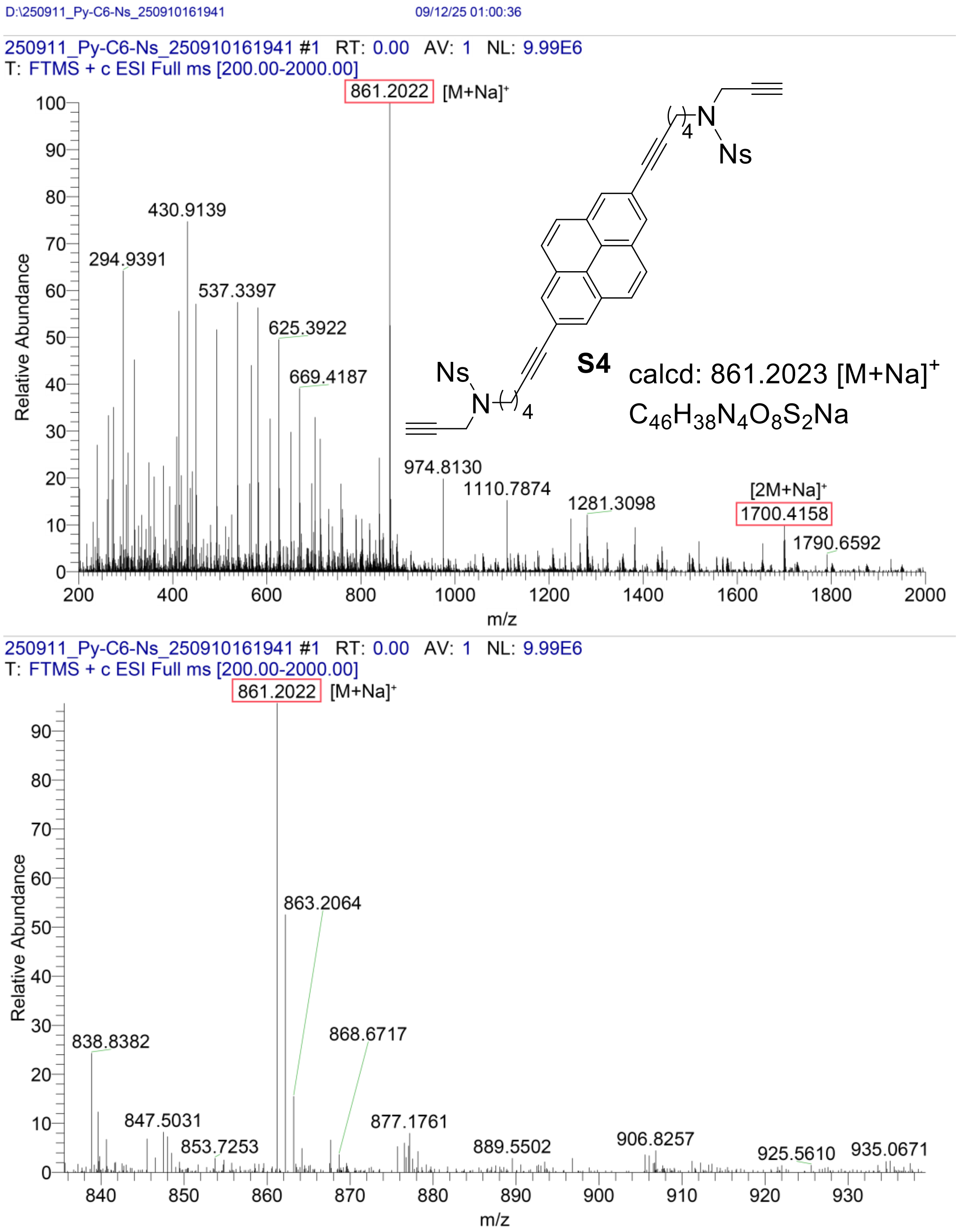

^{1}H NMR (500 MHz, CD_3OD) of **Axis-C6**.

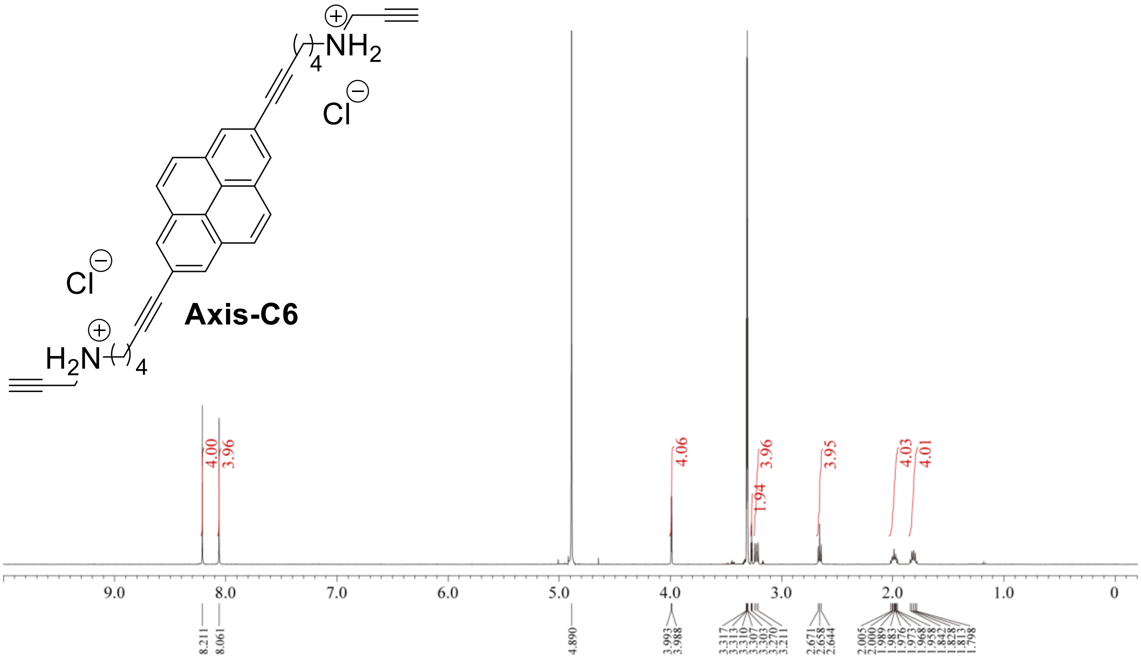

^{13}C NMR (125 MHz, CD_3OD) of **Axis-C6**.

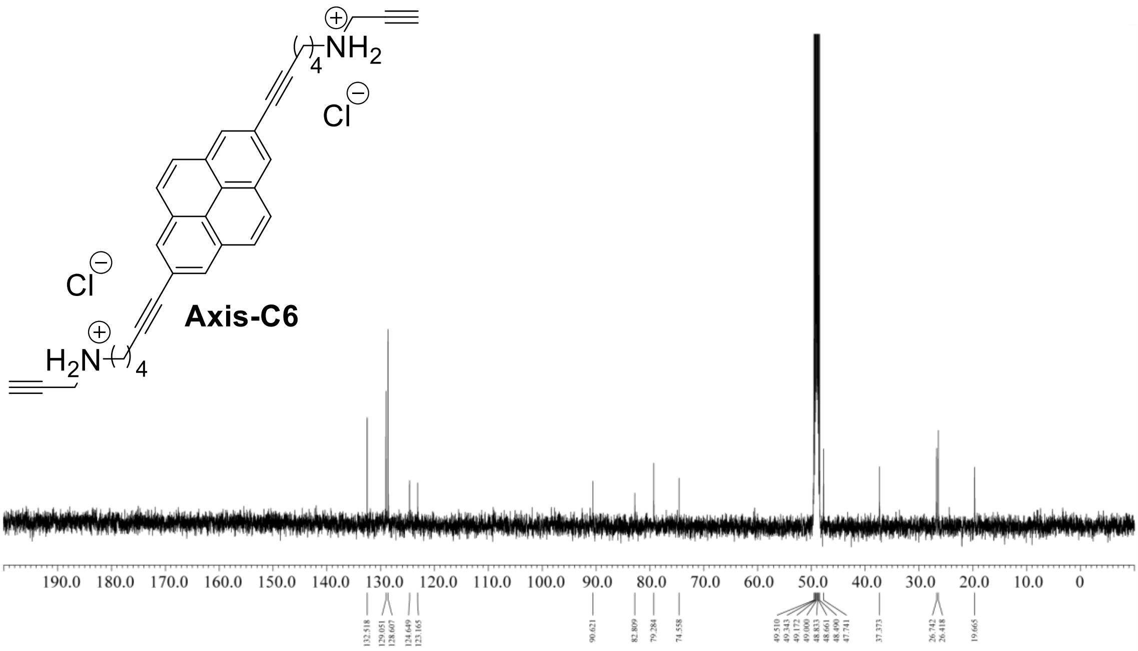

ESI-MS spectra of **Axis-C6**.

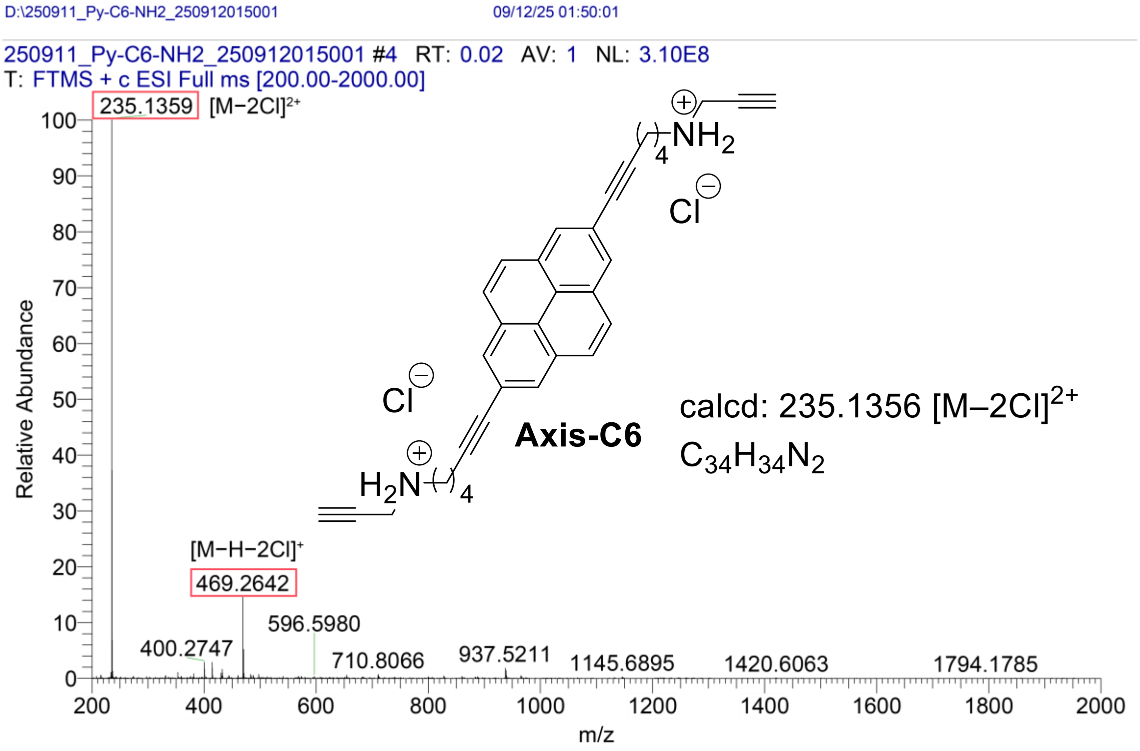

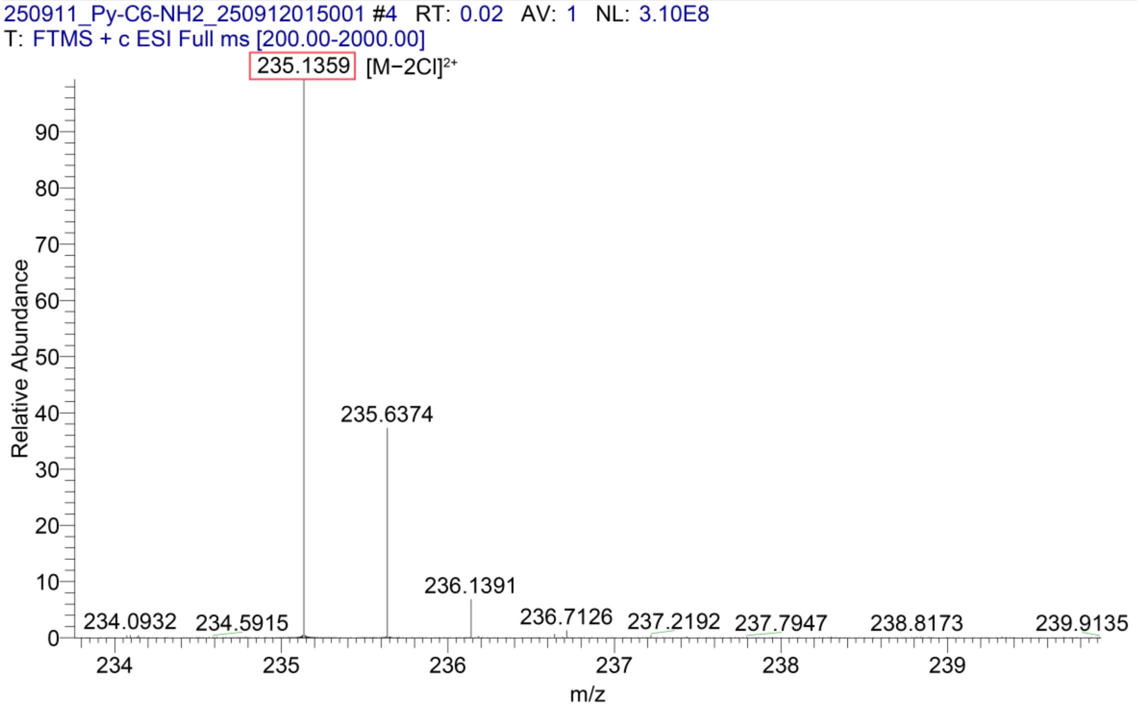

^{1}H NMR (500 MHz, D_2O) of **Rota-C6**.

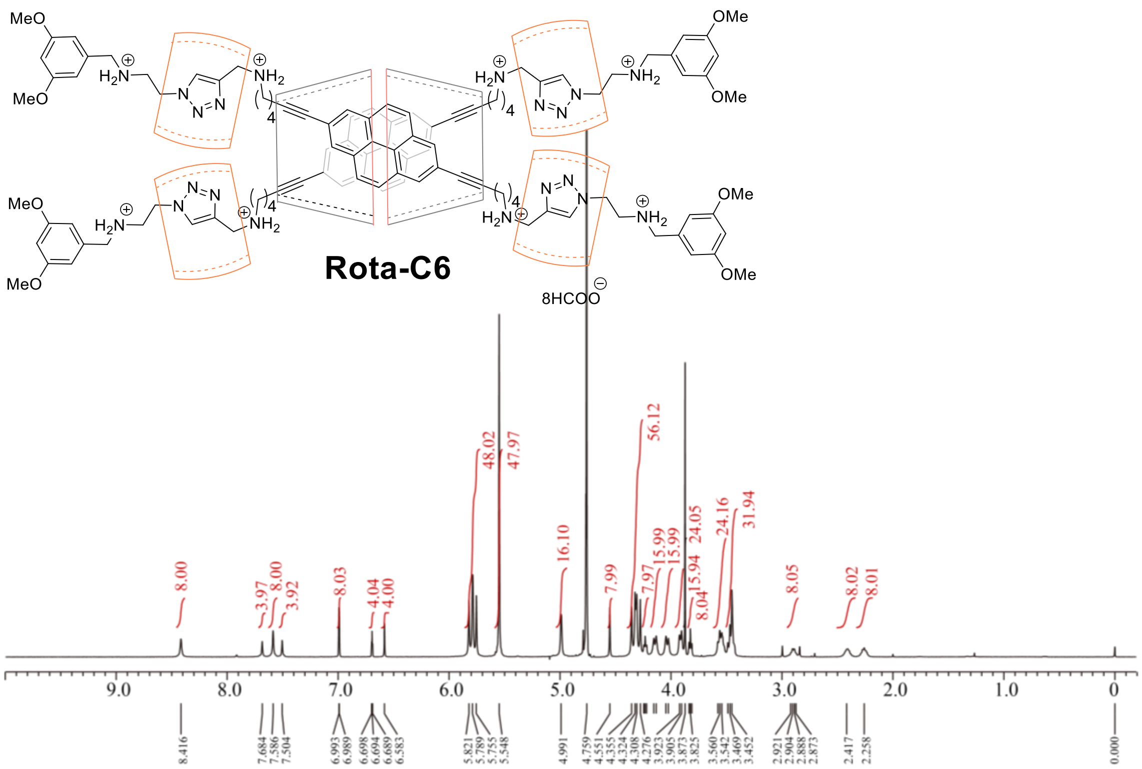

^{13}C NMR (125 MHz, D_2O) of **Rota-C6**.

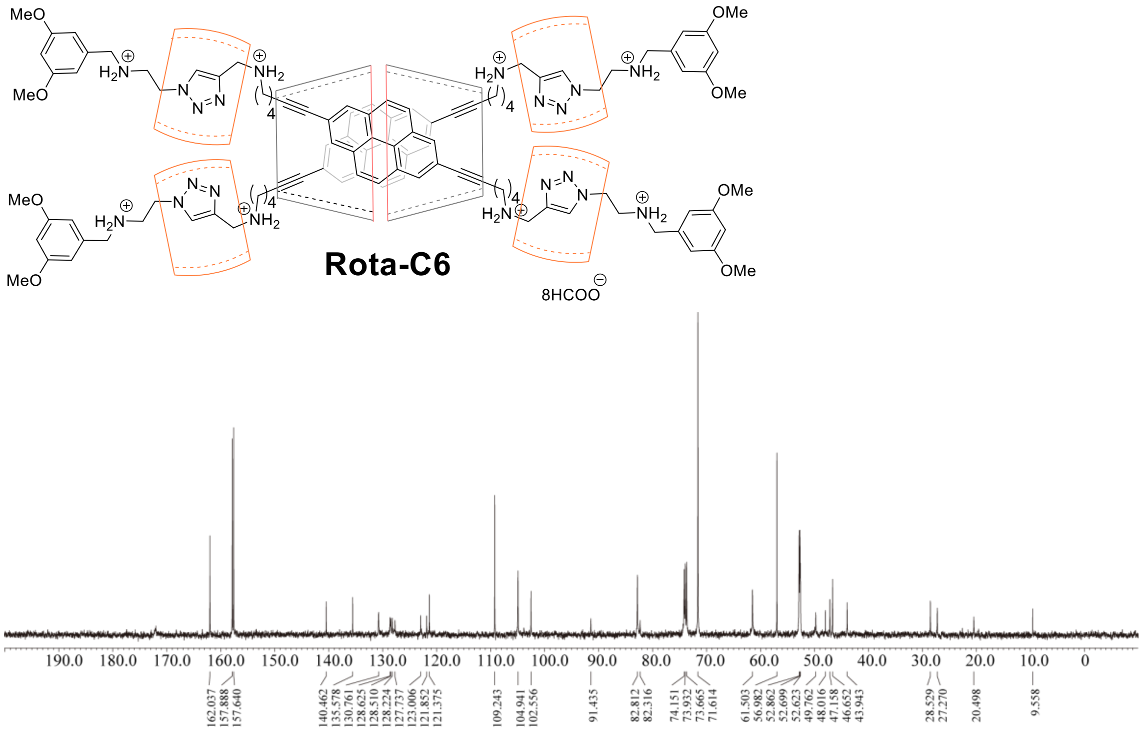

ESI-MS spectra of **Rota-C6**.

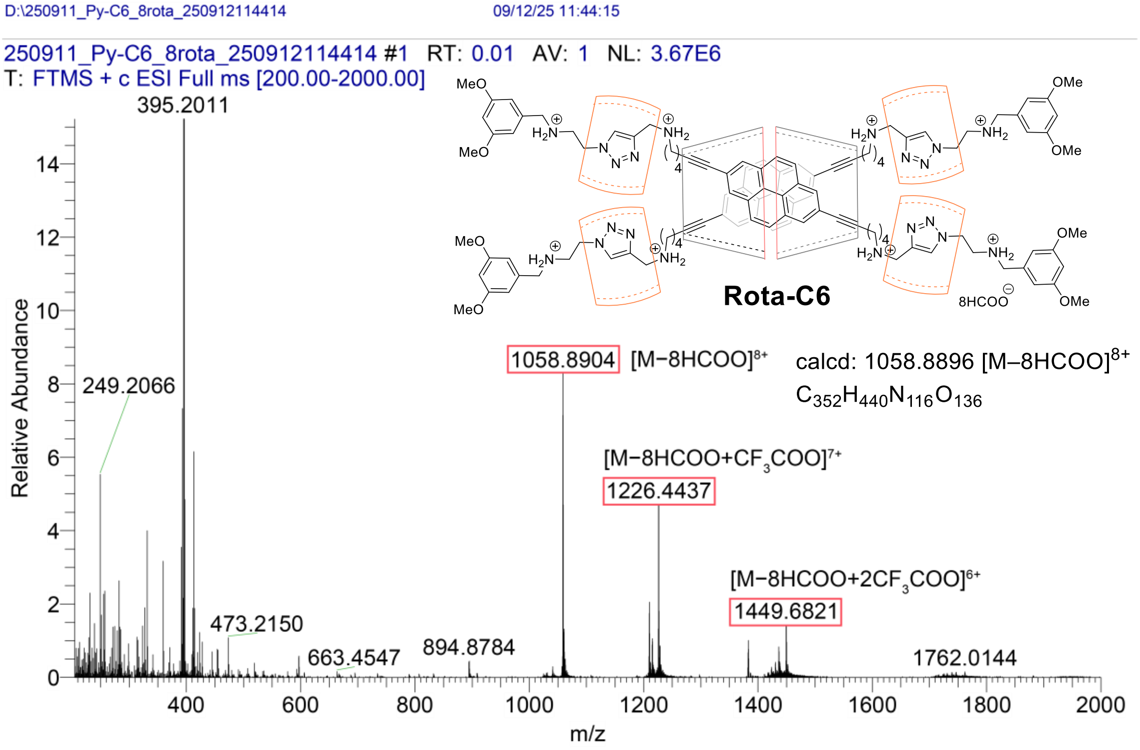

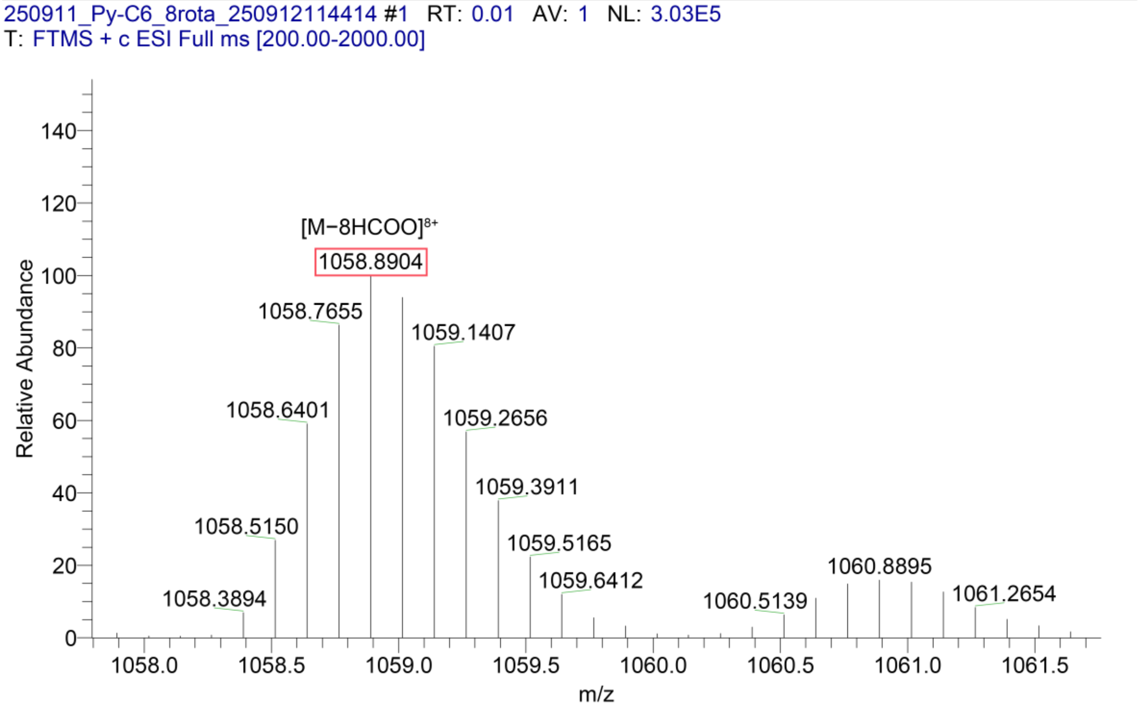

^{1}H NMR (500 MHz, $CDCl_3$) of **S5**.

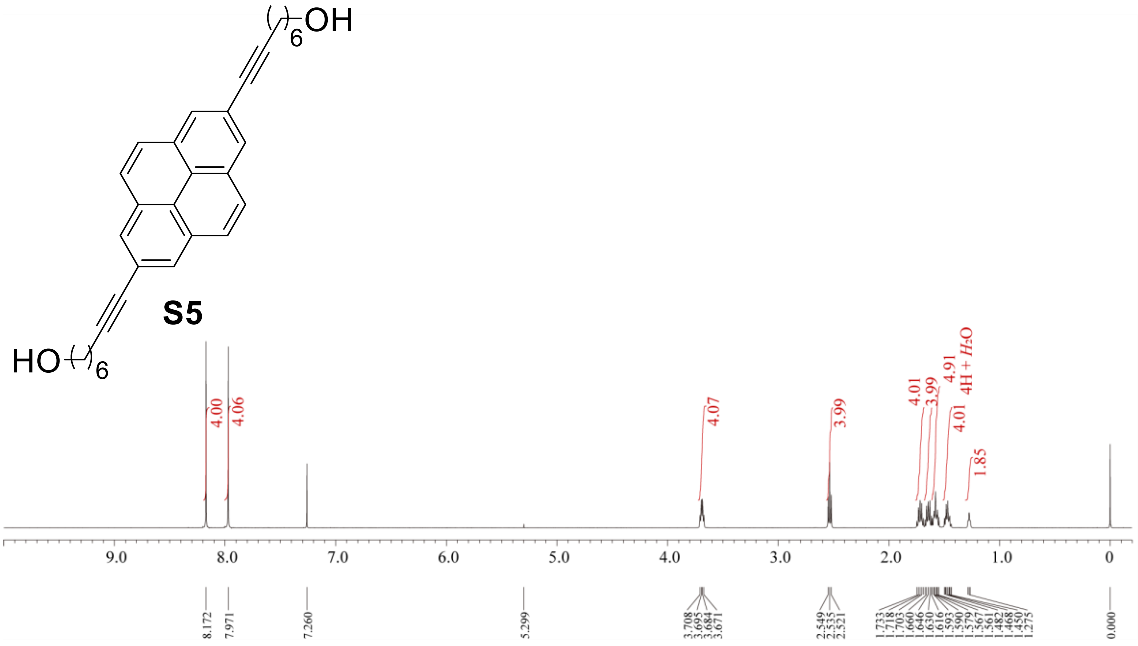

^{13}C NMR (125 MHz, $CDCl_3$) of **S5**.

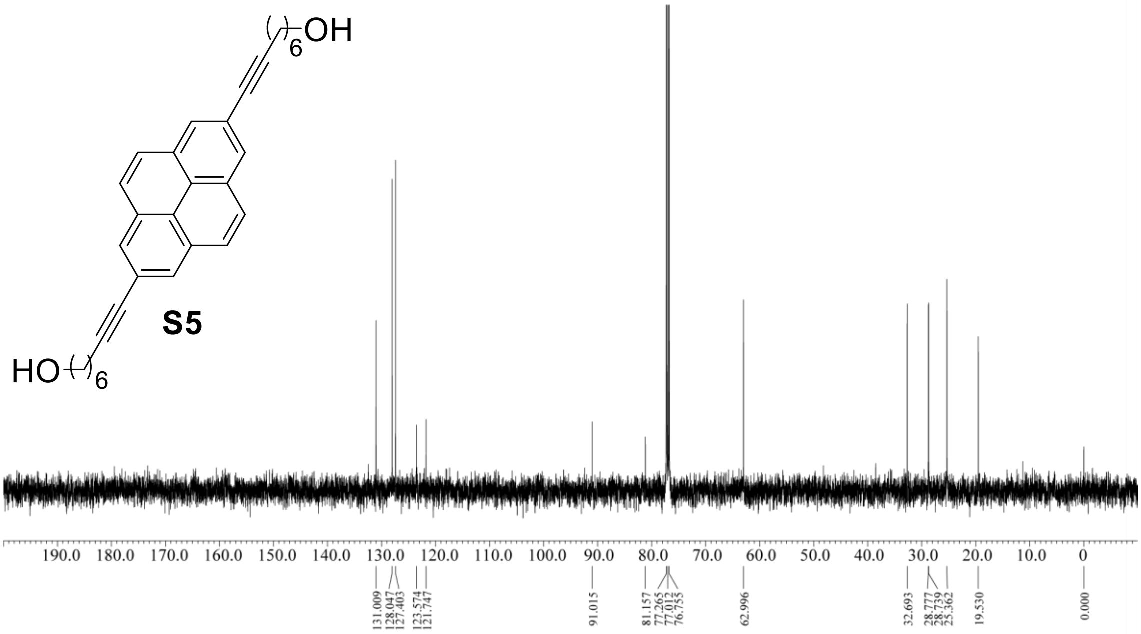

ESI-MS spectra of **S5**.

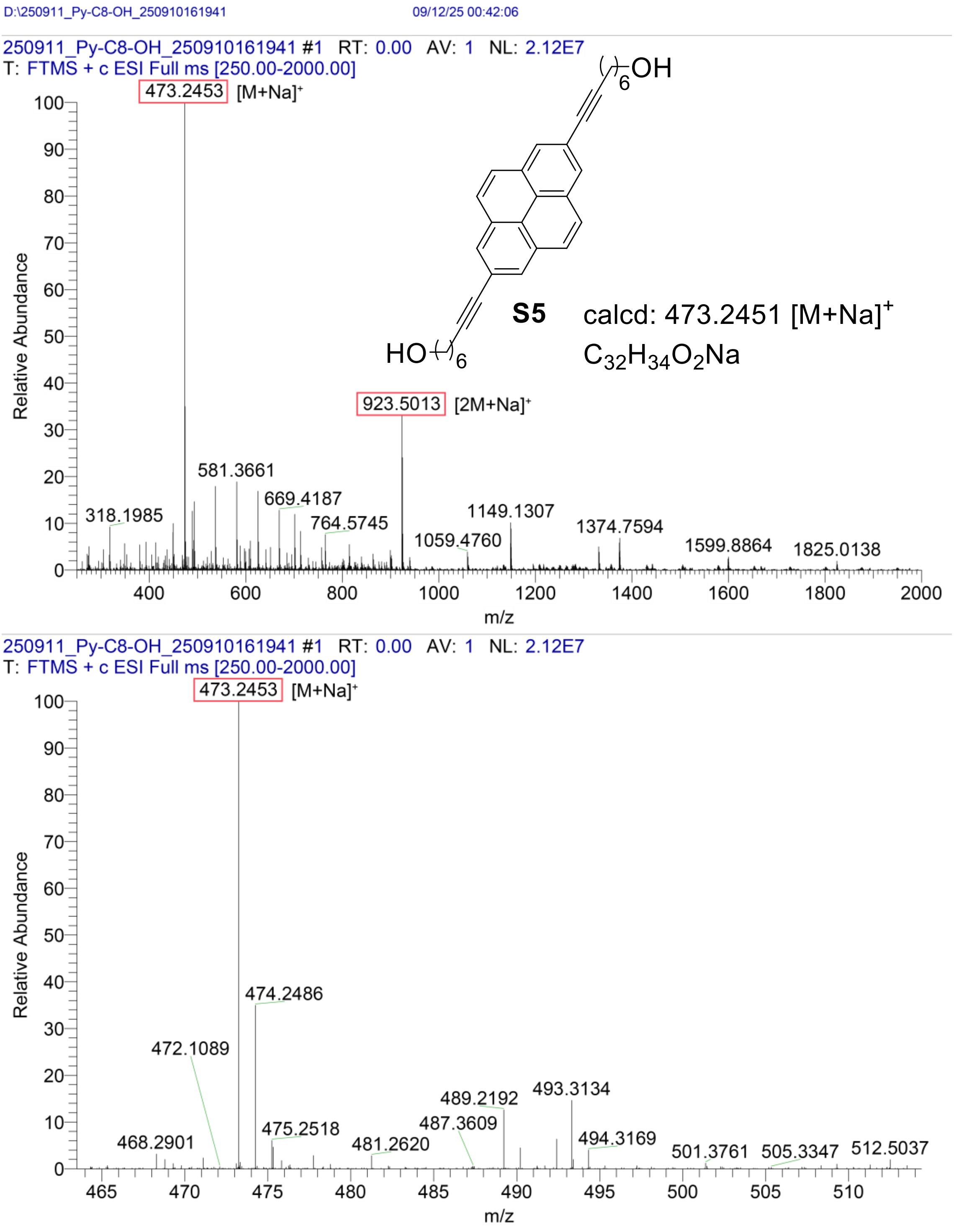

^{1}H NMR (500 MHz, $CDCl_3$) of **S6**.

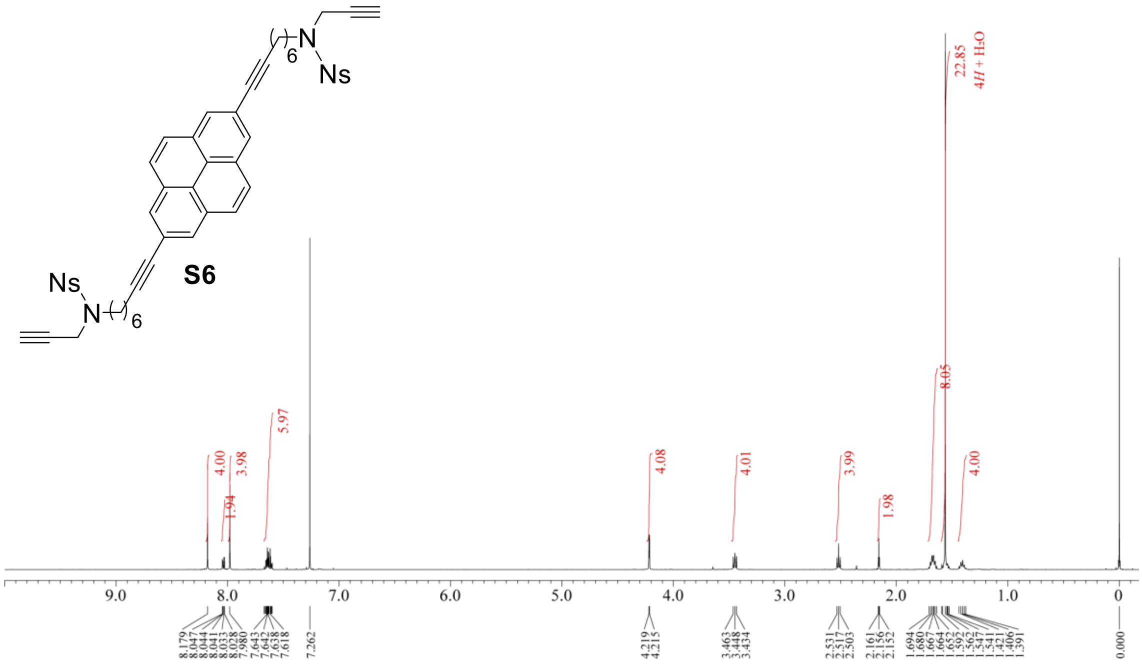

^{13}C NMR (125 MHz, $CDCl_3$) of **S6**.

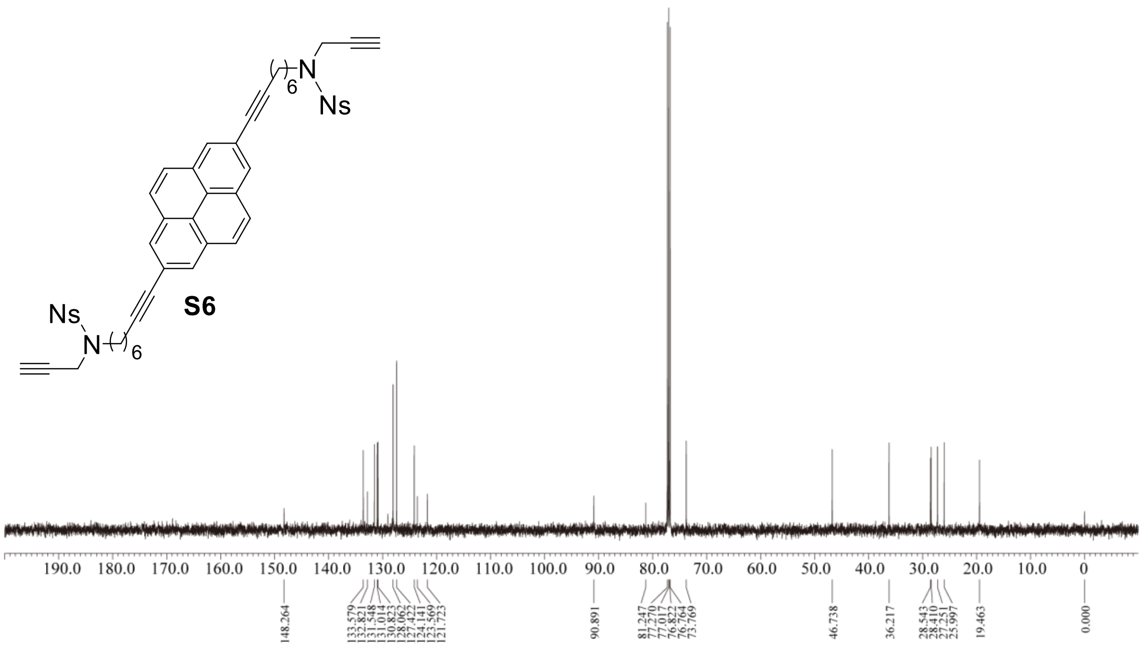

ESI-MS spectra of **S6**.

D:\250911_Py-C8-Ns_250912010304 09/12/25 01:03:40

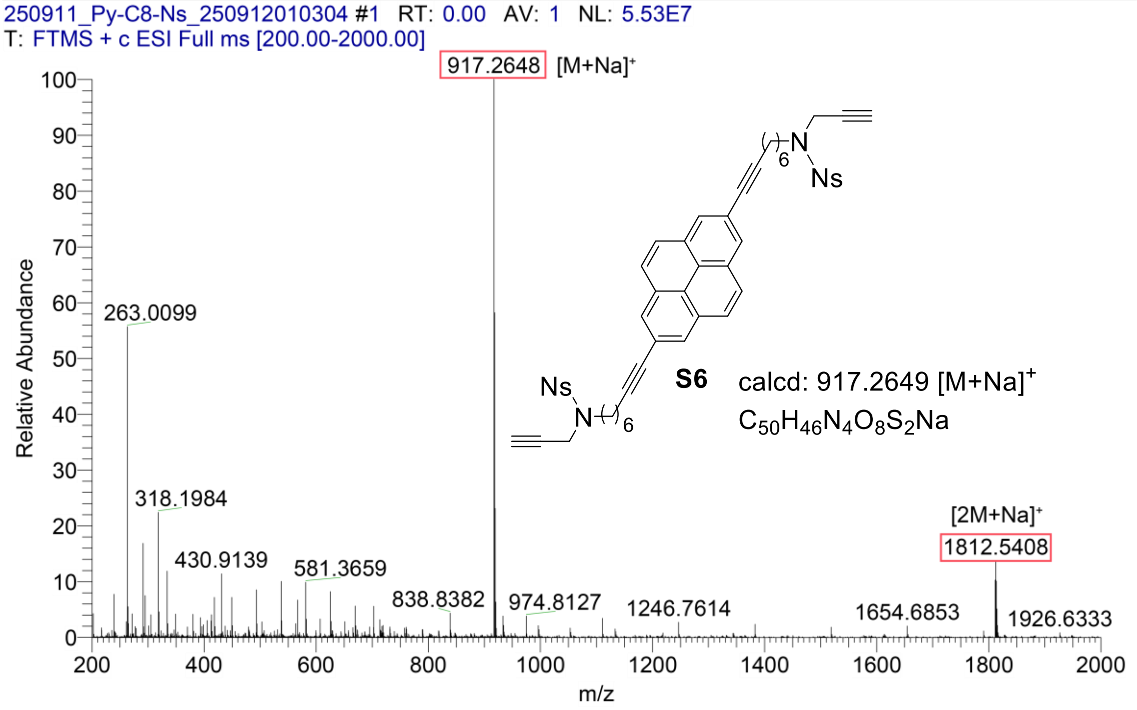

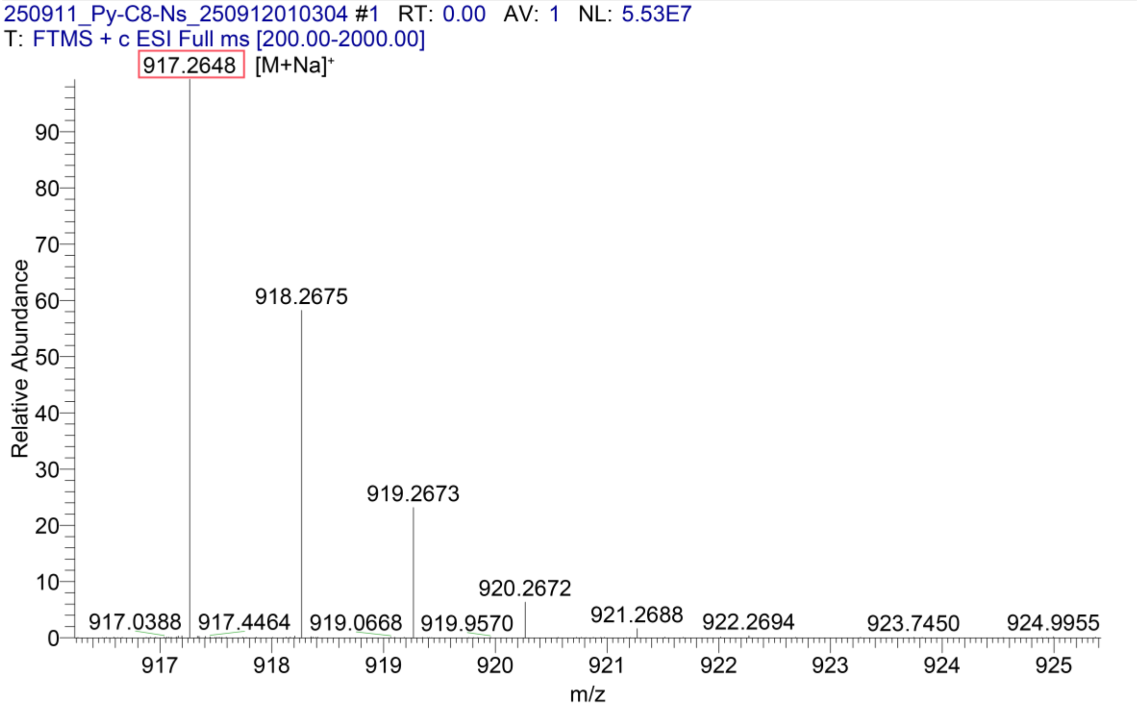

^{1}H NMR (500 MHz, CD_3OD) of **Axis-C8**.

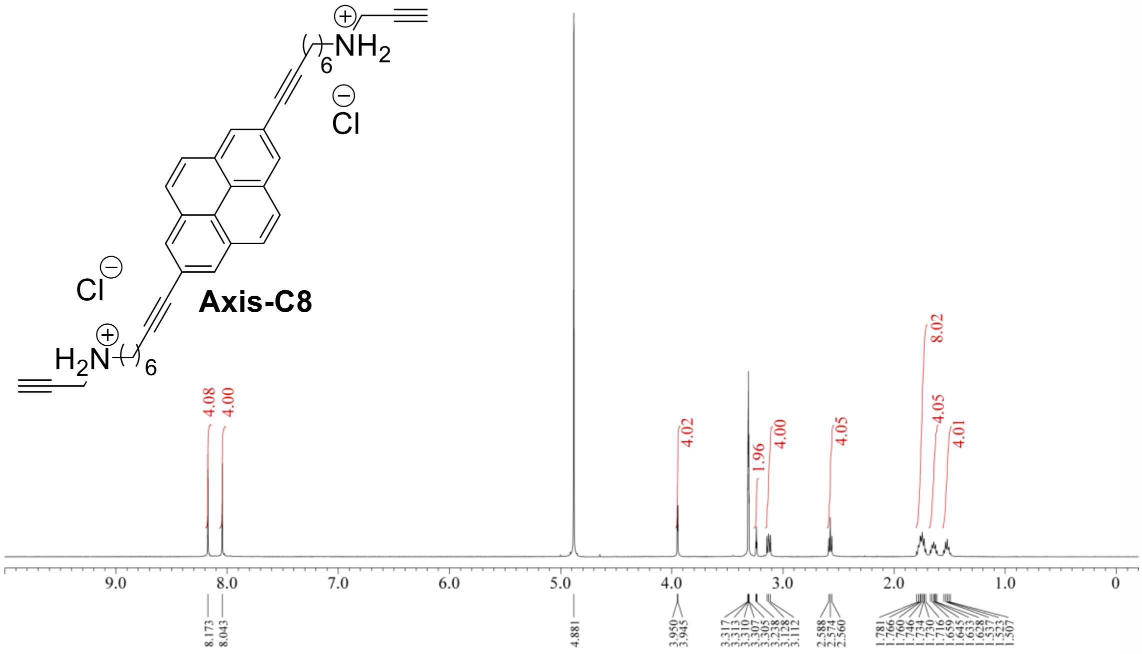

^{13}C NMR (125 MHz, CD_3OD) of **Axis-C8**.

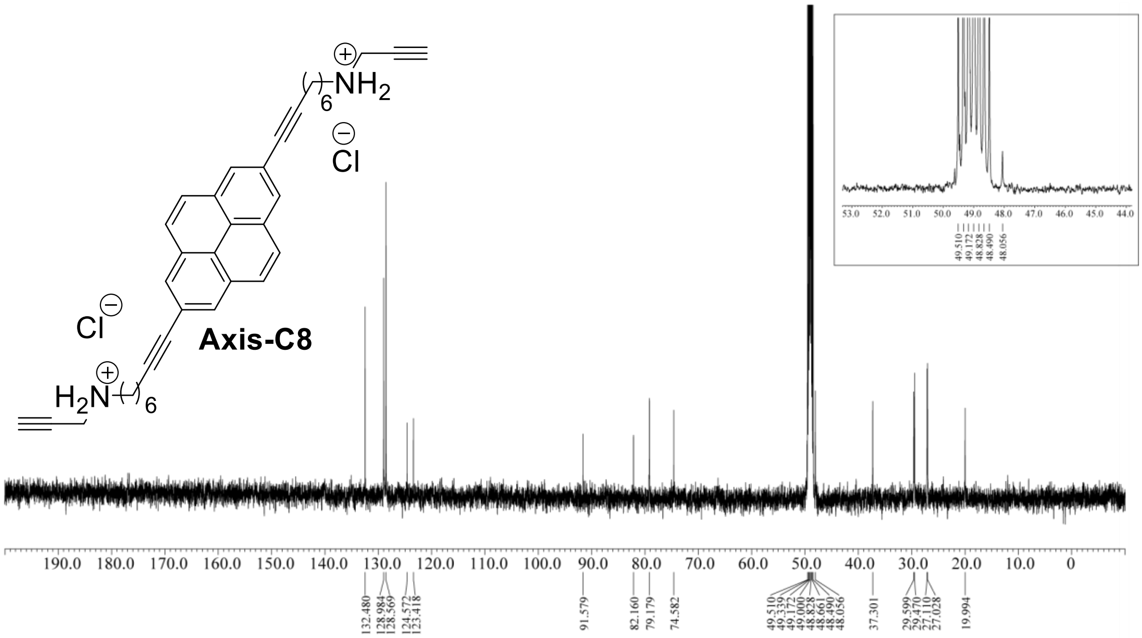

ESI-MS spectra of **Axis-C8**.

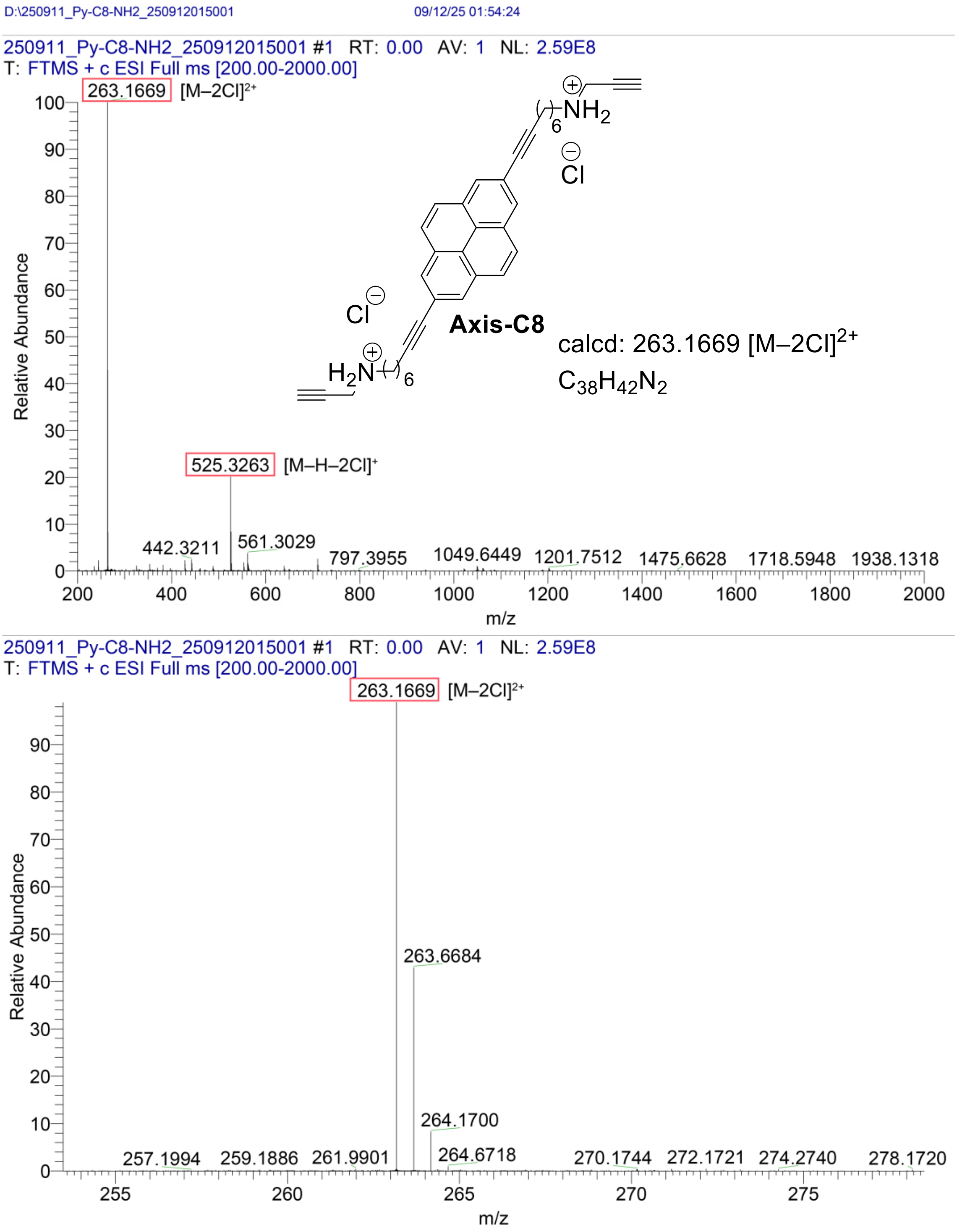

^{1}H NMR (500 MHz, D_2O) of **Rota-C8**.

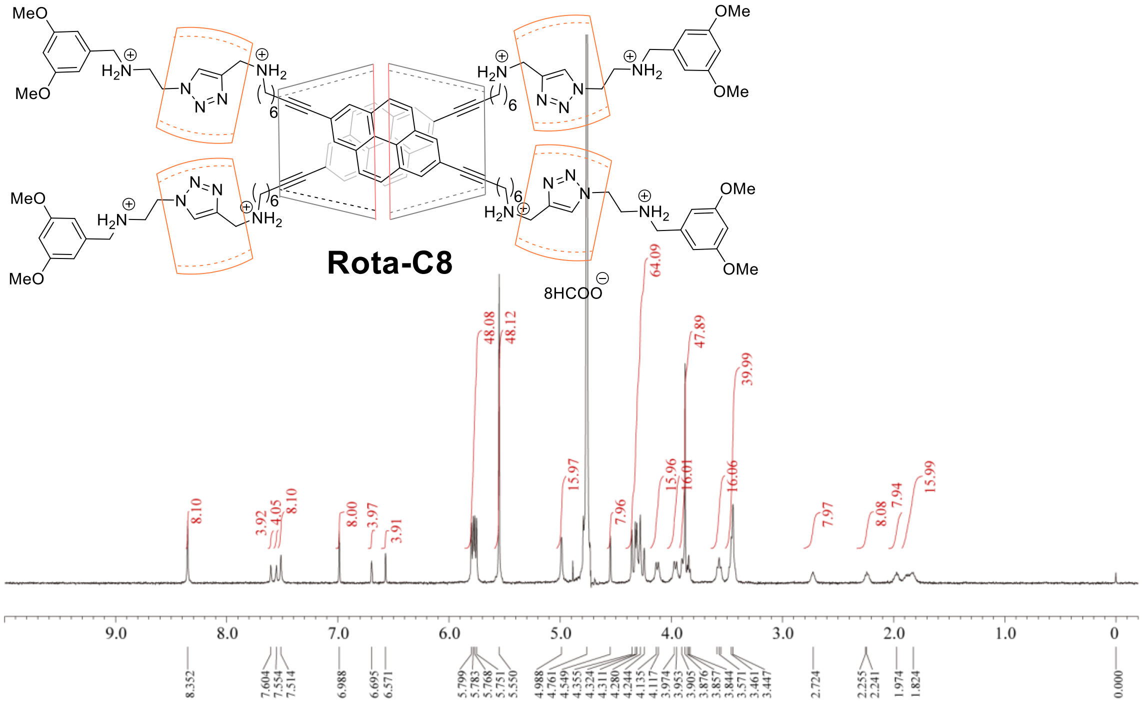

^{13}C NMR (125 MHz, D_2O) of **Rota-C8**.

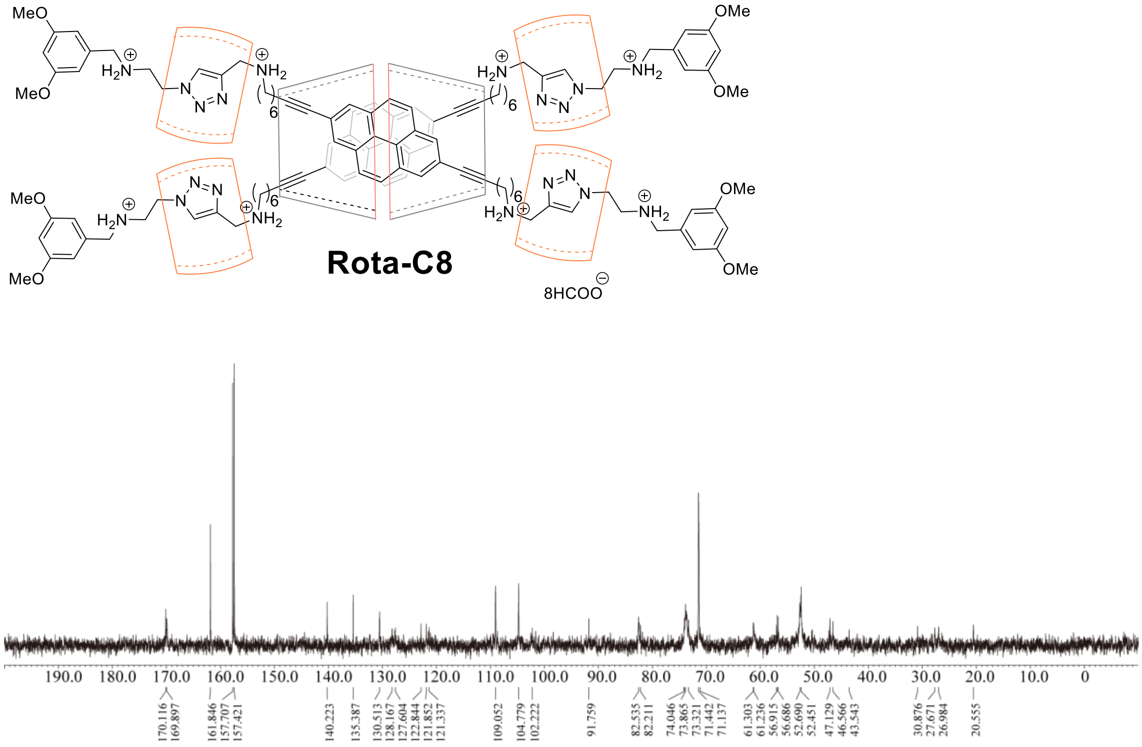

ESI-MS spectra of **Rota-C8**.

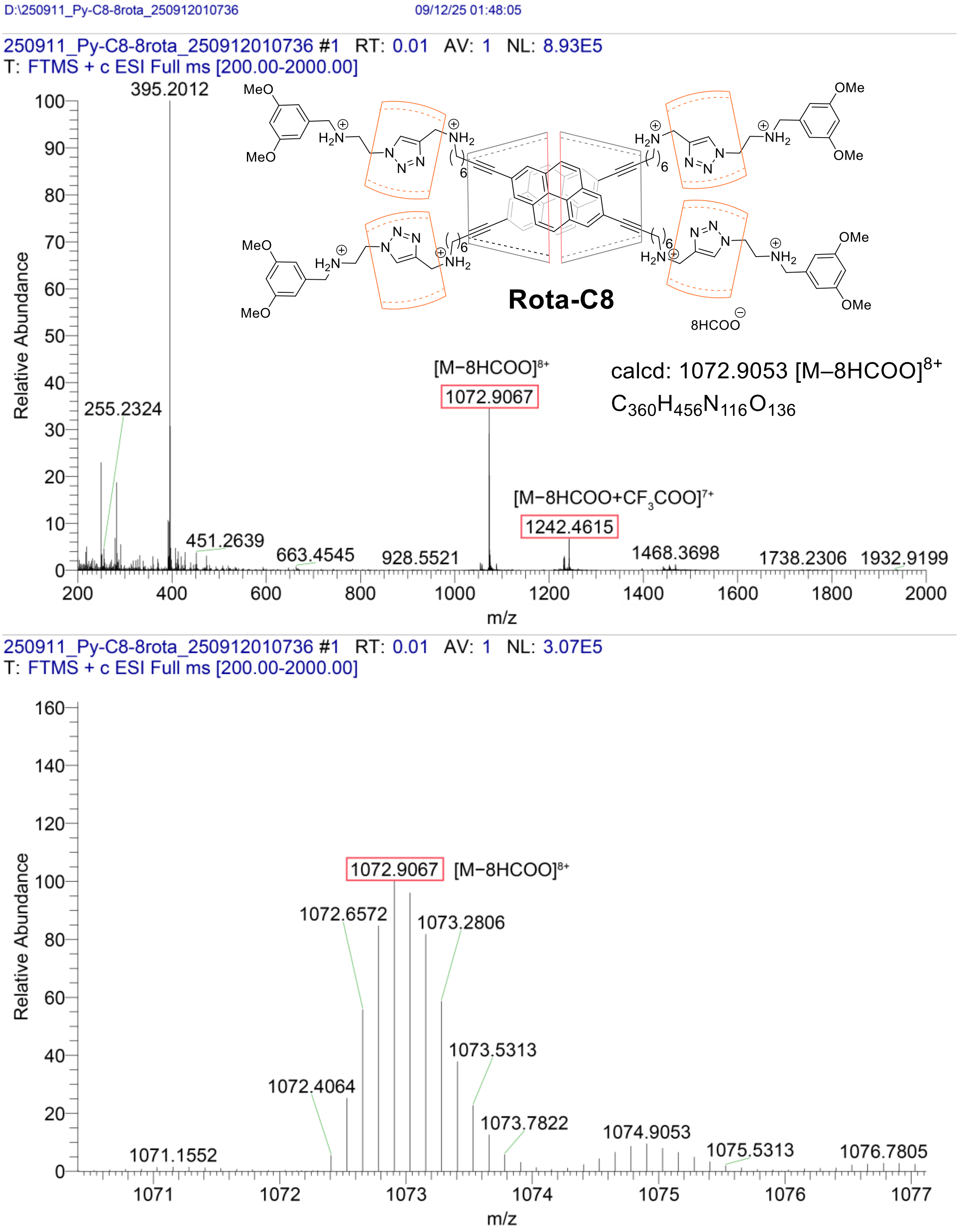

^{1}H NMR (500 MHz, $CDCl_3$) of **S7**.

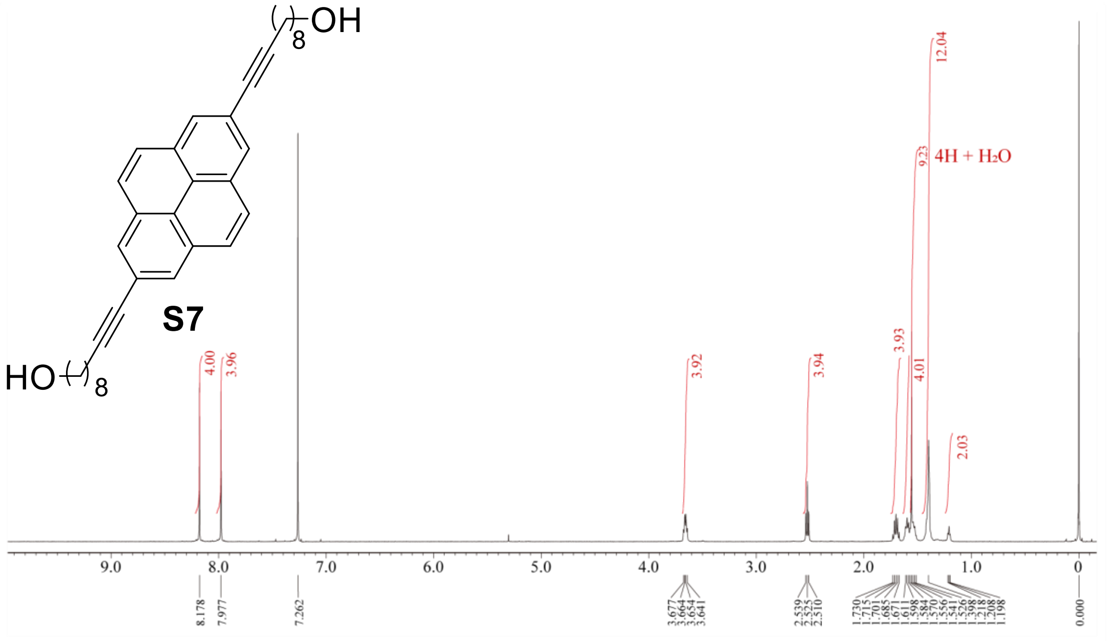

^{13}C NMR (125 MHz, $CDCl_3$) of **S7**.

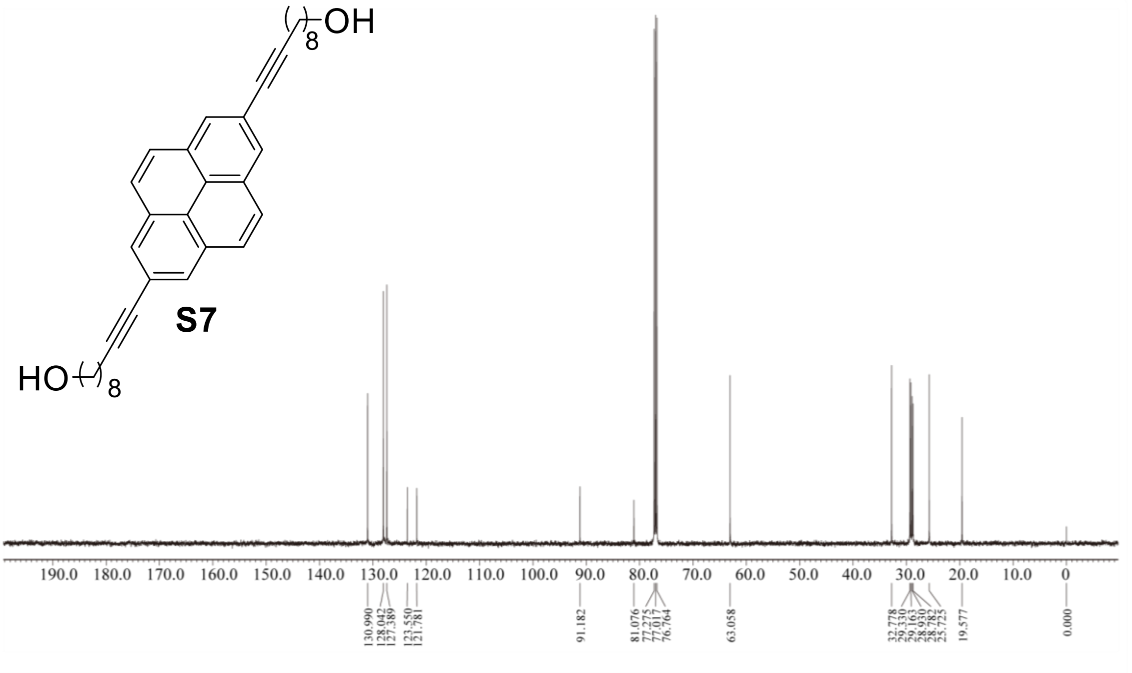

ESI-MS spectra of **S7**.

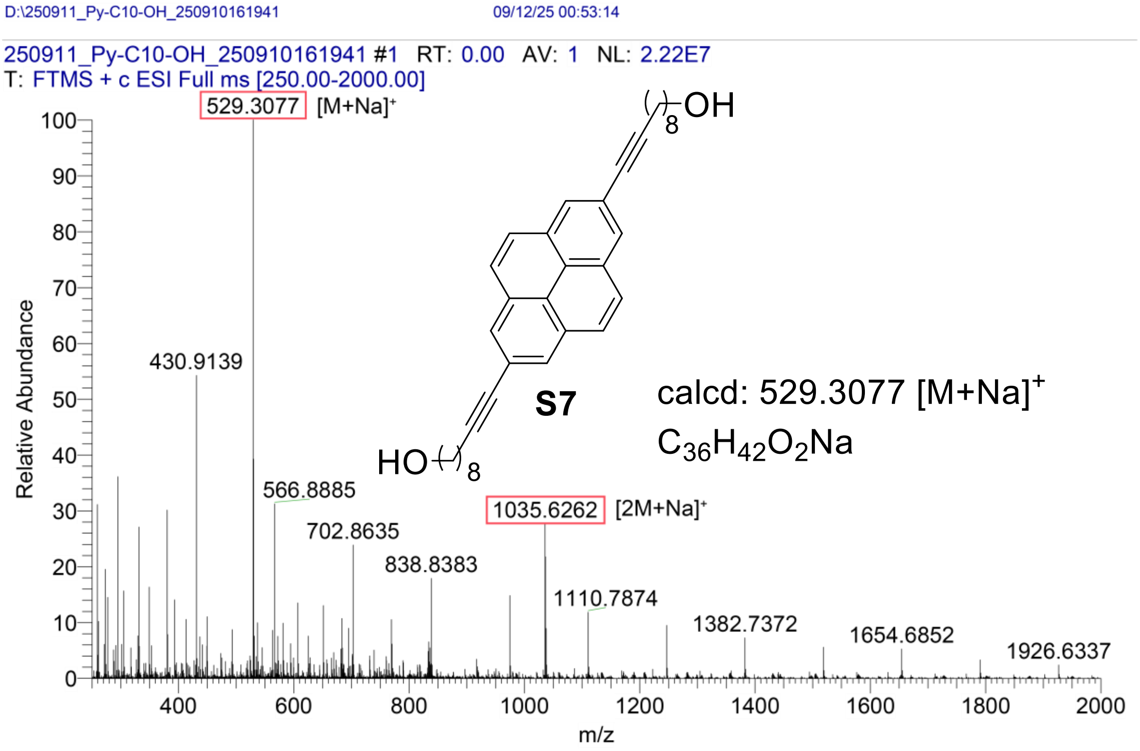

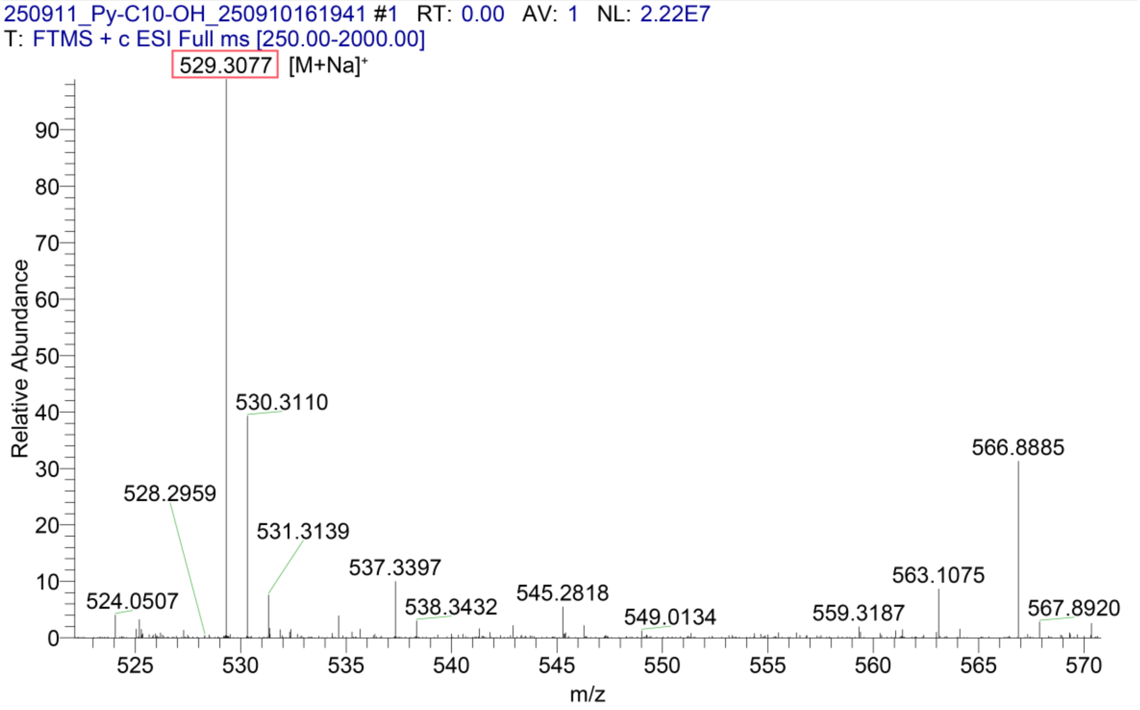

^{1}H NMR (500 MHz, $CDCl_3$) of **S8**.

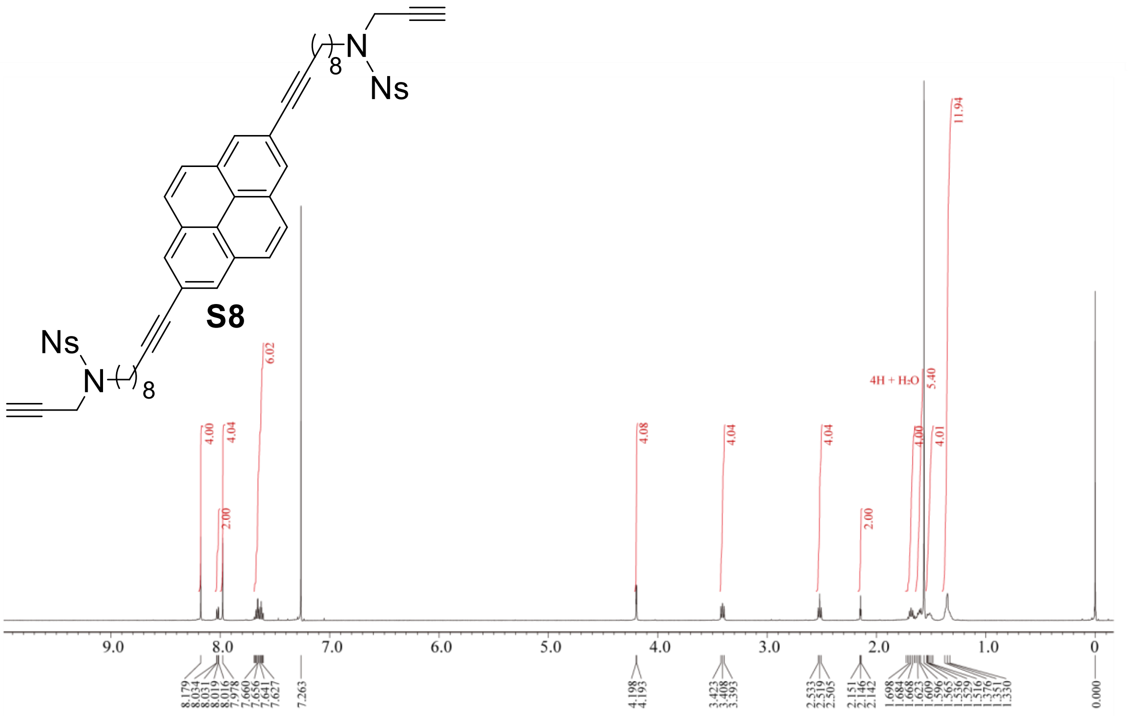

^{13}C NMR (125 MHz, $CDCl_3$) of **S8**.

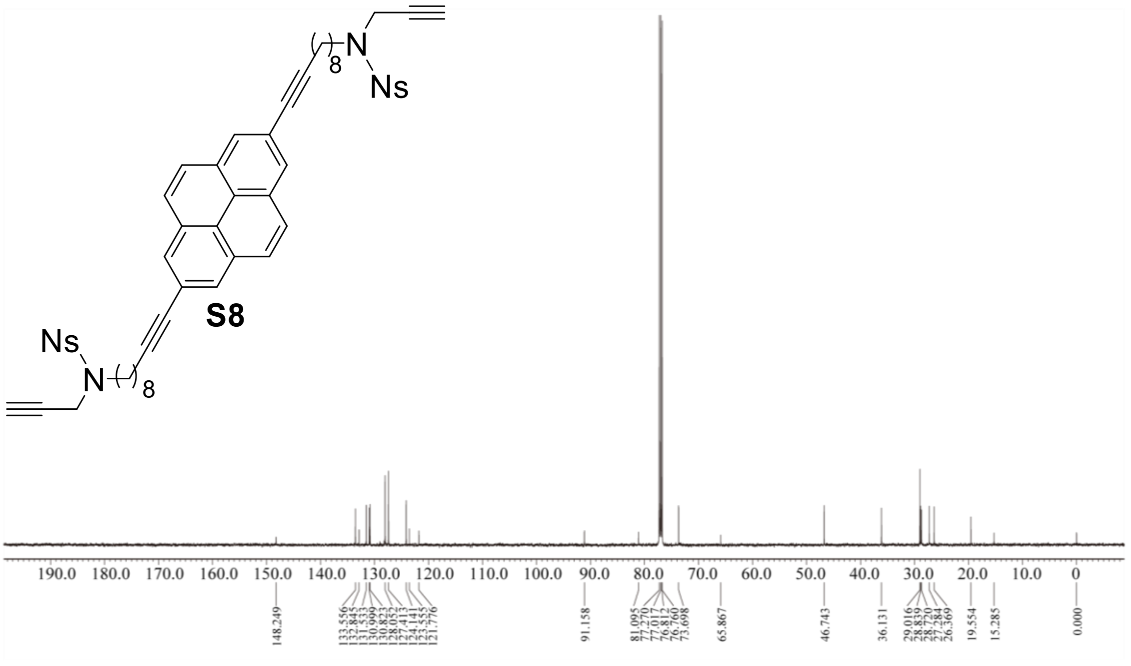

ESI-MS spectra of **S8**.

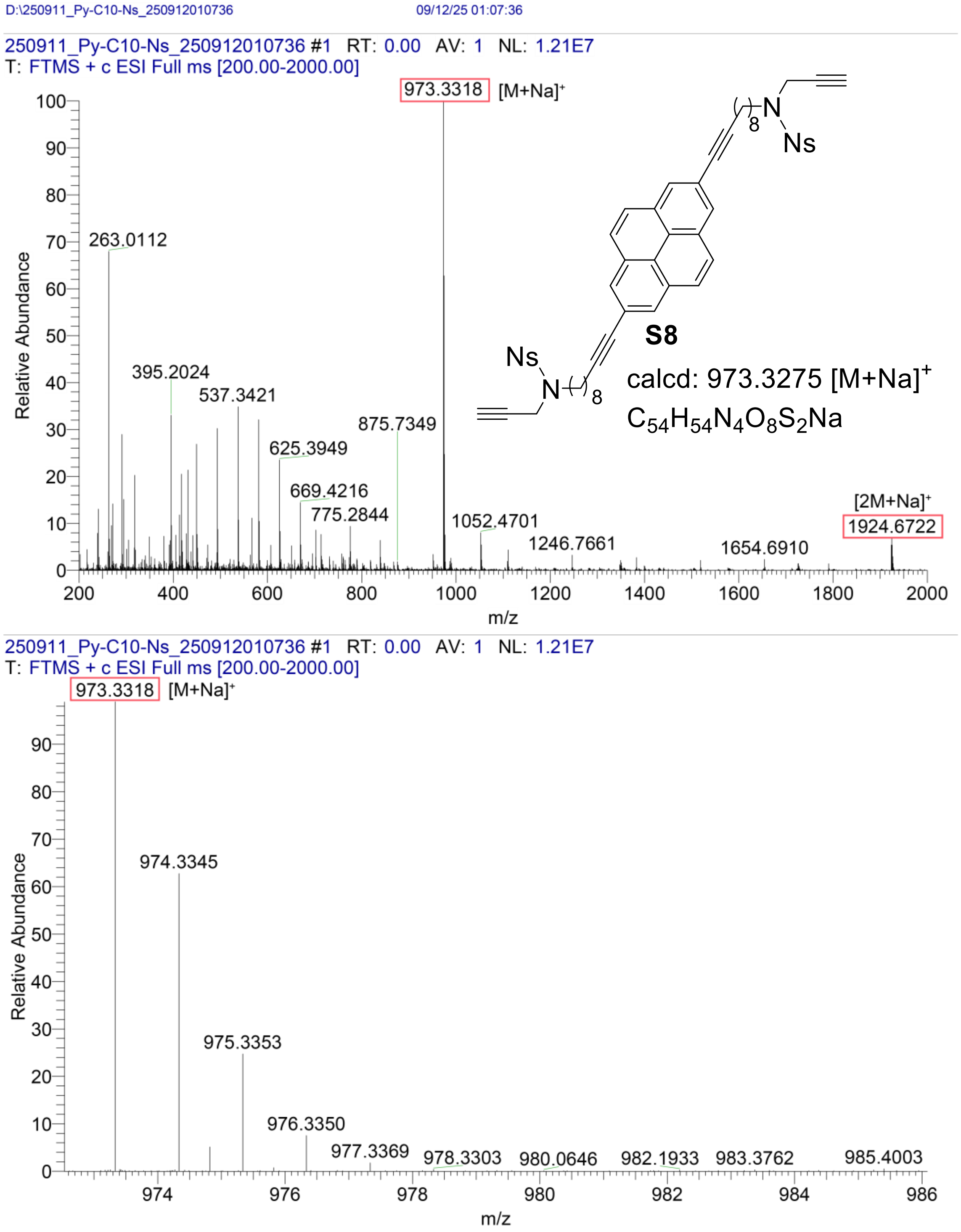

^{1}H NMR (500 MHz, CD_3OD) of **Axis-C10**.

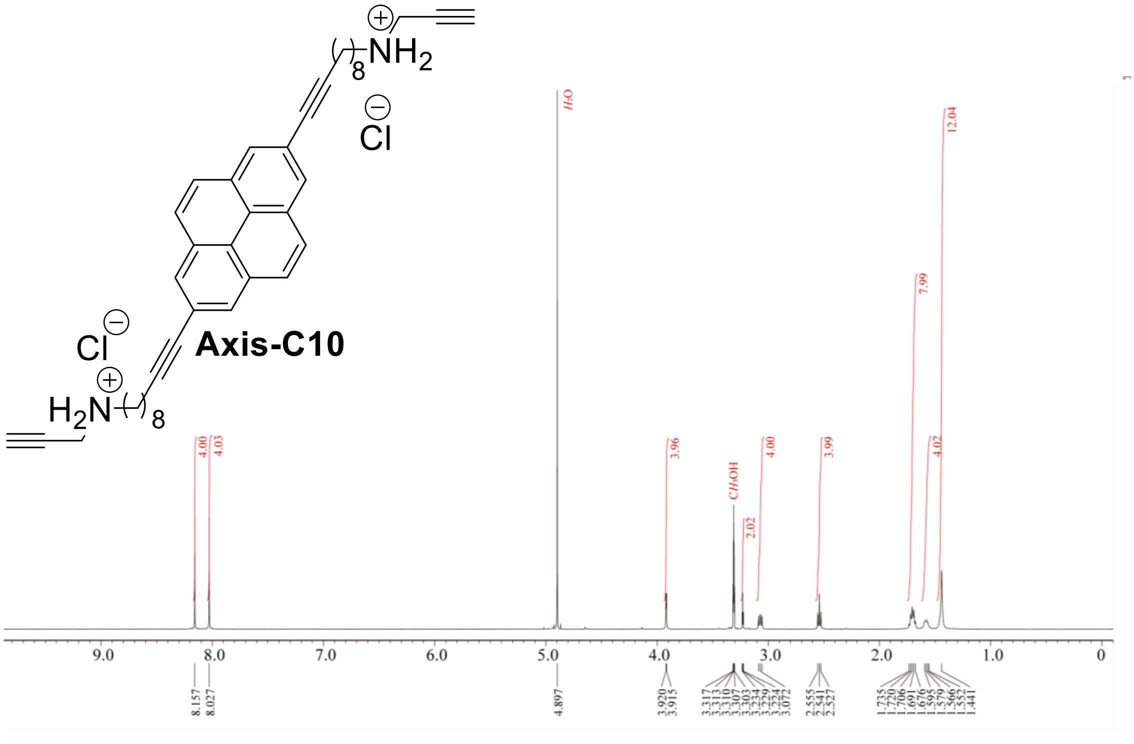

^{13}C NMR (125 MHz, CD_3OD) of **Axis-C10**.

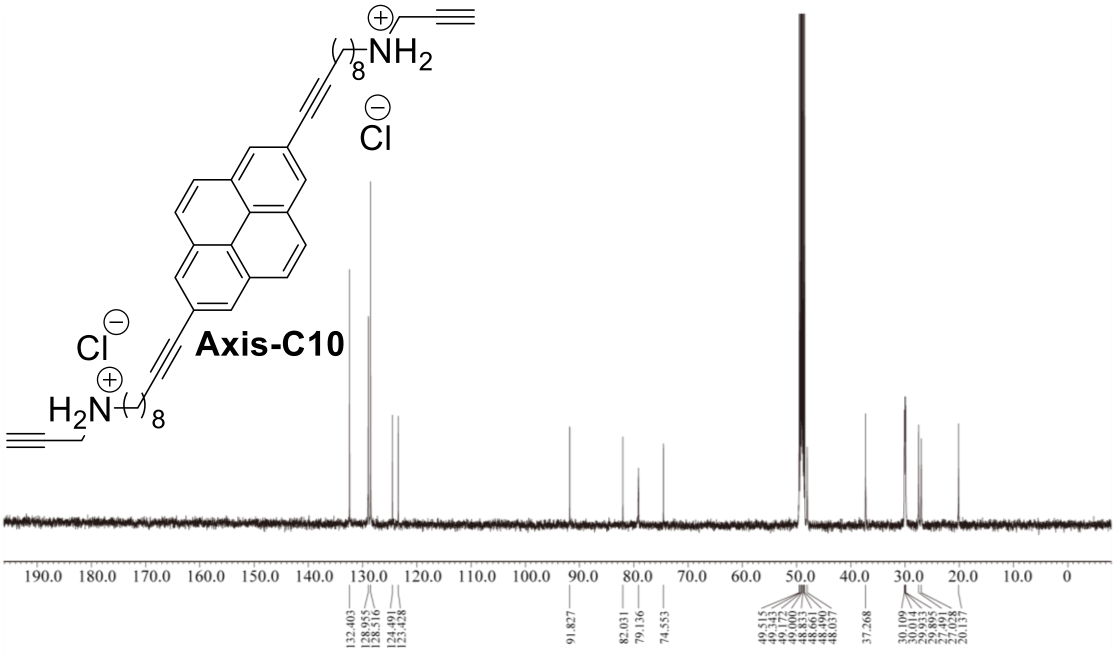

ESI-MS spectra of **Axis-C10**.

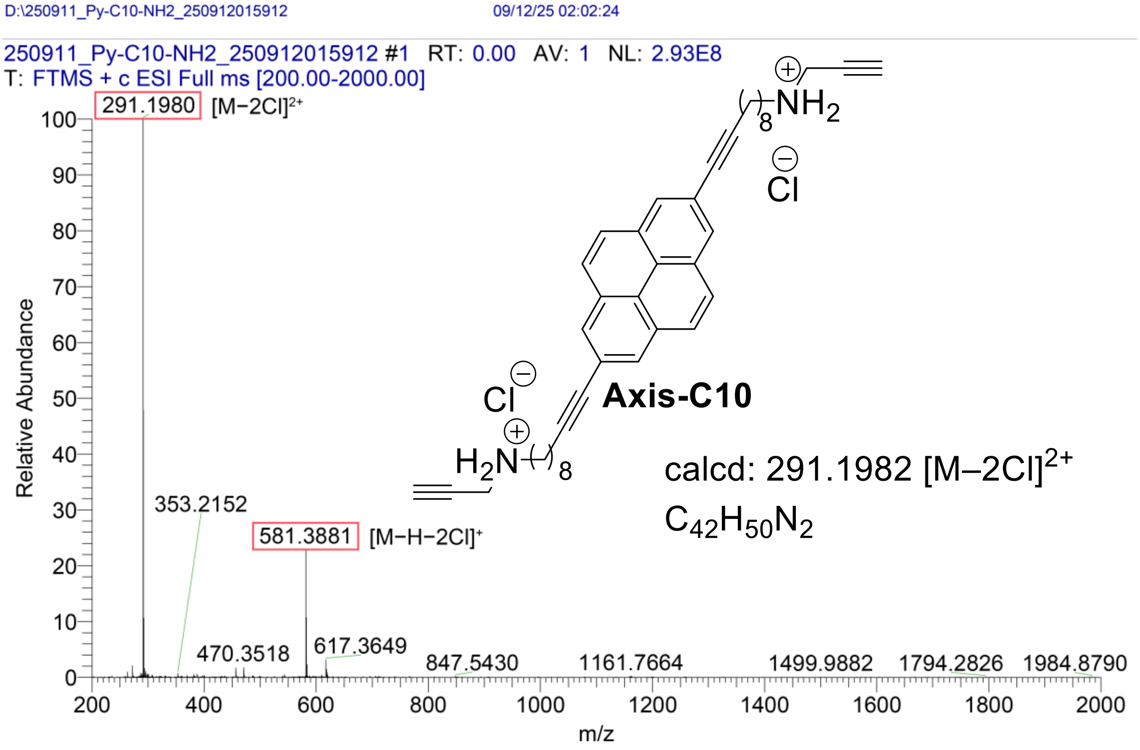

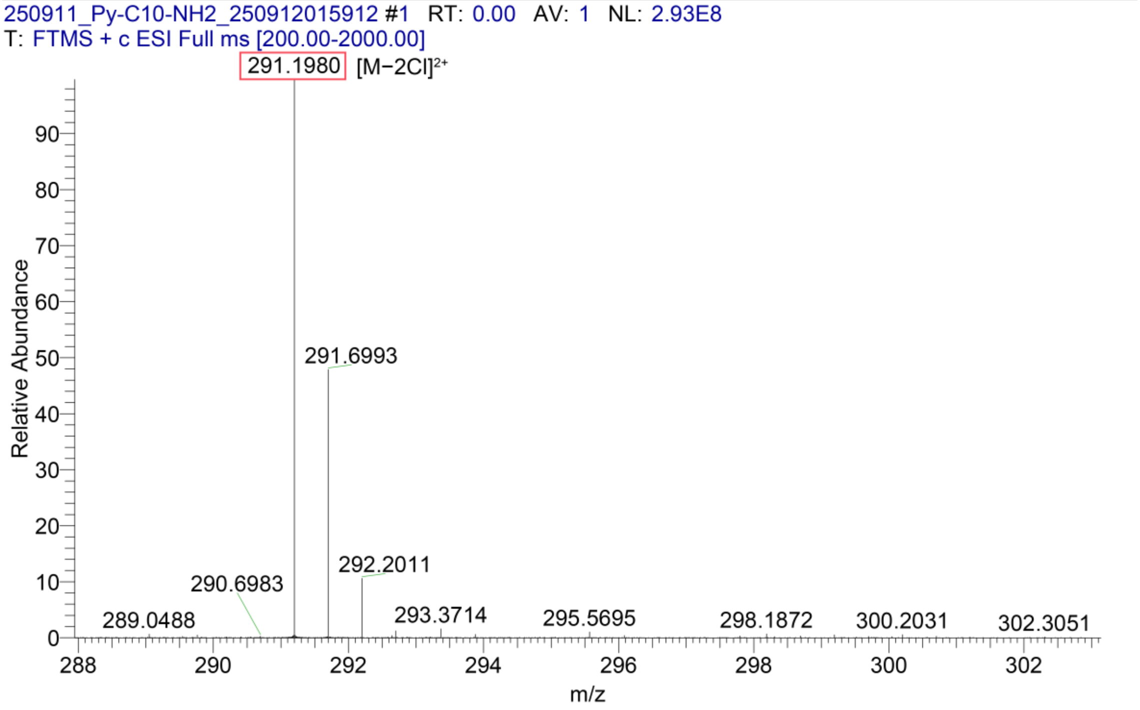

^{1}H NMR (500 MHz, D_2O) of **Rota-C10**.

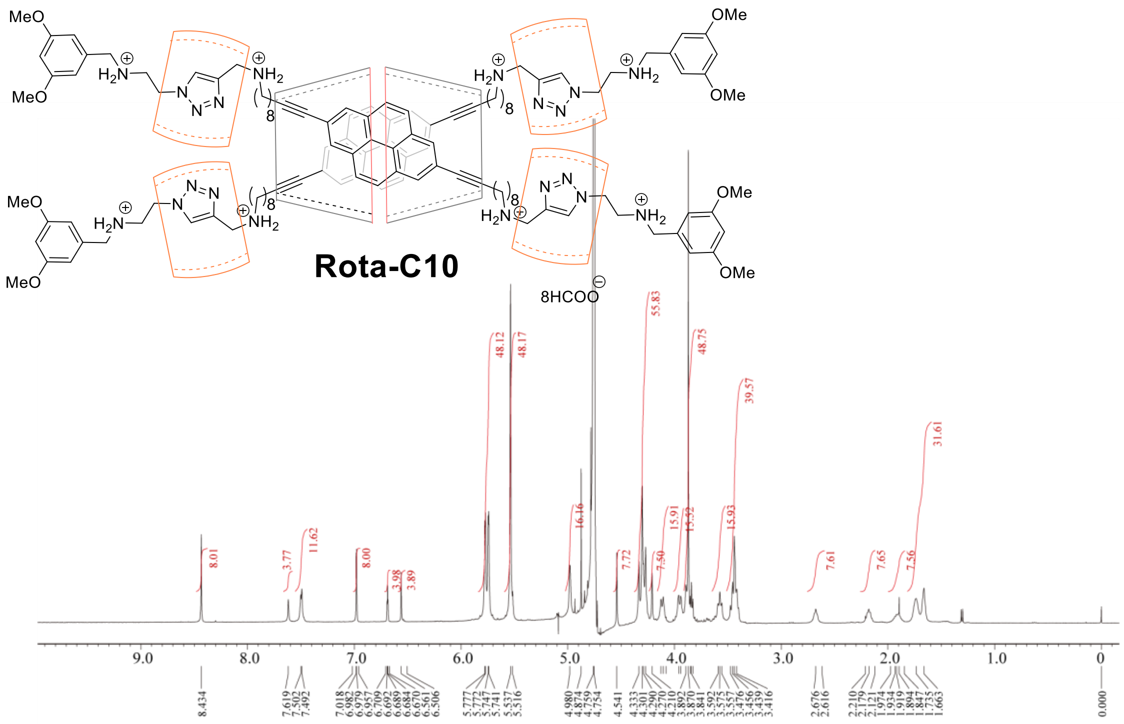

^{13}C NMR (125 MHz, D_2O) of **Rota-C10**.

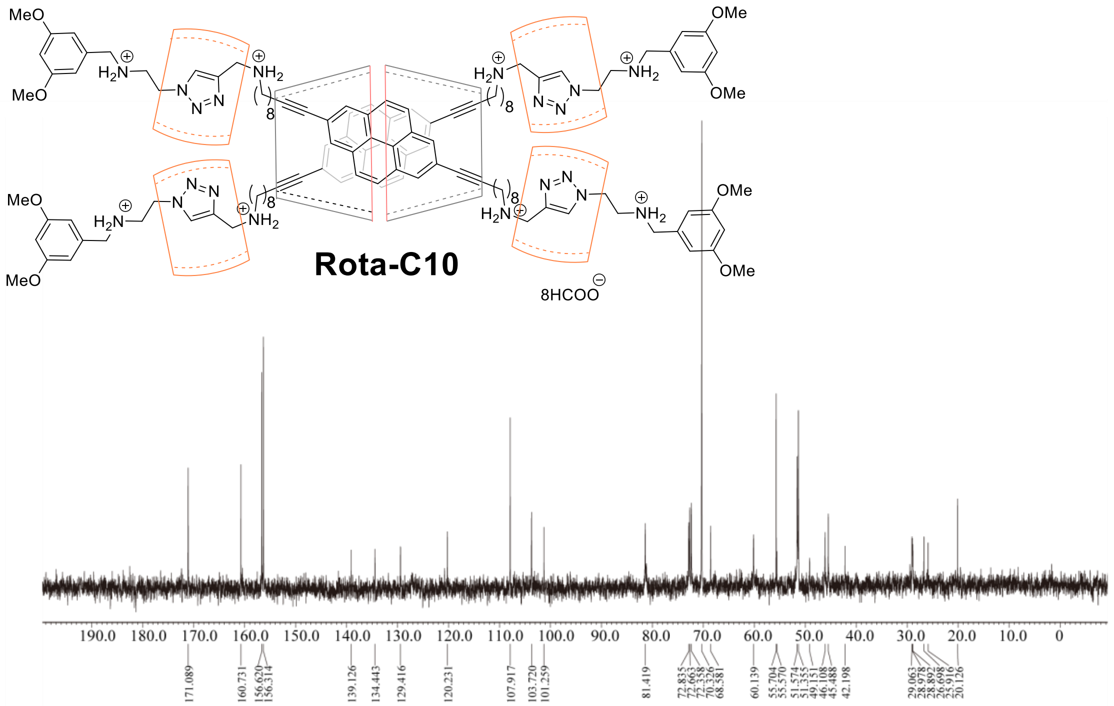

ESI-MS spectra of **Rota-C10**.

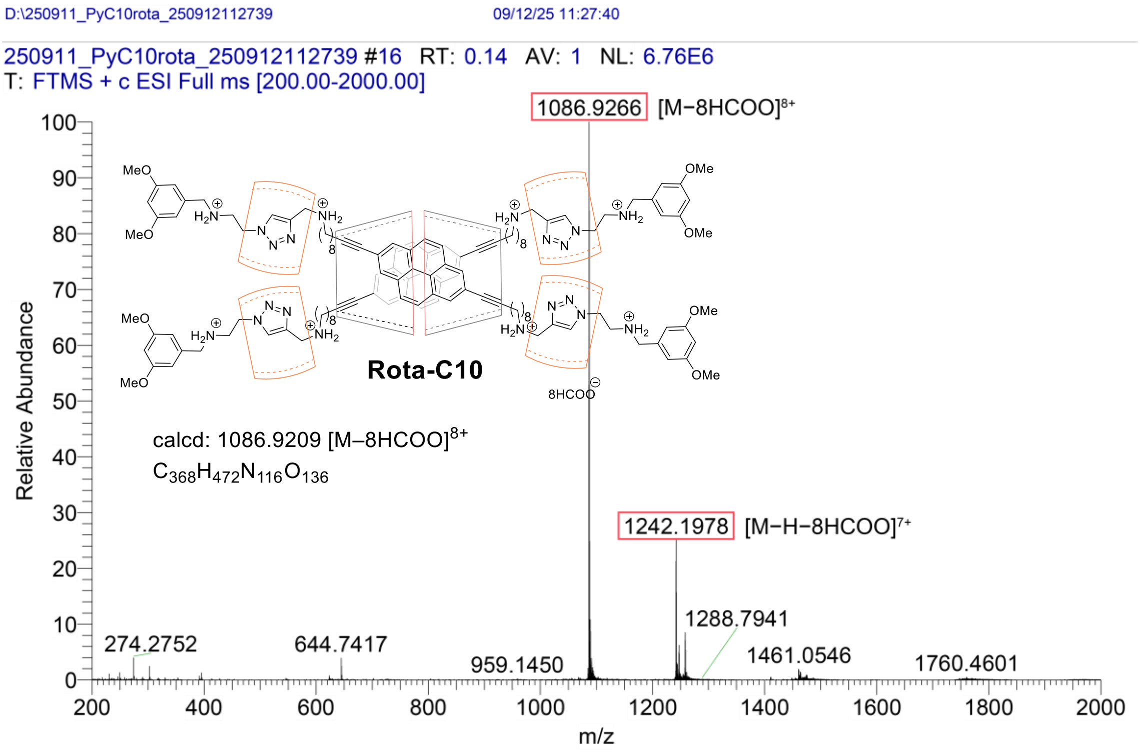

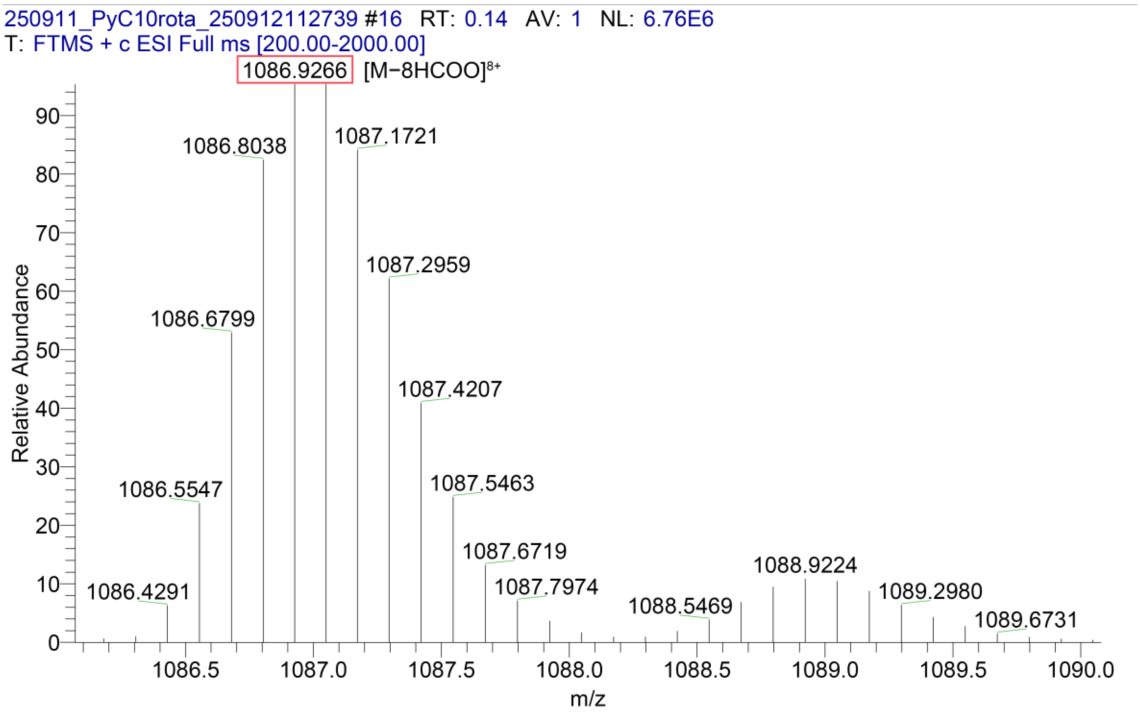

^{1}H NMR (500 MHz, D_2O) of reference compound **S9**.

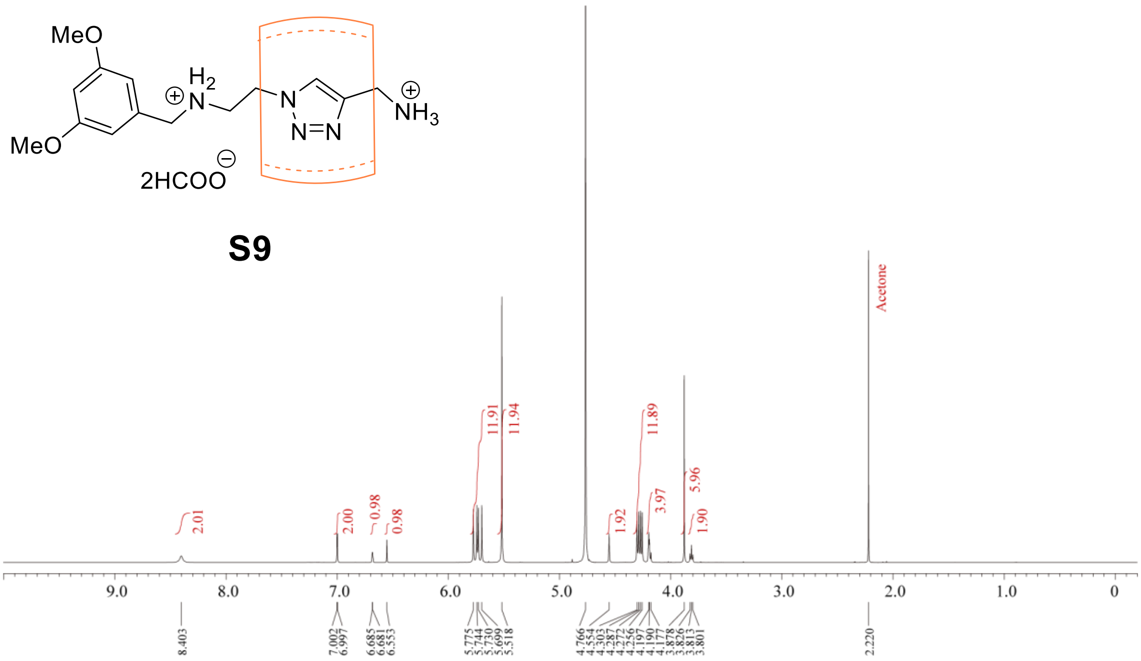

^{13}C NMR (125 MHz, D_2O) of reference compound **S9**.

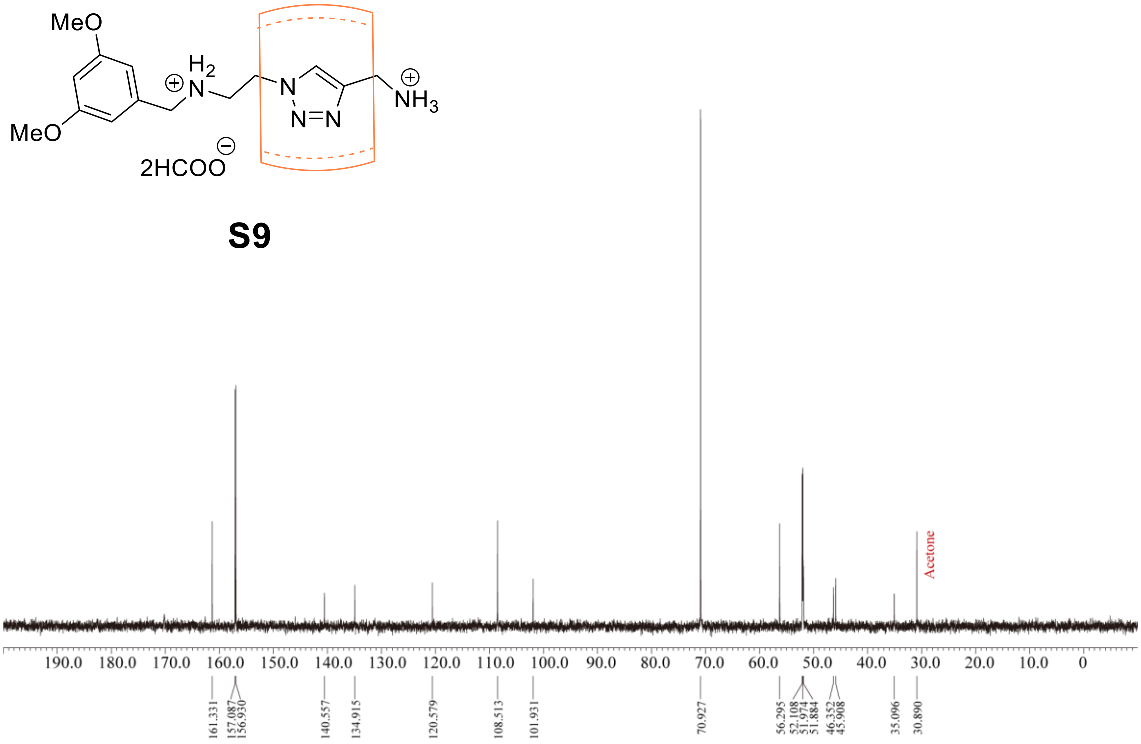

ESI-MS spectra of reference compound **S9**.

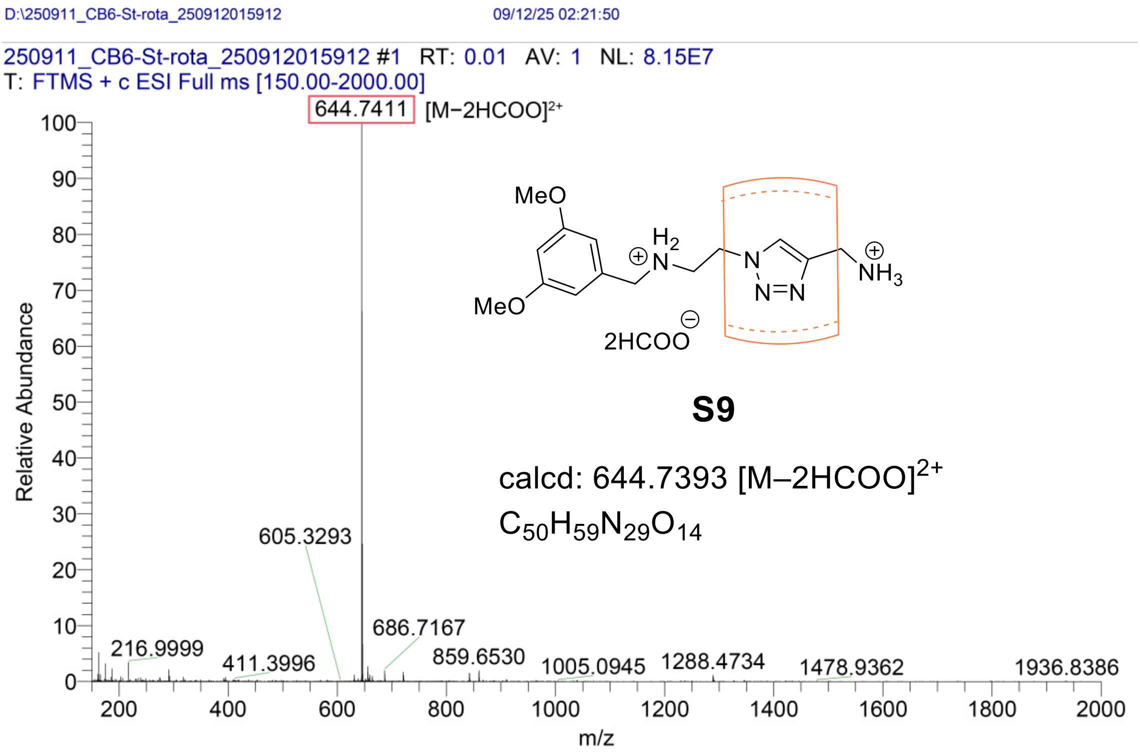

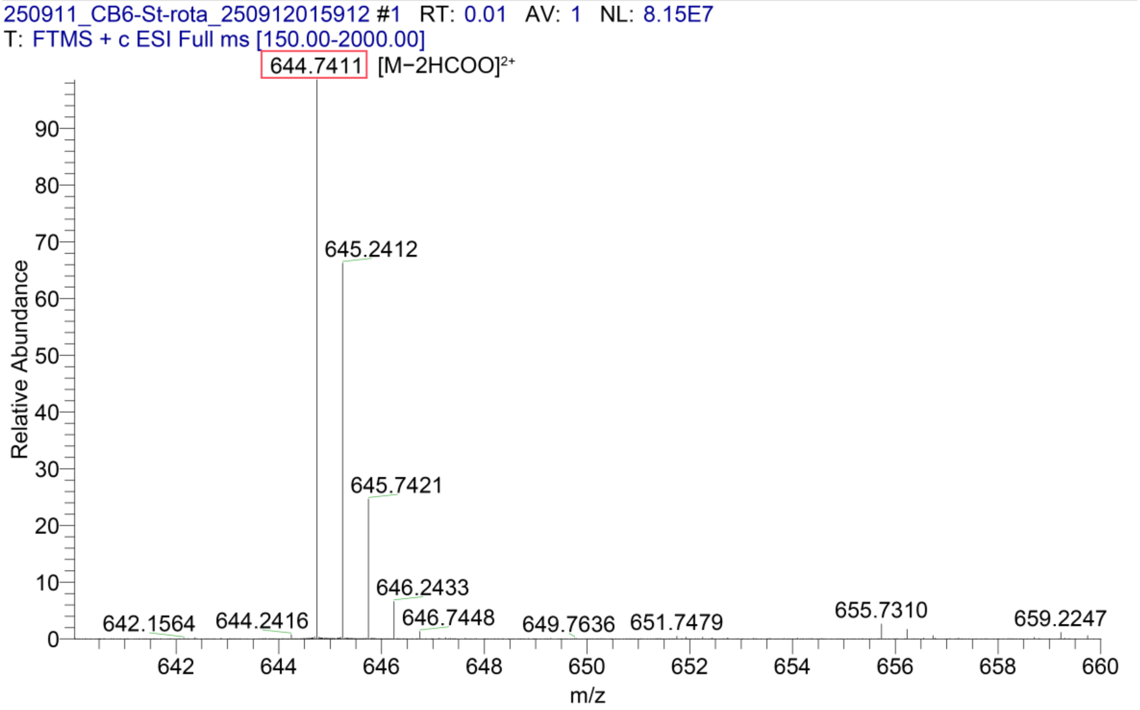

Supplement: SC-016-D5SC06304C-s001 [file SC-016-D5SC06304C-s001.pdf]
